# Supplementary material for: Investigating the stratified efficacy and safety of pharmacological blood pressure-lowering: an overall protocol for individual patient-level data meta-analyses of over 300 000 randomised participants in the new phase of the Blood Pressure Lowering Treatment Trialists’ Collaboration (BPLTTC)
Source: BMJ Open. 2019 May 22;9(5):e028698. doi: 10.1136/bmjopen-2018-028698 (PMC6538087; doi:10.1136/bmjopen-2018-028698)
Supplement: Supplementary file 1 [file bmjopen-2018-028698supp001.docx]

SUPPLEMENTARY MATERIAL

Investigating the stratified efficacy and safety of pharmacological blood pressure-lowering: An overall protocol for individual patient-level data meta-analyses of over 300,000 randomised participants in the new phase of the Blood Pressure Lowering Treatment Trialists’ Collaboration (BPLTTC)

Kazem Rahimi,^1^ Dexter Canoy,^1,2^ Milad Nazarzadeh,^1,3^ Gholamreza Salimi-Khorshidi,^1^ Mark Woodward,^1,4^ Koon Teo,^5^ Barry R Davis,^6^ John Chalmers,^4^ Carl J Pepine,^7^ on behalf of the Blood Pressure Lowering Treatment Trialists’ Collaboration

^1^The George Institute for Global Health, University of Oxford, Oxford, UK

^2^ Faculty of Medicine, University of New South Wales, Sydney, Australia

^3^ Collaboration Center of Meta-Analysis Research, Torbat Heydariyeh University of Medical Sciences, Torbat Heydariyeh, Iran

^4^The George Institute for Global Health, Sydney, New South Wales, Australia

^5^Population Health Research Institute, McMaster University, Hamilton, Ontario, Canada

^6^The University of Texas School of Public Health, Houston, Texas, USA

^7^College of of Medicine, University of Florida, Gainesville, Florida, USA

E-mail address:

kazem.rahimi@georgeinstitute.ox.ac.uk (KR)

dexter.canoy@georgeinstitute.ox.ac.uk (DC)

milad.nazarzadehlarzjan@georgeinstitute.ox.ac.uk (MN)

reza.khorshidi@georgeinstitute.ox.ac.uk (GSK)

mark.woodward@georgeinstitute.ox.ac.uk (MW)

Koon.Teo@phri.ca (KT)

Barry.R.Davis@uth.tmc.edu (BRD)

chalmers@georgeinstitute.org.au (JC)

Carl.Pepine@medicine.ufl.edu (CP)

***Supplementary table*** ***1.*** Search strategy to be used in MEDLINE, covering indexed entries between 01 Jan 1966 to 01 Jun 2018.

| Search (((((( "Hypertension/drug effects"[Mesh] OR "Hypertension/drug therapy"[Mesh] ))) AND (( "Blood Pressure/drug effects"[Mesh] OR "Blood Pressure/therapy"[Mesh] ))) AND ( ( Clinical Trial[ptyp] OR Controlled Clinical Trial[ptyp] OR Meta-Analysis[ptyp] OR Randomized Controlled Trial[ptyp] OR Clinical Trial, Phase III[ptyp] ) AND Humans[Mesh] AND adult[MeSH]))) AND ((((((((((("Antihypertensive Agents" [Pharmacological Action]) OR "Antihypertensive Agents/therapeutic use"[Mesh])) OR ((("Vasodilator Agents" [Pharmacological Action])) OR ( "Vasodilator Agents/therapeutic use"[Mesh] OR "Vasodilator Agents/therapy"[Mesh] ))) OR (("Adrenergic alpha-Antagonists/therapeutic use"[Mesh]) OR "Adrenergic alpha-Antagonists" [Pharmacological Action])) OR (("Adrenergic beta-Antagonists" [Pharmacological Action]) OR "Adrenergic beta-Antagonists/therapeutic use"[Mesh])) OR (("Sodium Chloride Symporter Inhibitors" [Pharmacological Action]) OR "Sodium Chloride Symporter Inhibitors/therapeutic use"[Mesh])) OR "Angiotensin-Converting Enzyme Inhibitors/therapeutic use"[Mesh]) OR (("Angiotensin II Type 1 Receptor Blockers" [Pharmacological Action]) OR "Angiotensin II Type 1 Receptor Blockers/therapeutic use"[Mesh])) OR (("Calcium Channel Blockers" [Pharmacological Action]) OR "Calcium Channel Blockers/therapeutic use"[Mesh])) AND ( ( Clinical Trial[ptyp] OR Controlled Clinical Trial[ptyp] OR Meta-Analysis[ptyp] OR Randomized Controlled Trial[ptyp] OR Clinical Trial, Phase III[ptyp] ) AND Humans[Mesh] AND adult[MeSH])) Filters: Clinical Trial; Controlled Clinical Trial; Randomized Controlled Trial; Clinical Trial, Phase III; Meta-Analysis; Systematic Reviews; Humans; Adult: 19+ years |
| --- |

***Supplementary table 2***. PRISMA-P (Preferred Reporting Items for Systematic review and Meta-Analysis Protocols) checklist for the Blood Pressure Lowering Treatment Trialists' Collaboration protocol for individual patient-level data meta-analysis

| Section and topic | Item No | Checklist item | | Location in the manuscript |
| --- | --- | --- | --- | --- |
| ADMINISTRATIVE INFORMATION |  | |  |  |
| Title: |  |  | |  |
| Identification | 1a | Identify the report as a protocol of a systematic review | | Title page |
| Update | 1b | If the protocol is for an update of a previous systematic review, identify as such | | NA |
| Registration | 2 | If registered, provide the name of the registry (such as PROSPERO) and registration number | | Page 5 |
| Authors: |  |  | |  |
| Contact | 3a | Provide name, institutional affiliation, e-mail address of all protocol authors; provide physical mailing address of corresponding author | | Title page (email address in Supplementary material title page) |
| Contributions | 3b | Describe contributions of protocol authors and identify the guarantor of the review | | Page 10 |
| Amendments | 4 | If the protocol represents an amendment of a previously completed or published protocol, identify as such and list changes; otherwise, state plan for documenting important protocol amendments | | NA |
| Support: |  |  | |  |
| Sources | 5a | Indicate sources of financial or other support for the review | | Page 10 |
| Sponsor | 5b | Provide name for the review funder and/or sponsor | | Page 10 |
| Role of sponsor or funder | 5c | Describe roles of funder(s), sponsor(s), and/or institution(s), if any, in developing the protocol | | Page 10 |
| INTRODUCTION |  | |  |  |
| Rationale | 6 | Describe the rationale for the review in the context of what is already known | | Page 3 |
| Objectives | 7 | Provide an explicit statement of the question(s) the review will address with reference to participants, interventions, comparators, and outcomes (PICO) | | Page 4 |
| METHODS |  | |  |  |
| Eligibility criteria | 8 | Specify the study characteristics (such as PICO, study design, setting, time frame) and report characteristics (such as years considered, language, publication status) to be used as criteria for eligibility for the review | | Pages 4 to 5 |
| Information sources | 9 | Describe all intended information sources (such as electronic databases, contact with study authors, trial registers or other grey literature sources) with planned dates of coverage | | Page 5 |
| Search strategy | 10 | Present draft of search strategy to be used for at least one electronic database, including planned limits, such that it could be repeated | | Supplementary table 1 |
| Study records: |  |  | |  |
| Data management | 11a | Describe the mechanism(s) that will be used to manage records and data throughout the review | | Page 5 |
| Selection process | 11b | State the process that will be used for selecting studies (such as two independent reviewers) through each phase of the review (that is, screening, eligibility and inclusion in meta-analysis) | | Pages 5 to 6 |
| Data collection process | 11c | Describe planned method of extracting data from reports (such as piloting forms, done independently, in duplicate), any processes for obtaining and confirming data from investigators | | Page 6 |
| Data items | 12 | List and define all variables for which data will be sought (such as PICO items, funding sources), any pre-planned data assumptions and simplifications | | Supplementary table 3 (Protocols for each meta-analysis will be developed and published which will include specific definitions of relevant variables) |
| Outcomes and prioritization | 13 | List and define all outcomes for which data will be sought, including prioritization of main and additional outcomes, with rationale | | For each meta-analysiss (to address each specific objectives), specific protocol will be developed and published which will include a definition of the outcome of interest |
| Risk of bias in individual studies | 14 | Describe anticipated methods for assessing risk of bias of individual studies, including whether this will be done at the outcome or study level, or both; state how this information will be used in data synthesis | | Page 6 |
| Data synthesis | 15a | Describe criteria under which study data will be quantitatively synthesised | | Pages 8 to 9 |
|  | 15b | If data are appropriate for quantitative synthesis, describe planned summary measures, methods of handling data and methods of combining data from studies, including any planned exploration of consistency (such as I^2^, Kendall’s τ) | | Pages 8 to 9 (more detailed analytical plans will be described in separate protocols for each meta-analysis) |
|  | 15c | Describe any proposed additional analyses (such as sensitivity or subgroup analyses, meta-regression) | | Pages 8 to 9 |
|  | 15d | If quantitative synthesis is not appropriate, describe the type of summary planned | | NA |
| Meta-bias(es) | 16 | Specify any planned assessment of meta-bias(es) (such as publication bias across studies, selective reporting within studies) | | Pages 6 and 9 (specific assessments will be described in the protocol for each planned meta-analysis) |
| Confidence in cumulative evidence | 17 | Describe how the strength of the body of evidence will be assessed (such as GRADE) | | Page 9 |

Reference: Shamseer L, Moher D, Clarke M, Ghersi D, Liberati A, Petticrew M, Shekelle P, Stewart L, PRISMA-P Group. Preferred reporting items for systematic review and meta-analysis protocols (PRISMA-P) 2015: elaboration and explanation. BMJ. 2015;349:g7647.

***Supplementary table 3***. Blood pressure lowering randomized trials eligible for BPLTTC individual patient-level data meta-analysis.

| Study name or author | Publication year | Total N | Intervention/Comparisons |
| --- | --- | --- | --- |
| **AASK** (African American Study of Kidney Disease and Hypertension)^1^ | 2006 | 1094 | Intensive BP lowering, moderate BP lowering, ACEi, CCB and BB (factorial design) |
| **ABCD** (Appropriate Blood Pressure Control in Diabetes Trial)^2^ | 1998 | 1900 | BP lowering to different targets |
| **ACCOMPLISH** (Avoiding Cardiovascular events through Combination therapy in Patients Living with Systolic Hypertension)^3^ | 2008 | 11,506 | CCB + ACEi vs diuretic |
| **ACCORD** (Action to Control Cardiovascular Risk in Diabetes)^4^ | 2010 | 4733 | Intensive vs standard BP lowering |
| **ACTION** (A Coronary Disease Trial Investigating Outcome with Nifedipine GITS)^5^ | 2004 | 7665 | CCB vs placebo |
| **ACTIVE I** (Atrial Fibrillation Clopidogrel Trial with Irbesartan for Prevention of Vascular Events)^6^ | 2011 | 9016 | AARB vs placebo |
| **ADVANCE** (Action in Diabetes and Vascular Disease)^7^ | 2007 | 11,140 | ACEi + diuretic vs placebo |
| **ALLHAT** (Antihypertensive and Lipid Lowering Treatment to Prevent Heart Attacks Trial)^8^ | 2002 | 42,418 | Diuretic vs CCB vs ACEi vs alpha-adrenergic blocker |
| **ALTITUDE** (Aliskiren Trial in Type 2 Diabetes Using Cardiovascular and Renal Disease Endpoints)^9^ | 2012 | 8561 | Renin inhibitor vs placebo |
| **ANBP** (The Australian National Blood Pressure Study)^10^ | 1980 | 3427 | Diuretic ± alpha-adrenergic agonist/BB vs placebo |
| **ANBP2** (Second Australian National Blood Pressure Study)^11^ | 2003 | 6083 | ACEi vs diuretic |
| **APSIS** (Angina Prognosis Study in Stockholm)^12^ | 1996 | 809 | BB vs CCB |
| **ASCOT-BPLA** (Anglo-Scandinavian Cardiac Outcomes Trial - Blood Pressure Lowering Arm)^13^ | 2005 | 19,257 | BB vs CCB |
| **ATTEMPT-CVD** (A Trial of Telmisartan Prevention of Cardiovascular Diseases)^14^ | 2016 | 1228 | ARB vs non-ARB |
| **BBB** (Behandla Blodtryck Battre)^15^ | 1994 | 2127 | Intensive vs usual BP lowering |
| **BCAPS** (β-Blocker Cholesterol-Lowering Asymptomatic Plaque Study)^16^ | 2001 | 793 | BB vs placebo |
| **BENEDICT** (Bergamo Nephrologic Diabetes Complications Trial)^17^ | 2004 | 1808 | ACEi, CCB or combination vs placebo; ACEi vs CCB |
| **CAMELOT** (The Comparison of Amlodipine vs Enalapril to Limit Occurrences of Thrombosis)^18^ | 2004 | 3327 | CCB or ACEi vs placebo; CCB vs ACEi |
| **CAPPP** (Captopril Prevention Project)^19^ | 1999 | 10,985 | ACEi vs diuretic + BB |
| **Cardio-Sis** (CARDIOvascolari del Controllo della Pressione Arteriosa SIStolica)^20^ | 2009 | 1111 | Tight vs usual BP lowering |
| **CASE-J** (Candesartan Antihypertensive Survival Evaluation in Japan)^21^ | 2008 | 4703 | ARB vs CCB |
| **CHIEF** (Chinese Hypertension Intervention Efficacy study)^22^ | 2012 | 13,080 | CCB + diuretic vs CCB + ARB |
| **COLM** (Combination of OLMesartan and calcium channel blocker or diuretic)^23^ | 2014 | 5141 | CCB + ARB vs diuretic + ARB |
| **CONVINCE** (Controlled Onset Verapamil Investigation of Cardiovascular End Points)^24^ | 2003 | 16,476 | CCB vs BB or diuretic |
| **Coope J**, et al^25^ | 1986 | 884 | BB ± diuretic |
| **COPE** (Combination Therapy of Hypertension to Prevent Cardiovascular Events)^26^ | 2011 | 6586 | BB + CCB vs ARB + CCB; ARB + CCB vs diuretic + CCB; BB + CCB vs diuretic + CCB |
| **DIABHYCAR** (Non-insulin-dependent diabetes, hypertension, microalbuminuria or proteinuria, cardiovascular events, and ramipril)^27^ | 2004 | 4912 | ACEi vs placebo |
| **DIME** (Diuretics in the Management of Essential hypertension study)^28^ | 2014 | 1130 | Diuretic vs non-diuretic |
| **DIRECT-Prevent 1** (Effect of candesartan on prevention)^29^ | 2008 | 1421 | ARB vs placebo |
| **DIRECT-Protect 1** (Effect of candesartan on progression)^29^ | 2008 | 1905 | ARB vs placebo |
| **DIRECT-Protect 2** (Effect of candesartan on progression and regression of retinopathy in type 2 diabetes)^30^ | 2011 | 1905 | ARB vs placebo |
| **DREAM** (Diabetes REduction Assessment with ramipril and rosiglitazone Medication)^31^ | 2006 | 5269 | ACEi vs placebo |
| **Dutch TIA Trial** (Dutch Transient Ischemic Attack Trial)^32^ | 1993 | 1473 | BB vs placebo |
| **E-COST** (Efficacy of Candesartan on Outcome in Saitama Trial)^33^ | 2005 | 2048 | ARB vs conventional drug (other than ACEi or ARB) |
| ELSA (Efficacy of Candesartan on Outcome in Saitama Trial)^34^ | 2002 | 2334 | BB vs CCB |
| **EUROPA** (European trial on reduction of cardiac events with perindopril in stable coronary artery)^35^ | 2003 | 12,218 | ACEi vs placebo |
| **EWPHE** (European Working Party on High Blood Pressure in the Elderly)^36^ | 1985 | 840 | Diuretic vs placebo |
| **FEVER** (Felodipine Event Reduction Study)^37^ | 2005 | 9711 | CCB + diuretic vs diuretic |
| **HAPPHY** (Heart Attack Primary Prevention in Hypertension Trial)^38^ | 1987 | 6569 | Diuretic vs BB |
| **HDFP** (Hypertension Detection and Follow Up Program)^39^ | 1979 | 10,940 | Intensive (stepped care) vs usual (referred care) BP lowering |
| **HIJ-CREATE** (Heart Institute of Japan Candesartan Randomized Trial for Evaluation in Coronary Heart Disease)^40^ | 2009 | 2049 | ARB vs non-ARB |
| **HOMED-BP** (Hypertension Objective Treatment based on Measurement by Electrical Devices of Blood Pressure Study)^41^ | 2012 | 3518 | Tight BP control, usual BP control, ACEi, CCB and ARB (factorial design) |
| **HOPE** (Heart Outcomes Prevention Evaluation Study)^42^ | 2000 | 9297 | ACEi vs placebo |
| **HOPE-3** (Heart Outcomes Prevention Evaluation-3)^43^ | 2016 | 12,705 | ARB + diuretic vs placebo |
| **HOT** (Hypertension Optimal Treatment Study)^44^ | 1998 | 18,790 | DBP lowering to ≤80, ≤85 and ≤90 mmHg |
| **HYVET** (Hypertension in the Very Elderly Trial)^45^ | 2008 | 3845 | Diuretic vs placebo |
| **IDNT** (Irbesartan Diabetic Nephropathy Trial)^46^ | 2001 | 2861 | ARB or CCB vs placebo; ARB vs CCB |
| **INSIGHT** (International Nifedipine GITS Study: Intervention as a Goal for Hypertension Therapy)^47^ | 2000 | 6321 | CCB vs diuretic |
| **INVEST** (International Verapamil-Trandolapril Study)^48^ | 2003 | 22,576 | CCB vs BB |
| **IPPSH** (International Prospective Primary Prevention Study in Hypertension)^49^ | 1985 | 6357 | BB vs non-BB |
| **JATOS** (Japanese Trial to Assess Optimal Systolic Blood Pressure in Elderly Hypertensive Patients)^50^ | 2008 | 4418 | Strict vs mild BP lowering |
| **JMIC-B** (Japan Multicenter Investigation for Cardiovascular Diseases-B)^51^ | 2004 | 1650 | CCB vs ACEi |
| **LIFE** (Losartan Intervention for Endpoint Reduction in Hypertension Study)^52^ | 2002 | 9193 | ARB vs BB |
| **LIT** (Lopressor Intervention Trial)^53^ | 1987 | 2395 | BB vs placebo |
| **MIDAS** (Multicenter Isradipine Diuretic Atherosclerosis Study)^54^ | 1996 | 883 | CCB vs diuretic |
| **MOSES** (Morbidity and Mortality After Stroke, Eprosartan Compared With Nitrendipine for Secondary Prevention)^55^ | 2005 | 1352 | ARB vs CCB |
| **MRC-1** (Medical Research Council Treatment of Mild Hypertension)^56^ | 1985 | 26,054 | Diuretic or BB vs placebo |
| **MRC-2** (Medical Research Council Treatment of Hypertension in Older Adults)^57^ | 1992 | 6579 | Diuretic or BB vs placebo; Diuretic vs BB |
| **Multicentre International Study**^58^ | 1975 | 3038 | BB vs placebo |
| **NAVIGATOR** (Nateglinide and Valsartan in Impaired Glucose Tolerance Outcomes Research)^59^ | 2010 | 9306 | ARB vs placebo |
| **NICOLE** (Nisoldipine in Coronary Artery Disease in Leuven Study)^60^ | 2001 | 819 | CCB vs placebo |
| **NICS-EH** (National Intervention Cooperative Study in Elderly Hypertensives)^61^ | 1999 | 414 | CCB vs diuretic |
| **NORDIL** (Nordic Diltiazem Study)^62^ | 2000 | 10,881 | CCB vs diuretic |
| **ONTARGET** (Ongoing Telmisartan Alone and in Combination with Ramipril Global Endpoint Trial)^63^ | 2008 | 34,196 | ARB + ACEi vs ACEi; ARB vs ACEi |
| **OSCAR** (OlmeSartan and calcium antagonists randomized study)^64^ | 2012 | 1164 | ARB vs ARB + CCB |
| **The Oslo Study**^65^ | 1980 | 785 | Diuretic ± alpha-adrenergic agonist or BB vs placebo |
| **PART-2** (Prevention of Atherosclerosis with Ramipril Trial)^66^ | 2000 | 617 | ACEi vs placebo |
| **PATE-Hypertension** (The Practitioner’s Trial on the Efficacy of Antihypertensive Treatment in the Elderly with Hypertension)^67^ | 2000 | 1748 | ACEi vs CCB |
| **PATS** (Post-stroke Antihypertensive Treatment Study)^68^ | 2009 | 5665 | Diuretic vs placebo |
| **PEACE** (Prevention of Events with Angiotensin Converting Enzyme Inhibition)^69^ | 2004 | 8290 | ACEi vs placebo |
| **PHARAO** (Prevention of hypertension with the angiotensin-converting enzyme inhibitor ramipril in patients with high-normal blood pressure)^70^ | 2008 | 1008 | ACEi vs placebo |
| **PREVEND IT** (Prevention of Renal and Vascular Endstage Disease)^71^ | 2004 | 864 | ACEi vs placebo |
| **PREVENT** (Prospective Randomized Evaluation of the Vascular Effects of Norvasc Trial)^72^ | 2000 | 825 | CCB vs placebo |
| **PRoFESS** (Prevention Regimen For Effectively Avoiding Second Strokes)^73^ | 2008 | 20,332 | ARB vs placebo |
| **PROGRESS** (Perindopril Protection Against Recurrent Stroke Study)^74^ | 2001 | 6105 | ACEi ± diuretic vs placebo |
| **QUIET** (Quinapril Ischaemic Event Trial)^75^ | 2001 | 1750 | ACEi vs placebo |
| **RENAAL** (Reduction of Endpoints in NIDDM with the Angiotensin II Antagonist Losartan Study)^76^ | 2001 | 1513 | ARB vs placebo |
| **ROADMAP** (Randomized Olmesartan And Diabetes Microalbuminuria Prevention Study)^77^ | 2011 | 4447 | ARB vs placebo |
| **SCOPE** (Study on Cognition and Prognosis in the Elderly)^78^ | 2003 | 4937 | ARB vs placebo |
| **SHELL** (Systolic Hypertension in the Elderly Long-term Lacidipine Trial)^79^ | 2003 | 1882 | CCB vs diuretic |
| **SHEP** (Systolic Hypertension in the Elderly Program)^80^ | 1991 | 4736 | Diuretic ± BB vs placebo |
| **SPRINT** (Systolic Blood Pressure Intervention Trial)^81^ | 2015 | 9361 | Intensive vs standard BP lowering |
| **SPS3** (Secondary Prevention of Small Subcortical Strokes)^82^ | 2013 | 3020 | SBP lowering to <120 and <140 mmHg |
| **STONE** (Shanghai trial of nifedipine in the elderly)^83^ | 1996 | 1632 | CCB vs placebo |
| **STOP Hypertension** (Swedish Trial in Old Patients with Hypertension)^84^ | 1991 | 1627 | BB + diuretic vs placebo |
| **STOP Hypertension-2** (Swedish Trial in Old Patients with Hypertension-2)^85^ | 1999 | 13,228 | ACEi vs conventional drug; CCB vs conventional drug; ACEi vs CCB |
| **Sun M**, et al^86^ | 1997 | 2080 | CCB vs placebo |
| **Syst-China** (Systolic Hypertension in China)^87^ | 1988 | 2394 | CCB ± ACEi and/or diuretic vs placebo |
| **Syst-Eur** (Systolic Hypertension in Europe)^88^ | 1997 | 4695 | CCB ± ACEi and/or diuretic |
| **Taylor SH**, et al^89^ | 1982 | 1103 | BB vs placebo |
| **TEST** (Ternormin after stroke and TIA)^90^ | 1995 | 720 | BB vs placebo |
| **TOMHS** (Treatment of Mild Hypertension Study)^91^ | 1993 | 902 | Multi-drug class combination vs placebo |
| **TRANSCEND** (Telmisartan Randomised Assessment Study in ACE Intolerant Subjects with Cardiovascular Disease)^92^ | 2008 | 5926 | ARB vs placebo |
| **TROPHY** (Trial of Preventing Hypertension)^93^ | 2006 | 772 | ARB vs placebo |
| **UKPDS** (UK Prospective Diabetes Study)^94^ | 1998 | 1906 | Strict vs less strict BP lowering; ACEi vs BB |
| **VA NEPHRON-D** (Veterans Affairs Nephropathy in Diabetes)^95^ | 2013 | 1448 | ACEi + ARB vs ARB |
| **VALISH** (Valsartan in Elderly Isolated Systolic Hypertension Study)^96^ | 2010 | 3079 | Strict vs moderate BP lowering |
| **VALUE** (Valsartan Antihypertensive Long-Term Use Evaluation)^97^ | 2004 | 15,245 | ARB vs CCB |
| **VHAS** (Verapamil in Hypertension and Atherosclerosis Study)^98^ | 1997 | 1414 | Diuretic vs CCB |
| **Wei Y**, et al.^99^ | 2013 | 724 | Intensive vs standard BP lowering |

BPLTTC – Blood Pressure Lowering Treatment Trialists' Collaboration; ACEi – Angiotensin-converting enzyme inhibitor; ARB – Angiotensin receptor blocker; BB – Beta-blocker; CCB – Calcium channel blocker; BP – blood pressure; SBP – Systolic BP; DBP – Diastolic BP.

**Supplementary table 3 references:**

1. Wright JT, Jr., Bakris G, Greene T, et al. Effect of blood pressure lowering and antihypertensive drug class on progression of hypertensive kidney disease: results from the AASK trial. *JAMA* 2002;**288**:2421-31.

2. Estacio RO, Jeffers BW, Hiatt WR, et al. The effect of nisoldipine as compared with enalapril on cardiovascular outcomes in patients with non-insulin-dependent diabetes and hypertension. *New Engl J Med* 1998;**338**:645-52. doi:10.1056/NEJM199803053381003

3. Jamerson K, Weber MA, Bakris GL, et al. Benazepril plus amlodipine or hydrochlorothiazide for hypertension in high-risk patients. *New Engl J Med* 2008;**359**:2417-28. doi:10.1056/NEJMoa0806182

4. ACCORD Study Group, Cushman WC, Evans GW, et al. Effects of intensive blood-pressure control in type 2 diabetes mellitus. *New Engl J Med* 2010;**362**:1575-85. doi:10.1056/NEJMoa1001286

5. Poole-Wilson PA, Lubsen J, Kirwan BA, et al. Effect of long-acting nifedipine on mortality and cardiovascular morbidity in patients with stable angina requiring treatment (ACTION trial): randomised controlled trial. *Lancet* 2004;**364**:849-57. doi:10.1016/S0140-6736(04)16980-8

6. Active I Investigators, Yusuf S, Healey JS, et al. Irbesartan in patients with atrial fibrillation. *New Engl J Med* 2011;**364**:928-38. doi:10.1056/NEJMoa1008816

7. Patel A, Group AC, MacMahon S, et al. Effects of a fixed combination of perindopril and indapamide on macrovascular and microvascular outcomes in patients with type 2 diabetes mellitus (the ADVANCE trial): a randomised controlled trial. *Lancet* 2007;**370**:829-40. doi:10.1016/S0140-6736(07)61303-8

8. Major cardiovascular events in hypertensive patients randomized to doxazosin vs chlorthalidone: the antihypertensive and lipid-lowering treatment to prevent heart attack trial (ALLHAT). ALLHAT Collaborative Research Group. *JAMA*  2000;**283**:1967-75.

9. Parving HH, Brenner BM, McMurray JJ, et al. Cardiorenal end points in a trial of aliskiren for type 2 diabetes. *New Engl J Med* 2012;**367**:2204-13. doi:10.1056/NEJMoa1208799

10. The Australian therapeutic trial in mild hypertension. Report by the Management Committee. *Lancet* 1980;**315**:1261-7.

11. Wing LM, Reid CM, Ryan P, et al. A comparison of outcomes with angiotensin-converting--enzyme inhibitors and diuretics for hypertension in the elderly. *New Engl J Med* 2003;**348**:583-92. doi:10.1056/NEJMoa021716

12. Rehnqvist N, Hjemdahl P, Billing E, et al. Effects of metoprolol vs verapamil in patients with stable angina pectoris. The Angina Prognosis Study in Stockholm (APSIS). *Eur Heart J* 1996;**17**:76-81.

13. Dahlöf B, Sever PS, Poulter NR, et al. Prevention of cardiovascular events with an antihypertensive regimen of amlodipine adding perindopril as required versus atenolol adding bendroflumethiazide as required, in the Anglo-Scandinavian Cardiac Outcomes Trial-Blood Pressure Lowering Arm (ASCOT-BPLA): a multicentre randomised controlled trial. *Lancet* 2005;**366**:895-906. doi:10.1016/s0140-6736(05)67185-1

14. Ogawa H, Soejima H, Matsui K, et al. A trial of telmisartan prevention of cardiovascular diseases (ATTEMPT-CVD): Biomarker study. *Eur J Prev Cardiol* 2016;**23**:913-21. doi:10.1177/2047487315603221

15. Hansson L. The BBB Study: the effect of intensified antihypertensive treatment on the level of blood pressure, side-effects, morbidity and mortality in well-treated hypertensive patients. Behandla Blodtryck Battre. *Blood Pressure* 1994;**3**:248--54.

16. Hedblad B, Wikstrand J, Janzon L, et al. Low-dose metoprolol CR/XL and fluvastatin slow progression of carotid intima-media thickness: Main results from the Beta-Blocker Cholesterol-Lowering Asymptomatic Plaque Study (BCAPS). *Circulation* 2001;**103**:1721-6.

17. Ruggenenti P, Fassi A, Ilieva AP, et al. Preventing microalbuminuria in type 2 diabetes. *New Engl J Med* 2004;**351**:1941-51. doi:10.1056/NEJMoa042167

18. Nissen SE, Tuzcu EM, Libby P, et al. Effect of antihypertensive agents on cardiovascular events in patients with coronary disease and normal blood pressure: the CAMELOT study: a randomized controlled trial. *JAMA* 2004;**292**:2217-25. doi:10.1001/jama.292.18.2217

19. Hansson L, Lindholm LH, Niskanen L, et al. Effect of angiotensin-converting-enzyme inhibition compared with conventional therapy on cardiovascular morbidity and mortality in hypertension: the Captopril Prevention Project (CAPPP) randomised trial. *Lancet* 1999;**353**:611-6.

20. Verdecchia P, Staessen JA, Angeli F, et al. Usual versus tight control of systolic blood pressure in non-diabetic patients with hypertension (Cardio-Sis): an open-label randomised trial. *Lancet* 2009;**374**:525-33. doi:10.1016/S0140-6736(09)61340-4

21. Ogihara T, Nakao K, Fukui T, et al. Effects of candesartan compared with amlodipine in hypertensive patients with high cardiovascular risks: candesartan antihypertensive survival evaluation in Japan trial. *Hypertension* 2008;**51**:393-8. doi:10.1161/HYPERTENSIONAHA.107.098475

22. Ma L, Wang W, Zhao Y, et al. Combination of amlodipine plus angiotensin receptor blocker or diuretics in high-risk hypertensive patients: a 96-week efficacy and safety study. *Am J Cardiovasc Drugs* 2012;**12**:137-42. doi:10.2165/11598110-000000000-00000

23. Ogihara T, Saruta T, Rakugi H, et al. Combinations of olmesartan and a calcium channel blocker or a diuretic in elderly hypertensive patients: a randomized, controlled trial. *J Hypertens* 2014;**32**:2054-63; discussion 63. doi:10.1097/HJH.0000000000000281

24. Black HR, Elliott WJ, Grandits G, et al. Principal results of the Controlled Onset Verapamil Investigation of Cardiovascular End Points (CONVINCE) trial. *JAMA* 2003;**289**:2073-82. doi:10.1001/jama.289.16.2073

25. Coope J, Warrender TS. Randomised trial of treatment of hypertension in elderly patients in primary care. *BMJ*  1986;**293**:1145-51.

26. Matsuzaki M, Ogihara T, Umemoto S, et al. Prevention of cardiovascular events with calcium channel blocker-based combination therapies in patients with hypertension: a randomized controlled trial. *J Hypertens* 2011;**29**:1649-59. doi:10.1097/HJH.0b013e328348345d

27. Marre M, Lievre M, Chatellier G, et al. Effects of low dose ramipril on cardiovascular and renal outcomes in patients with type 2 diabetes and raised excretion of urinary albumin: randomised, double blind, placebo controlled trial (the DIABHYCAR study). *BMJ* 2004;**328**:495. doi:10.1136/bmj.37970.629537.0D

28. Ueda S, Morimoto T, Ando S, et al. A randomised controlled trial for the evaluation of risk for type 2 diabetes in hypertensive patients receiving thiazide diuretics: Diuretics In the Management of Essential hypertension (DIME) study. *BMJ Open* 2014;**4**:e004576. doi:10.1136/bmjopen-2013-004576

29. Chaturvedi N, Porta M, Klein R, et al. Effect of candesartan on prevention (DIRECT-Prevent 1) and progression (DIRECT-Protect 1) of retinopathy in type 1 diabetes: randomised, placebo-controlled trials. *Lancet* 2008;**372**:1394-402. doi:10.1016/S0140-6736(08)61412-9

30. Tillin T, Orchard T, Malm A, et al. The role of antihypertensive therapy in reducing vascular complications of type 2 diabetes. Findings from the DIabetic REtinopathy Candesartan Trials-Protect 2 study. *J Hypertens* 2011;**29**:1457-62. doi:10.1097/HJH.0b013e3283480db9

31. DREAM Trial Investigators, Bosch J, Yusuf S, et al. Effect of ramipril on the incidence of diabetes. *New Engl J Med*  2006;**355**:1551-62. doi:10.1056/NEJMoa065061

32. Trial of secondary prevention with atenolol after transient ischemic attack or nondisabling ischemic stroke. The Dutch TIA Trial Study Group. *Stroke* 1993;**24**:543-8.

33. Suzuki H, Kanno Y, Group EoCoOiST. Effects of candesartan on cardiovascular outcomes in Japanese hypertensive patients. *Hypertens Res* 2005;**28**:307-14. doi:10.1291/hypres.28.307

34. Zanchetti A, Bond MG, Hennig M, et al. Calcium antagonist lacidipine slows down progression of asymptomatic carotid atherosclerosis: principal results of the European Lacidipine Study on Atherosclerosis (ELSA), a randomized, double-blind, long-term trial. *Circulation* 2002;**106**:2422-7.

35. Fox KM, Investigators EtOrocewPiscAd. Efficacy of perindopril in reduction of cardiovascular events among patients with stable coronary artery disease: randomised, double-blind, placebo-controlled, multicentre trial (the EUROPA study). *Lancet* 2003;**362**:782-8.

36. Amery A, Birkenhager W, Brixko P, et al. Mortality and morbidity results from the European Working Party on High Blood Pressure in the Elderly trial. *Lancet* 1985;**1**:1349-54.

37. Liu L, Zhang Y, Liu G, et al. The Felodipine Event Reduction (FEVER) Study: a randomized long-term placebo-controlled trial in Chinese hypertensive patients. *J Hypertens* 2005;**23**:2157-72.

38. Wilhelmsen L, Berglund G, Elmfeldt D, et al. Beta-blockers versus diuretics in hypertensive men: main results from the HAPPHY trial. *J Hypertens* 1987;**5**:561-72.

39. Five-year findings of the hypertension detection and follow-up program. I. Reduction in mortality of persons with high blood pressure, including mild hypertension. Hypertension Detection and Follow-up Program Cooperative Group. *JAMA* 1979;**242**:2562-71.

40. Kasanuki H, Hagiwara N, Hosoda S, et al. Angiotensin II receptor blocker-based vs. non-angiotensin II receptor blocker-based therapy in patients with angiographically documented coronary artery disease and hypertension: the Heart Institute of Japan Candesartan Randomized Trial for Evaluation in Coronary Artery Disease (HIJ-CREATE). *Eur Heart J* 2009;**30**:1203-12. doi:10.1093/eurheartj/ehp101

41. Asayama K, Ohkubo T, Metoki H, et al. Cardiovascular outcomes in the first trial of antihypertensive therapy guided by self-measured home blood pressure. *Hypertens Res* 2012;**35**:1102-10. doi:10.1038/hr.2012.125

42. Investigators; HOPES, Yusuf S, Sleight P, et al. Effects of an angiotensin-converting-enzyme inhibitor, ramipril, on cardiovascular events in high-risk patients. *New Engl J Med* 2000;**342**:145-53. doi:10.1056/NEJM200001203420301

43. Lonn EM, Bosch J, Lopez-Jaramillo P, et al. Blood-Pressure Lowering in Intermediate-Risk Persons without Cardiovascular Disease. *New Engl J Med* 2016;**374**:2009-20. doi:10.1056/NEJMoa1600175

44. Hansson L, Zanchetti A, Carruthers SG, et al. Effects of intensive blood-pressure lowering and low-dose aspirin in patients with hypertension: principal results of the Hypertension Optimal Treatment (HOT) randomised trial. HOT Study Group. *Lancet* 1998;**351**:1755--62.

45. Beckett NS, Peters R, Fletcher AE, et al. Treatment of hypertension in patients 80 years of age or older. *New Engl J Med* 2008;**358**:1887-98. doi:10.1056/NEJMoa0801369

46. Lewis EJ, Hunsicker LG, Clarke WR, et al. Renoprotective effect of the angiotensin-receptor antagonist irbesartan in patients with nephropathy due to type 2 diabetes. *New Engl J Med* 2001;**345**:851-60. doi:10.1056/NEJMoa011303

47. Brown MJ, Palmer CR, Castaigne A, et al. Morbidity and mortality in patients randomised to double-blind treatment with a long-acting calcium-channel blocker or diuretic in the International Nifedipine GITS study: Intervention as a Goal in Hypertension Treatment (INSIGHT). *Lancet* 2000;**356**:366-72. doi:10.1016/S0140-6736(00)02527-7

48. Pepine CJ, Handberg EM, Cooper-DeHoff RM, et al. A calcium antagonist vs a non-calcium antagonist hypertension treatment strategy for patients with coronary artery disease. The International Verapamil-Trandolapril Study (INVEST): a randomized controlled trial. *JAMA* 2003;**290**:2805-16. doi:10.1001/jama.290.21.2805

49. Cardiovascular risk and risk factors in a randomized trial of treatment based on the beta-blocker oxprenolol: the International Prospective Primary Prevention Study in Hypertension (IPPPSH). The IPPPSH Collaborative Group. *J Hypertens* 1985;**3**:379-92.

50. JATOS Study Group. Principal results of the Japanese trial to assess optimal systolic blood pressure in elderly hypertensive patients (JATOS). *Hypertens Res* 2008;**31**:2115-27. doi:10.1291/hypres.31.2115

51. Yui Y, Sumiyoshi T, Kodama K, et al. Comparison of nifedipine retard with angiotensin converting enzyme inhibitors in Japanese hypertensive patients with coronary artery disease: the Japan Multicenter Investigation for Cardiovascular Diseases-B (JMIC-B) randomized trial. *Hypertens Res* 2004;**27**:181-91.

52. Dahlof B, Devereux RB, Kjeldsen SE, et al. Cardiovascular morbidity and mortality in the Losartan Intervention For Endpoint reduction in hypertension study (LIFE): a randomised trial against atenolol. *Lancet* 2002;**359**:995-1003. doi:10.1016/S0140-6736(02)08089-3

53. The Lopressor Intervention Trial: multicentre study of metoprolol in survivors of acute myocardial infarction. Lopressor Intervention Trial Research Group. *Eur Heart J* 1987;**8**:1056-64.

54. Borhani NO, Mercuri M, Borhani PA, et al. Final outcome results of the Multicenter Isradipine Diuretic Atherosclerosis Study (MIDAS). A randomized controlled trial. *JAMA* 1996;**276**:785-91.

55. Schrader J, Luders S, Kulschewski A, et al. Morbidity and Mortality After Stroke, Eprosartan Compared with Nitrendipine for Secondary Prevention: principal results of a prospective randomized controlled study (MOSES). *Stroke* 2005;**36**:1218-26. doi:10.1161/01.STR.0000166048.35740.a9

56. MRC trial of treatment of mild hypertension: principal results. Medical Research Council Working Party. *BMJ*  1985;**291**:97-104.

57. Medical Research Council trial of treatment of hypertension in older adults: principal results. MRC Working Party. *BMJ*  1992;**304**:405-12.

58. Improvement in prognosis of myocardial infarction by long-term beta-adrenoreceptor blockade using practolol. A multicentre international study. *BMJ* 1975;**3**:735-40.

59. Navigator Study Group, McMurray JJ, Holman RR, et al. Effect of valsartan on the incidence of diabetes and cardiovascular events. *New Engl J Med* 2010;**362**:1477-90. doi:10.1056/NEJMoa1001121

60. Dens JA, Desmet WJ, Coussement P, et al. Long term effects of nisoldipine on the progression of coronary atherosclerosis and the occurrence of clinical events: the NICOLE study. *Heart* 2003;**89**:887-92.

61. Randomized double-blind comparison of a calcium antagonist and a diuretic in elderly hypertensives. National Intervention Cooperative Study in Elderly Hypertensives Study Group. *Hypertension* 1999;**34**:1129-33.

62. Hansson L, Hedner T, Lund-Johansen P, et al. Randomised trial of effects of calcium antagonists compared with diuretics and beta-blockers on cardiovascular morbidity and mortality in hypertension: the Nordic Diltiazem (NORDIL) study. *Lancet* 2000;**356**:359-65.

63. ONTARGET Investigators, Yusuf S, Teo KK, et al. Telmisartan, ramipril, or both in patients at high risk for vascular events. *New Engl J Med* 2008;**358**:1547-59. doi:10.1056/NEJMoa0801317

64. Ogawa H, Kim-Mitsuyama S, Matsui K, et al. Angiotensin II receptor blocker-based therapy in Japanese elderly, high-risk, hypertensive patients. *Am J Med* 2012;**125**:981-90. doi:10.1016/j.amjmed.2011.12.010

65. Helgeland A. Treatment of mild hypertension: a five year controlled drug trial. The Oslo study. *Am J Med* 1980;**69**:725-32.

66. MacMahon S, Sharpe N, Gamble G, et al. Randomized, placebo-controlled trial of the angiotensin-converting enzyme inhibitor, ramipril, in patients with coronary or other occlusive arterial disease. PART-2 Collaborative Research Group. Prevention of Atherosclerosis with Ramipril. *J Am Coll Cardiol* 2000;**36**:438-43.

67. Ogihara T. Practitioner's Trial on the Efficacy of Antihypertensive Treatment in the Elderly Hypertension (The PATE-Hypertension Study) in Japan. *Am J Hypertens* 2000;**13**:461-7.

68. Liu L, Wang Z, Gong L, et al. Blood pressure reduction for the secondary prevention of stroke: a Chinese trial and a systematic review of the literature. *Hypertens Research* 2009;**32**:1032-40. doi:10.1038/hr.2009.139

69. Braunwald E, Domanski MJ, Fowler SE, et al. Angiotensin-converting-enzyme inhibition in stable coronary artery disease. *New Engl J Med* 2004;**351**:2058-68. doi:10.1056/NEJMoa042739

70. Luders S, Schrader J, Berger J, et al. The PHARAO study: prevention of hypertension with the angiotensin-converting enzyme inhibitor ramipril in patients with high-normal blood pressure: a prospective, randomized, controlled prevention trial of the German Hypertension League. *J Hypertens* 2008;**26**:1487-96. doi:10.1097/HJH.0b013e3282ff8864

71. Asselbergs FW, Diercks GF, Hillege HL, et al. Effects of fosinopril and pravastatin on cardiovascular events in subjects with microalbuminuria. *Circulation* 2004;**110**:2809-16. doi:10.1161/01.CIR.0000146378.65439.7A

72. Pitt B, Byington RP, Furberg CD, et al. Effect of amlodipine on the progression of atherosclerosis and the occurrence of clinical events. PREVENT Investigators. *Circulation* 2000;**102**:1503-10.

73. Yusuf S, Diener HC, Sacco RL, et al. Telmisartan to prevent recurrent stroke and cardiovascular events. *New Engl J Med*  2008**;359**:1225-37. doi:10.1056/NEJMoa0804593

74. Randomised trial of a perindopril-based blood-pressure-lowering regimen among 6105 individuals with previous stroke or transient ischaemic attack. *Lancet* 2001;**358**:1033-41. doi:10.1016/s0140-6736(01)06178-5

75. Pitt B, O'Neill B, Feldman R, et al. The QUinapril Ischemic Event Trial (QUIET): evaluation of chronic ACE inhibitor therapy in patients with ischemic heart disease and preserved left ventricular function. *Am J Cardiol* 2001;**87**:1058-63.

76. Brenner BM, Cooper ME, de Zeeuw D, et al. Effects of losartan on renal and cardiovascular outcomes in patients with type 2 diabetes and nephropathy. *New Engl J Med* 2001;**345**:861-9. doi:10.1056/NEJMoa011161

77. Haller H, Ito S, Izzo JL, Jr., et al. Olmesartan for the delay or prevention of microalbuminuria in type 2 diabetes. *New Engl J Med* 2011;**364**:907-17. doi:10.1056/NEJMoa1007994

78. Lithell H, Hansson L, Skoog I, et al. The Study on Cognition and Prognosis in the Elderly (SCOPE): principal results of a randomized double-blind intervention trial. *J Hypertens* 2003;**21**:875-86. doi:10.1097/01.hjh.0000059028.82022.89

79. Malacco E, Mancia G, Rappelli A, et al. Treatment of isolated systolic hypertension: the SHELL study results. *Blood Pressure* 2003;**12**:160-7.

80. Prevention of stroke by antihypertensive drug treatment in older persons with isolated systolic hypertension. Final results of the Systolic Hypertension in the Elderly Program (SHEP). SHEP Cooperative Research Group. *JAMA* 1991;**265**:3255-64.

81. SPRINT Research Group, Wright JT, Jr., Williamson JD, et al. A Randomized Trial of Intensive versus Standard Blood-Pressure Control. *New Engl J Med* 2015;**373**:2103-16. doi:10.1056/NEJMoa1511939

82. S. P. S. Study Group, Benavente OR, Coffey CS, et al. Blood-pressure targets in patients with recent lacunar stroke: the SPS3 randomised trial. *Lancet* 2013;**382**:507-15. doi:10.1016/S0140-6736(13)60852-1

83. Gong L, Zhang W, Zhu Y, et al. Shanghai trial of nifedipine in the elderly (STONE). *J Hypertens* 1996;**14**:1237-45.

84. Dahlof B, Lindholm LH, Hansson L, et al. Morbidity and mortality in the Swedish Trial in Old Patients with Hypertension (STOP-Hypertension). *Lancet* 1991;**338**:1281-5.

85. Hansson L, Lindholm LH, Ekbom T, et al. Randomised trial of old and new antihypertensive drugs in elderly patients: cardiovascular mortality and morbidity the Swedish Trial in Old Patients with Hypertension-2 study. *Lancet* 1999;**354**:1751-6.

86. Sun M, Zhou H, Jia Z. [Prevention and treatment of stroke after hypertension for ten years in Hunan Province]. *Zhonghua nei ke za zhi* 1997;**36**:312-4.

87. Liu L, Wang JG, Gong L, et al. Comparison of active treatment and placebo in older Chinese patients with isolated systolic hypertension. Systolic Hypertension in China (Syst-China) Collaborative Group. *J Hypertens* 1998;**16**:1823-9.

88. Staessen JA, Fagard R, Thijs L, et al. Randomised double-blind comparison of placebo and active treatment for older patients with isolated systolic hypertension. The Systolic Hypertension in Europe (Syst-Eur) Trial Investigators. *Lancet* 1997;**350**:757-64.

89. Taylor SH, Silke B, Ebbutt A, et al. A long-term prevention study with oxprenolol in coronary heart disease. *New Engl J Med* 1982;**307**:1293-301. doi:10.1056/NEJM198211183072101

90. Eriksson S, Olofsson BO, Wester PO, et al. Atenolol in Secondary Prevention after Stroke. *Cerebrovasc Dis* 1995;**5**:21-25. doi:<https://doi.org/10.1159/000107813>

91. Neaton JD, Grimm RH, Jr., Prineas RJ, et al. Treatment of Mild Hypertension Study. Final results. Treatment of Mild Hypertension Study Research Group. *JAMA* 1993;**270**:713-24.

92. Telmisartan Randomised AssessmeNt Study in A. C. E. iNtolerant subjects with cardiovascular Disease Investigators, Yusuf S, Teo K, et al. Effects of the angiotensin-receptor blocker telmisartan on cardiovascular events in high-risk patients intolerant to angiotensin-converting enzyme inhibitors: a randomised controlled trial. *Lancet* 2008;**372**:1174-83. doi:10.1016/S0140-6736(08)61242-8

93. Julius S, Nesbitt SD, Egan BM, et al. Feasibility of treating prehypertension with an angiotensin-receptor blocker. *The New Engl J Med* 2006;**354**:1685-97. doi:10.1056/NEJMoa060838

94. Tight blood pressure control and risk of macrovascular and microvascular complications in type 2 diabetes: UKPDS 38. UK Prospective Diabetes Study Group. *BMJ* 1998;**317**:703-13.

95. Fried LF, Emanuele N, Zhang JH, et al. Combined angiotensin inhibition for the treatment of diabetic nephropathy. *The New Engl J Med* 2013;**369**:1892-903. doi:10.1056/NEJMoa1303154

96. Ogihara T, Saruta T, Rakugi H, et al. Target blood pressure for treatment of isolated systolic hypertension in the elderly: valsartan in elderly isolated systolic hypertension study. *Hypertension* 2010**;56**:196-202. doi:10.1161/HYPERTENSIONAHA.109.146035

97. Julius S, Kjeldsen SE, Weber M, et al. Outcomes in hypertensive patients at high cardiovascular risk treated with regimens based on valsartan or amlodipine: the VALUE randomised trial. *Lancet* 2004;**363**:2022-31. doi:10.1016/s0140-6736(04)16451-9

98. Rosei EA, Dal Palu C, Leonetti G, et al. Clinical results of the Verapamil in Hypertension and Atherosclerosis Study. VHAS Investigators. *J Hypertens* 1997;**15**:1337-44.

99. Wei Y, Jin Z, Shen G, et al. Effects of intensive antihypertensive treatment on Chinese hypertensive patients older than 70 years. *J Clin Hypertens* 2013;**15**:420-7. doi:10.1111/jch.12094

***Supplementary table 4.*** Variables to be sought from participating trials.

| Data | Variables |
| --- | --- |
| Study level data | Region  Treatment/Comparison groups  Study period / Duration of follow-up  Randomisation method/Treatment allocation  Outcome ascertainment  Early stopping and reasons  Funding source |
| Participant level data | Baseline characteristics  Sex, age, ethnicity, lifestyle (e.g. smoking, alcohol intake), past medical history (e.g. diabetes, cardiovascular disease, chronic renal disease), drug treatment (e.g. hypertension, dyslipidaemia, cardiovascular disease) weight, height, systolic and diastolic blood pressure, blood and urine measurements (e.g. lipids, glucose homestasis, renal function)  Follow-up variables (after randomisation)  Outcomes and relevant dates (e.g. cardiovascular events, acute renal injury / renal replacement therapy, cancer, fractures, diabetes, retinopathy, neurodegenerative conditions, serious adverse events)  Vital status (and cause of death) on last follow-up  Clinical measures and relevant dates (e.g. weight, height, systolic and diastolic blood pressure, blood and urine measurements)  Changes in treatment (e.g. discontinuation) and date |
