# Supplementary material for: Investigating the stratified efficacy and safety of pharmacological blood pressure-lowering: an overall protocol for individual patient-level data meta-analyses of over 300 000 randomised participants in the new phase of the Blood Pressure Lowering Treatment Trialists’ Collaboration (BPLTTC)
Source: BMJ Open. 2019 May 22;9(5):e028698. doi: 10.1136/bmjopen-2018-028698 (PMC6538087; doi:10.1136/bmjopen-2018-028698)
Supplement: Author's manuscript [file bmjopen-2018-028698.draft_revisions.pdf]

BMJ Open is committed to open peer review. As part of this commitment we make the peer review history of every article we publish publicly available.

When an article is published we post the peer reviewers' comments and the authors' responses online. We also post the versions of the paper that were used during peer review. These are the versions that the peer review comments apply to.

The versions of the paper that follow are the versions that were submitted during the peer review process. They are not the versions of record or the final published versions. They should not be cited or distributed as the published version of this manuscript.

BMJ Open is an open access journal and the full, final, typeset and author-corrected version of record of the manuscript is available on our site with no access controls, subscription charges or pay-per-view fees (http://bmjopen.bmj.com).

If you have any questions on BMJ Open's open peer review process please email info.bmjopen@bmj.com

BMJ Open

Investigating the stratified efficacy and safety of pharmacological blood pressure lowering: A protocol for individual patient-level data meta-analyses of over 300,000 randomized participants in the new phase of the Blood Pressure Lowering Treatment Trialists' Collaboration (BPLTTC)

Journal:	*BMJ Open*
Manuscript ID	bmjopen-2018-028698
Article Type:	Protocol
Date Submitted by the Author:	21-Dec-2018
Complete List of Authors:	Rahimi, Kazem; University of Oxford, The George Institute for Global Health, NDWRH<br>Canoy, Dexter; University of Oxford, The George Institute for Global Health, NDWRH<br>Nazarzadeh, Milad; University of Oxford, The George Institute for Global Health, NDWRH<br>Khorshidi, Gholamreza; University of Oxford, The George Institute for Global Health, NDWRH<br>Woodward, Mark; Univ Sydney<br>Teo, Koon; Population Health Research Institute<br>Davis, Barry; The University of Texas<br>Chalmers, John; The George Institute for Global Health, Professorial Unit<br>Pepine, Carl; University of Florida,
Keywords:	Blood Pressure, Meta-analyses, Efficacy and safety of BP lowering treatment

SCHOLARONE™
Manuscripts

Investigating the stratified efficacy and safety of pharmacological blood pressure lowering: A protocol for individual patient-level data meta-analyses of over 300,000 randomized participants in the new phase of the Blood Pressure Lowering Treatment Trialists' Collaboration (BPLTTC)

Kazem Rahimi,[1] Dexter Canoy,[1] Milad Nazarzadeh,[1,2] Gholamreza Khorshidi,[1] Mark Woodward,[1,3] Koon Teo,[4] Barry R Davis,[5] John Chalmers,[3] Carl J. Pepine,[6] on behalf of the Blood Pressure Lowering Treatment Trialists' Collaboration*

**Individual members listed at the end of the manuscript*

[1]The George Institute for Global Health UK, University of Oxford, Oxford, UK

[2] Collaboration Center of Meta-Analysis Research, Torbat Heydariyeh University of Medical Sciences, Torbat Heydariyeh, Iran

[3]The George Institute for Global Health, University of New South Wales, Sydney, Australia

[4]Population Health Research Institute, McMaster University, Hamilton, Ontario, Canada

[5]The University of Texas School of Public Health, Houston, Texas, USA

[6]Department of Medicine, University of Florida, Gainesville, Florida, USA

Correspondence to:

The BPLTTC

The George Institute for Global Health (UK)

University of Oxford

Hayes House, 75 George St., Oxford OX1 2BQ, United Kingdom

Tel: +441865617200; fax: +441865617202; e-mail: kazem.rahimi@georgeinstitute.ox.ac.uk

For peer review only

Abstract

Introduction: Previous research from the Blood Pressure Lowering Treatment Trialists' Collaboration (BPLTTC) and others has shown that pharmacological blood pressure (BP) lowering substantially reduces the risk of major cardiovascular events, including ischaemic heart disease, heart failure and stroke. In this new phase, the aim is to conduct individual patient-level data (IPD) meta-analyses involving eligible BP lowering randomized controlled trials (RCTs) to address uncertainties relating to efficacy and safety of BP lowering treatment.

Methods and analysis: RCTs investigating the effect of pharmacological BP lowering, with a minimum of 1000 patient-years of follow-up in each trial arm, are eligible. Our systematic review identified 100 potentially eligible trials. We requested their investigators/sponsors to contribute baseline, follow-up and outcomes data. As of January 2018, the collaboration has obtained data from 49 trials (N=315,046 participants), with additional data currently in the process of being transferred from four RCTs (N=34,642 participants). In addition, data harmonization has commenced. Scientific activities of the collaboration are overseen by the Steering Committee with input from all collaborators. Detailed protocols for individual meta-analyses will be developed and registered on public platforms.

Ethics and dissemination: Ethics approval has been obtained for this new and extended phase of the BPLTTC, the largest collaboration of de-identified IPD from RCTs. It offers an efficient and ethical manner of re-purposing existing data to answer clinically important questions relating to BP treatment as well as methodological questions relating to IPD meta-analyses. Among the immediate impacts will include reliable quantification of effects of treatment modifiers, such as baseline BP, age and prior disease, on both vascular and non-vascular outcomes. Analyses will further assess the impact of BP lowering on important, but less-well understood, outcomes, such as new-onset diabetes and renal disease. Findings will be published in peer-reviewed medical journals on behalf of the collaboration.

For peer review only - http://bmjopen.bmj.com/site/about/guidelines.xhtml

Strengths and limitations of this study

- This protocol aims to conduct individual patient-level data (IPD) of blood pressure (BP)-lowering randomised clinical trials (RCT) to address stratified efficacy of BP-lowering treatment according to various patient characteristics, examine impact of BP-lowering treatment on less-studied health vascular outcomes, and identify unintended consequences of such treatment.
- With data from 49 RCTs (>300,000 participants) already shared in the collaboration, it will be the largest IPD meta-analysis of any randomised trials, allowing sufficient statistical power to address a number of important clinical questions a single BP-lowering randomised trial is unlikely to be able to answer reliably on its own.
- Findings of meta-analyses from this resource are likely to provide reliable evidence to inform clinical guidelines and public health policy developmemt
- Data from some RCTs are not accessible because some investigators, particularly of older trials, could not be traced; or, sponsors/funders of the trials are unwilling to share data
- Not all RCTs would have collected the same clinical information so missing data is inevitable for some studies; appropriate methods to address these issues would have to be utilised

For peer review only

Introduction

Elevated blood pressure (BP) is a major risk factor for cardiovascular death and disability worldwide, and its burden has been increasing. In 2016, elevated blood pressure above the minimum risk exposure threshold of 110 mmHg systolic was responsible for more than 10 million deaths and 7% of the world's total disability-adjusted life years (DALY) [1].

Several randomized trials and their meta-analyses have shown that pharmacological BP lowering is an effective and affordable strategy for management of cardiovascular risk. Despite this, treatment and control rates among those who have been classified to have 'hypertension' (BP >140/90 mmHg) remain low in both high and low income countries [2]. This gap between evidence and practice is likely to be at least in part due to remaining uncertainties about the efficacy and safety of BP lowering [3]. For instance, there is no expert consensus on the appropriate BP threshold for treatment initiation and whether this should differ by age or disease status (e.g. diabetes or coronary heart disease [CHD]). Also, little is known on the effects of BP lowering on safety outcomes (e.g. cancer, falls and fractures, acute kidney injury), non-vascular outcomes (e.g. new-onset diabetes, dementia), or less commonly reported vascular outcomes (e.g. atrial fibrillation, peripheral arterial disease, aortic aneurysms, pulmonary embolism, stroke subtype).

These existing uncertainties have major consequences for patient management and outcomes. On the one hand, a lack of reliable estimates for the efficacy of BP lowering on less commonly reported vascular outcomes, some of which have a clear association with BP in observational studies [4–7], may result in an underestimation of the overall efficacy of BP lowering in the general patient population. On the other hand, uncertainty about the safety of BP lowering could potentially expose specific patient groups to avoidable harms because of over- or underuse of BP lowering treatment. To address these uncertainties, more evidence from randomized trials are needed. However, because single randomized trials are often not large enough for detailed investigation of the safety and efficacy of BP lowering and its variation by class of medication or important patient features, pooling information from several trials remains the most efficient and often the only viable option for addressing these remaining uncertainties.

In 1995, Principal Investigators of major ongoing clinical trials of BP lowering drugs conceived and initiated the Blood Pressure Lowering Treatment Trialists' Collaboration (BPLTTC) to investigate the effect of BP lowering on major cardiovascular events and all-cause mortality through a series of

For peer review only - http://bmjopen.bmj.com/site/about/guidelines.xhtml

individual patient data (IPD) meta-analyses [8]. Since its inception, the BPLTTC has successfully completed several studies which have provided reliable evidence for the benefits of BP lowering for prevention of major cardiovascular outcomes (CHD, heart failure and stroke) and have demonstrated that major classes of anti-hypertensives have similar beneficial effects on major vascular outcomes [9–13]. However, with its limited size and original focus on major cardiovascular outcomes only, the BPLTTC has been unable to address important contemporary questions about the stratified (i.e. subgroup-specific) effects BP lowering and its safety.

Objectives

In 2014, existing BPLTTC investigators were approached and asked to extend the scope of the collaboration with provision of information on all available and accessible baseline, follow-up and outcome data. In addition, investigators from several other large-scale trials that had not participated in BPLTTC or had provided summary data only were invited to join the collaboration with provision of IPD. The key objectives for this extended phase of BPLTTC were specified as follows:

1. To assess the stratified efficacy of BP lowering on major vascular events (CHD, stroke and heart failure) and all-cause mortality by baseline BP levels, sex, age, disease status (including multimorbidity), and risk of CVD at baseline.
2. To assess the overall and stratified safety of BP lowering examining its effect on unintended outcomes, including total serious adverse events, acute renal failure, new-onset diabetes, fractures, and cancer overall and by drug classes and baseline BP levels.
3. To assess the extended efficacy of BP lowering on other important 'vascular' outcomes, including atrial fibrillation and dementia.

Methods and analysis

Inclusion and exclusion criteria:

For this extended phase of the collaboration, the original trial eligibility criteria were modified, chiefly to enable inclusion of additional large-scale trials that were reported before the inception of the BPLTTC in 1995. We will only include randomized clinical trials to minimise the impact of bias and confounding. For clinical trials to be eligible for inclusion in BPLTTC, one of the following criteria must be met:

For peer review only - http://bmjopen.bmj.com/site/about/guidelines.xhtml

- Randomization of patients between a BP lowering agent and a placebo arm (or other inactive control condition)
- Randomization of patients between various BP lowering intensities
- Randomization of patients between various antihypertensive drugs

Additionally, a minimum of 1000 patient-years of follow-up in each randomly allocated trial arm is required. There is no restriction on publication date, setting, or drugs investigated. However, the following types of trial will be excluded:

- Trials exclusively conducted in patients with heart failure or short-term interventions in patients with acute myocardial infarction or other acute settings
- Trials with non-pharmacological interventions of BP lowering without a drug comparison arm
- Trials without a clearly defined randomization process

Identification of trials

We conducted a systematic review to identify trials for possible inclusion in the IPD meta-analysis. The search was restricted to clinical trials, controlled clinical trials, randomized controlled trials or meta-analyses and no language restrictions were applied. We searched electronic bibliographic databases, including PubMed/Medline (NCBI, Bethesda, MD, USA) and The Cochrane Central Register of Controlled Trials (The Cochrane Collaboration, London, UK) covering the periods between 1 Jan 1966 and 1 Jun 2018. Reference lists of eligible studies, related meta-analyses, clinical trial registries, and BPLTTC collected in previous projects [14] were hand-searched to identify further relevant studies. This systematic review protocol, including details of the methods and search strategy, was registered with PROSPERO (www.crd.york.ac.uk/prospero/) (*CRD42018099283*) before the review was conducted. ***Supplementary table 1*** shows the Medline searh strategy. For this protocol, the Preferred Reporting Items for Systematic review and Meta-Analysis Protocols (PRISMA-P) checklist is shown in ***Supplementary Table 2***.

We used reference management software (Endnote) to store identified records from different sources. Duplicate records from various sources were collapsed into a single entry. Two investigators (DC and MN) conducted independent searches and screened the title and abstracts of publications for meeting eligibility criteria. The full manuscript were then requested and assessed

For peer review only - http://bmjopen.bmj.com/site/about/guidelines.xhtml

for eligibility. Disagreements were resolved by discussion, with involvement of a third investigator (KR) where necessary.

Because the purpose of this systematic review was to identify potential trials for inclusion in an IPD meta-analysis, we mainly extracted information about the investigators and/or contact details to allow us to invite them to participate by contributing data. When conducting the meta-analysis of RCTs for which we have obtained data at individual patient-level, we will report quality assessment of these RCTs based on randomization procedures, treatment allocations according to assignment, and outcomes collected and compared across comparison groups; concealment and blinding methods; and risk of bias both at study and outcome levels using the Cochrane Risk of Bias Tool. We will use the quality of RCTs as part of sensitivity analysis when conducting the IPD meta-analysis.

Figure 1 summarizes the trial search as of June 2018, indicating that a total of 100 trials with 595,514 randomized participants were identified that potentially met the BPLTTC eligibility criteria (***Supplementary Table 3***). This literature search will be continuously updated, and investigators of newly reported and eligible trials will be invited to join the collaboration.

Data collection

All existing BPLTT collaborators and investigators of all newly identified trials were contacted and asked whether they would be willing to provide IPD (variables are listed in ***Supplementary table 4***). As of June 2018, the collaboration had acquired data from 49 trials comprising 315,046 participants. Investigators or sponsors of several other trials have agreed to share data. Data from another four major trials with 34,642 participants are currently at the transfer stage. For the remaining trials, data are still being sought. Many of these trials have been published many years ago and identification of the data guardian or an electronic database of the trial has proven challenging.

In parallel to requesting further data, the process of data harmonization has commenced. This includes creation of database with all information obtained from investigators, using the data dictionary provided. Initial data verification will include checking of the individual patient data with respect to number of participants, baseline characteristics, and types of follow-up and outcome data available. Next, prepared data are to be checked for consistency (e.g. plausible ranges, randomization sequence etc.) and completeness. In case of any inconsistencies or missing information, the investigators will be contacted for verification.

For peer review only - http://bmjopen.bmj.com/site/about/guidelines.xhtml
For peer review only

Governance and data management policies

Figure 2 provides an overview of roles and responsibilities of the BPLTTC collaborative group. The BPLTTC Director, supported by a core analytic and management group, at the University of Oxford coordinates all activities among internal collaborators and external stakeholders. This central team will periodically update the literature review, acquire new data, develop proposals and protocols, seek approvals and funding, and manage publications and communication. Scientific activities of the BPLTTC core group are overseen by the Steering Committee with input from all BPLTT collaborators.

The aforementioned three main objectives of the extended phase of BPLTTC have all been approved by the Steering Committee and collaborators. *For each of these objectives, at least one study protocol will be developed*. Collaborators or external researchers may suggest additional research proposals to the BPLTTC core group. Such proposals shall be checked for feasibility and overlap with any existing proposals. If appropriate, they will be shared with the Steering Committee for scientific review. After approval, proposing researchers will be able to join a working group, including other interested members of the Collaboration. This working group will work with the BPLTTC core group and Steering Committee to write the research protocol, conduct and interpret the results, draft the manuscript and publish the final report.

Once agreement on a structured protocol is reached by the working group, the study will be registered with PROSPERO and the protocol will be posted on a password protected website at www.bplttc.org. Additionally, collaborators will be able to use this website to stay informed about the progress of various projects.

Trial data from investigators and sponsors are typically transferred by using a secure file transmission system, such as Oxford's Oxfile system. Before sending a data file, the data will be compressed using a standard compression system and then encrypted. All data transfer processes will be overseen by the BPLTTC core group in compliance with the data exchange and sharing agreement with the data provider. All trial data will be considered confidential and will not be provided to any third party without written permission of the owner of the data. Data will be stored on site at the George Institute at the University of Oxford, with strict confidentiality and

For peer review only

comprehensive data security. All analyses will be carried out by the core analytic team, according to research priorities and availability of resources.

Patient and public involvement

This protocol describes plans for collaborative research investigations involving secondary analyses of existing clinical trials data. There was neither any involvement by patients in conceptualising the study nor in the development of the protocol.

Statistical analysis

Prior to commencing any analysis, a feasibility assessment and power calculation will be carried out on the blinded dataset. For approved proposals, a protocol will be written which will entail a detailed statistical analysis plan. Although specific proposals may require different statistical methods, in principle, we intend to use a one-step approach by simultaneously analysing observations from all trials (while accounting for clustering of observations within studies) [15][16]. This approach is more suitable than a two-step approach when non-linear effects or multiple covariates and their interaction are to be assessed, which our stratified analyses aim to do [17]. For some analyses, it might be important to supplement the available IPD with aggregate data for those studies where IPD are not available, using a combined one-step and two-step approaches. Aggregate data will be obtained by sending our computer codes to the Investigators of such studies, so as to extract their results (e.g. hazard ratios), rather than their data.

We will use fixed effects methods to pool trial-specific data. For time-to-event data clustered Cox proportional-hazard models will be used and hazard ratios with 95% or 99% confidence intervals (CI) will be reported. For binary outcomes, risk ratios and 95% CI will be estimated using log-binomial mixed-effects models (or odds ratios from logistic models, if necessary). Effects on continuous outcomes (e.g. renal function) will be quantified by mean differences and 95% CI and will be estimated using linear mixed effects models.

Using a two-step approach, key results will be presented using Forest plots, and Cochran's Q test and I^2 statistic will be used to quantify the degree of heterogeneity between studies. Meta-regression will be used if considerable residual heterogeneity remains after controlling for all possible variables and recognized effect modifiers, with P-value adjusted for false positive findings based on Monte Carlo simulation.

For peer review only - http://bmjopen.bmj.com/site/about/guidelines.xhtml

Where appropriate, analyses will also be stratified by class of medication, patient population and baseline BP to allow for heterogeneity among different patient populations and types of medication to be determined. In addition, a dose-effect (dose-response) regression model will be applied to assess any potential dose-effect association for each medication dosage and represent efficacy and probable risk of side effects (point-to-point association) both at individual and trial level (aggregate data). Also, we will use fractional polynomials and cubic splines to smoothen the dose-response relationship and address any violated linearity assumption [18].

Most trials included in BPLTTC have a double blind design where the use of routine unadjudicated outcomes is unlikely to result in biased estimates (provided sufficient number of events can be gathered) because any under- or over-reporting would affect both arms of the trial equally [19,20]. However, due to the differing data formats of contributing trials and potentially differing types of data provided, additional methodological work might be needed to enhance the quality and power of the data. This might be particularly relevant when a large proportion of data on important covariates, effect modifiers or outcomes is missing. Simply ignoring the missing data, or implementing a complete case analysis, is typically inappropriate, as it could result in biased results. We will therefore conduct methodological studies that will compare existing and novel methods for imputation of non-commensurate measurements between trials, correction of measurement errors, or harmonization of differing definitions among trials [21–24].

In general, reporting of the findings will follow the PRISMA-IPD guidance [25] and where appropriate, we will use Grading of Recommendations Assessment, Development and Evaluation (GRADE) (www.gradeworkinggroup.org) when assessing certainty of evidence.

Ethics and dissemination

As we do not have access to patient identifiable information, our procedures are fully compliant with the Data Protection Act 1998. The University of Oxford's research contracts team is well aware of legal requirements for data sharing and storage and all our procedures are fully compliant with these requirements. We have already obtained ethics review from the Oxford Central Ethics University Research Committee (OxTREC Reference: 545-14) and agreed to communicate any major amendments to the protocol for this third cycle of BPLTTC with the Ethics Committee for further approval before analyses begin.

For peer review only - http://bmjopen.bmj.com/site/about/guidelines.xhtml

The knowledge of the degree of efficacy and safety of BP lowering and its variation by class of medication and among subgroups of patients will help to refine treatment recommendations. The presence of clinically meaningful differences in the efficacy of different classes of BP medication, either overall, or within certain patient groups will have considerable implications for stratified patient management and clinical decision making. BPLTTC is the largest international hypertension collaboration and is unique in its ability to pool and analyse IPD from a large number of trials to answer these clinically relevant questions.

Authors' contributions

KR wrote the first draft of the manuscript; KR and DC sought access to the data; DC, ML and KR conducted the systematic review; all authors contributed in the conception and design of the study, provided critical input into the subsequent revisions of the manuscript, and gave approval to the final version to be published; KR and DC are guarantors of this protocol.

Funding statement

BPLTTC is supported by the Oxford Martin School and the National Institute for Health Research (NIHR) Oxford Biomedical Research Centre (BRC).

The views expressed are those of the author(s) and not necessarily those of the NHS, the NIHR or the Department of Health; Funders have no involvement in the conduct of the review or in the development of the protocol.

Competing interests statement

George Health Enterprises, the social enterprise arm of The George Institute, has received investment to develop fixed dose combinations of aspirin, statin and blood pressure lowering drugs.

For peer review only

The Blood Pressure Lowering Treatment Trialists' Collaboration

Steering Committee

Kazem Rahimi (Chair) (The George Institute for Global Health, University of Oxford, Oxford, United Kingdom), Koon Teo (Population Health Research Institute, McMaster University, Hamilton, Ontario, Canada) Barry R Davis (The University of Texas School of Public Health, Houston, Texas, USA), John Chalmers (The George Institute for Global Health, University of New South Wales, Sydney, Australia), Carl J. Pepine (Department of Medicine, University of Florida, Gainesville, Florida, USA)

Collaborating Trialists

L Agodoa (AASK [African-American Study of Kidney Disease and Hypertension]), A Algra (Dutch TIA Study [Dutch Transient Ischemic Attack Study]), F W Asselbergs (PREVEND-IT [Prevention of Renal and Vascular End- stage Disease Intervention Trial], Renal analyses), N Beckett (HYVET [Hypertension in the Very Elderly Trial]), E Berge (VALUE trial [Valsartan Antihypertensive Long-term Use Evaluation trial]), H Black (CONVINCE [Controlled Onset Verapamil Investigation of Cardiovascular End Points]), B Brenner (RENAAL [Reduction of Endpoints in NIDDM with the Angiotensin II Antagonist Losartan]), F.P.J. Brouwers (PREVEND-IT), M Brown (INSIGHT [International Nifedipine GITS Study: Intervention as a Goal in Hypertension]), C J Bulpitt (HYVET), B Byington (PREVENT [Prospective Randomized Evaluation of the Vascular Effects of Norvasc Trial]), J Chalmers (ADVANCE[Action in Diabetes and Vascular Disease: Preterax and Diamicron MR Controlled Evaluation]), J Cutler (ALLHAT [Antihypertensive and Lipid-Lowering Treatment to Prevent Heart Attack Trial]), B Davis (ALLHAT), R B Devereaux (LIFE [Losartan Intervention For Endpoint reduction in hypertension]), D Dwyer (IDNT [Irbesartan Diabetic Nephropathy Trial]), R Estacio (ABCD [Appropriate Blood Pressure Control in Diabetes]), R Fagard (SYST-EUR [SYSTtolic Hypertension in EURope]), K Fox (EUROPA [European trial on Reduction Of cardiac events with Perindopril among patients with stable coronary Artery disease]), T Fukui (CASE-J [Candesartan Antihypertensive Survival Evaluation in Japan]), A J Gupta (ASCOT [Anglo-Scandinavian Cardiac Outcomes Trial]), Y Imai (HOMED-BP [Hypertension Objective Treatment Based on Measurement by Electrical Devices of Blood Pressure]), R R Holman (UKPDS [UK Prospective Diabetes Study]), M Ishii (JATOS [Japanese Trial to Assess Optimal Systolic Blood Pressure in Elderly Hypertensive Patients], JMIC-B [Japan Multicenter Investigation for Cardiovascular Diseases-B]), S Julius (VALUE), Y Kanno (ECOST [The Effectiveness and Cost of ICD follow-up Schedule with Telecardiology]), S E Kjeldsen (VALUE, LIFE), J Kostis (SHEP [Systolic Hypertension in the Elderly Program]) K Kuramoto (NICS-EH [National Intervention Cooperative Study in Elderly Hypertensives]), J Lanke (STOP2 [Swedish Trial in Old Patients with Hypertension-2], NORDIL [Nordic Diltiazem]), E Lewis (IDNT), J Lewis (IDNT) M Lievre (DIABHYCAR [Non-insulin-dependent diabetes, hypertension, microalbuminuria or proteinuria, cardiovascular events, and ramipril study]), L H Lindholm (CAPPP [Captopril Prevention Project], STOP2, NORDIL), L Lisheng (FEVER [Felodipine Event Reduction]), J Lubsen (ACTION [A Coronary disease Trial Investigating Outcome with Nifedipine GITS]), S Lueders (MOSES [The Morbidity and Mortality After Stroke, Eprosartan Compared With Nitrendipine for Secondary Prevention]), E Malacco (SHELL [Systolic Hypertension in the Elderly: Lacidipine Long-Term study]), G Mancia (SHELL), S MacMahon (ADVANCE), M Matsuzaki (COPE [The Combination Therapy of Hypertension to Prevent Cardiovascular Events]), M H Mehlum (VALUE), S Nissen (CAMELOT [Comparison of Amlodipine vs Enalapril to Limit Occurrences of Thrombosis]), H Ogawa (HIJ-CREATE), T Othisgihara (CASE-J), T Ohkubo (HOMED-BP), C Palmer (INSIGHT), A Patel (ADVANCE), C Pepine (INVEST [International Verapamil SR-Trandolapril Study]), M Pfeffer (PEACE [Prevention of Events With Angiotensin- Converting Enzyme Inhibition]), B Pitt (QUIET [QUinapril Ischemic Event Trial]), N R Poulter (ASCOT [Anglo-

For peer review only - http://bmjopen.bmj.com/site/about/guidelines.xhtml

Scandinavian Cardiac Outcomes Trial]), H Rakugi (VALISH/CASE-J), G Remuzzi (BENEDICT [BErgamo NEphrologic DIabetes Complications Trial]), P Ruggenenti (BENEDICT), T Saruta (CASE-J), J Schrader (MOSES), R Schrier (ABCD), P Sever (ASCOT), P Sleight (CONVINCE, HOPE [Heart Outcomes Prevention Evaluation], TRANSCEND [Telmisartan Randomised AssessmeNt Study in ACE iNtolerant subjects with cardiovascular Disease], ONTARGET [Ongoing Telmisartan Alone and in Combination with Ramipril Global Endpoint Trial]), J A Staessen (SYST-EUR [Systolic Hypertension in Europe]), H Suzuki (ECOST [Efficacy of Candesartan on Outcome in Saitama Trial]), K Teo (SCAT [Simvastatin/Enalapril Coronary Atherosclerosis Trial]), L Thijs (Syst-Eur), K Ueshima (VALISH/CASE-J), S Umemoto (COPE), W H van Gilst (PREVEND-IT/Renal analyses), P Verdeccia (Cardio-Sis), G Viberti (ROADMAP [Randomised Olmesartan and Diabetes Microalubuminuria Prevention]), K Wachtell (LIFE), J Wang (NICOLE [NIsoldipine in COronary artery disease in LEuven]), L Wing (ANBP2 [The Second Australian National Blood Pressure Study]), Y Yui (JMIC-B), S Yusuf (HOPE, ONTARGET, TRANSCEND), A Zanchetti (deceased) (SCOPE [Study on COgnition and Prognosis in the Elderly], VHAS [Verapamil in Hypertension and Atherosclerosis Study], ELSA [European Lacidipine Study on Atherosclerosis], HOT [Hypertension Optimal Treatment]).

Other members

C Baigent, R Collins, D de Zeeuw B Neal, V Perkovic, M Rahman, W J Remme, A Rodgers, J Sundström, F Turnbull.

For peer review only

For peer review only - http://bmjopen.bmj.com/site/about/guidelines.xhtml

Figure legends

Figure 1: Literature search and trial selection flowchart.

Figure 2: Overview of collaboration governance.

For peer review only

References

1 GBD 2016 Risk Factors Collaboration, Abajobir AA, Abate KH, *et al.* Global, regional, and national comparative risk assessment of 84 behavioural, environmental and occupational, and metabolic risks or clusters of risks, 1990–2016: a systematic analysis for the Global Burden of Disease Study 2016. *Lancet* 2017;**390**:1345–422. doi:10.1016/S0140-6736(17)32366-8

2 Mills KT, Bundy JD, Kelly TN, *et al.* Global Disparities of Hypertension Prevalence and Control Clinical Perspective: a Systematic Analysis of Population-Based Studies from 90 Countries. *Circulation* 2016;**134**:441–50. doi:10.1161/CIRCULATIONAHA.115.018912

3 Rahimi K, Emdin CA, MacMahon S. The epidemiology of blood pressure and its worldwide management. *Circ Res* 2015;**116**:925–36. doi:10.1161/CIRCRESAHA.116.304723

4 Emdin CA, Anderson SG, Salimi-Khorshidi G, *et al.* Usual blood pressure, atrial fibrillation and vascular risk: Evidence from 4.3 million adults. *Int J Epidemiol* 2017;**46**:162–72. doi:10.1093/ije/dyw053

5 Emdin CA, Rothwell PM, Salimi-Khorshidi G, *et al.* Blood Pressure and Risk of Vascular Dementia. *Stroke* 2016;**47**:1429–35. doi:10.1161/STROKEAHA.116.012658

6 Emdin CA, Anderson SG, Callender T, *et al.* Usual blood pressure, peripheral arterial disease, and vascular risk: cohort study of 4.2 million adults. *BMJ* 2015;**351**:h4865.

7 Emdin CA, Anderson SG, Woodward M, *et al.* Usual Blood Pressure and Risk of New-Onset Diabetes: Evidence From 4.1 Million Adults and a Meta-Analysis of Prospective Studies. *J Am Coll Cardiol* 2015;**66**:1552–62. doi:10.1016/j.jacc.2015.07.059

8 Protocol for prospective collaborative overviews of major randomized trials of blood-pressure-lowering treatments. World Health Organization-International Society of Hypertension Blood Pressure Lowering Treatment Trialists' Collaboration. *J Hypertens* 1998;**16**:127–37.

9 Blood Pressure Lowering Treatment Trialists' Collaboration, Turnbull F, Neal B, *et al.* Effects of different regimens to lower blood pressure on major cardiovascular events in older and younger adults: meta-analysis of randomised trials. *BMJ* 2008;**336**:1121–3. doi:10.1136/bmj.39548.738368.BE

10 Turnbull F, Blood Pressure Lowering Treatment Trialists' Collaboration. Effects of different blood-pressure-lowering regimens on major cardiovascular events: results of prospectively-designed overviews of randomised trials. *Lancet (London, England)* 2003;**362**:1527–35.

11 Sundström J, Arima H, Jackson R, *et al.* Effects of blood pressure reduction in mild hypertension: a systematic review and meta-analysis. *Ann Intern Med* 2015;**162**:184–91. doi:10.7326/M14-0773

12 Blood Pressure Lowering Treatment Trialists' Collaboration, Ying A, Arima H, *et al.* Effects of blood pressure lowering on cardiovascular risk according to baseline body-mass index: a meta-analysis of randomised trials. *Lancet (London, England)* 2015;**385**:867–74. doi:10.1016/S0140-6736(14)61171-5

13 Turnbull F, Woodward M, Neal B, *et al.* Do men and women respond differently to blood pressure-lowering treatment? Results of prospectively designed overviews of randomized trials. *Eur Heart J* 2008;**29**:2669–80. doi:10.1093/eurheartj/ehn427

14 Ettehad D, Emdin CA, Kiran A, *et al.* Blood pressure lowering for prevention of cardiovascular disease and death: a systematic review and meta-analysis. *Lancet* 2016;**387**:957–67. doi:10.1016/S0140-6736(15)01225-8

15 Hamza TH, van Houwelingen HC, Stijnen T. The binomial distribution of meta-analysis was preferred to model within-study variability. *J Clin Epidemiol* 2008;**61**:41–51. doi:10.1016/j.jclinepi.2007.03.016

For peer review only

For peer review only - http://bmjopen.bmj.com/site/about/guidelines.xhtml

16 Stijnen T, Hamza TH, Özdemir P. Random effects meta-analysis of event outcome in the framework of the generalized linear mixed model with applications in sparse data. *Stat Med* 2010;**29**:3046–67. doi:10.1002/sim.4040
17 Abo-Zaid G, Guo B, Deeks JJ, *et al.* Individual participant data meta-analyses should not ignore clustering. *J Clin Epidemiol* 2013;**66**:865–873.e4. doi:10.1016/j.jclinepi.2012.12.017
18 Bagnardi V, Zambon A, Quatto P, *et al.* Flexible Meta-Regression Functions for Modeling Aggregate Dose-Response Data, with an Application to Alcohol and Mortality. *Am J Epidemiol* 2004;**159**:1077–86. doi:10.1093/aje/kwh142
19 Rahimi K, Bhala N, Kamphuisen P, *et al.* Effect of Statins on Venous Thromboembolic Events: A Meta-analysis of Published and Unpublished Evidence from Randomised Controlled Trials. *PLoS Med* 2012;**9**:e1001310. doi:10.1371/journal.pmed.1001310
20 Pogue J, Walter SD, Yusuf S. Evaluating the benefit of event adjudication of cardiovascular outcomes in large simple RCTs. *Clin Trials* 2009;**6**:239–51. doi:10.1177/1740774509105223
21 Resche-Rigon M, White IR, Bartlett JW, *et al.* Multiple imputation for handling systematically missing confounders in meta-analysis of individual participant data. *Stat Med* 2013;**32**:4890–905. doi:10.1002/sim.5894
22 Siddique J, Reiter JP, Brincks A, *et al.* Multiple imputation for harmonizing longitudinal non-commensurate measures in individual participant data meta-analysis. *Stat Med* 2015;**34**:3399–414. doi:10.1002/sim.6562
23 Kunkel D, Kaizar EE. A comparison of existing methods for multiple imputation in individual participant data meta-analysis. *Stat Med* 2017;**36**:3507–32. doi:10.1002/sim.7388
24 Schifeling T, Reiter JP, DeYoreo M. Data Fusion for Correcting Measurement Errors. 2016;:1–31.
25 Stewart LA, Clarke M, Rovers M, *et al.* Preferred Reporting Items for a Systematic Review and Meta-analysis of Individual Participant Data. *JAMA* 2015;**313**:1657. doi:10.1001/jama.2015.3656

For peer review only

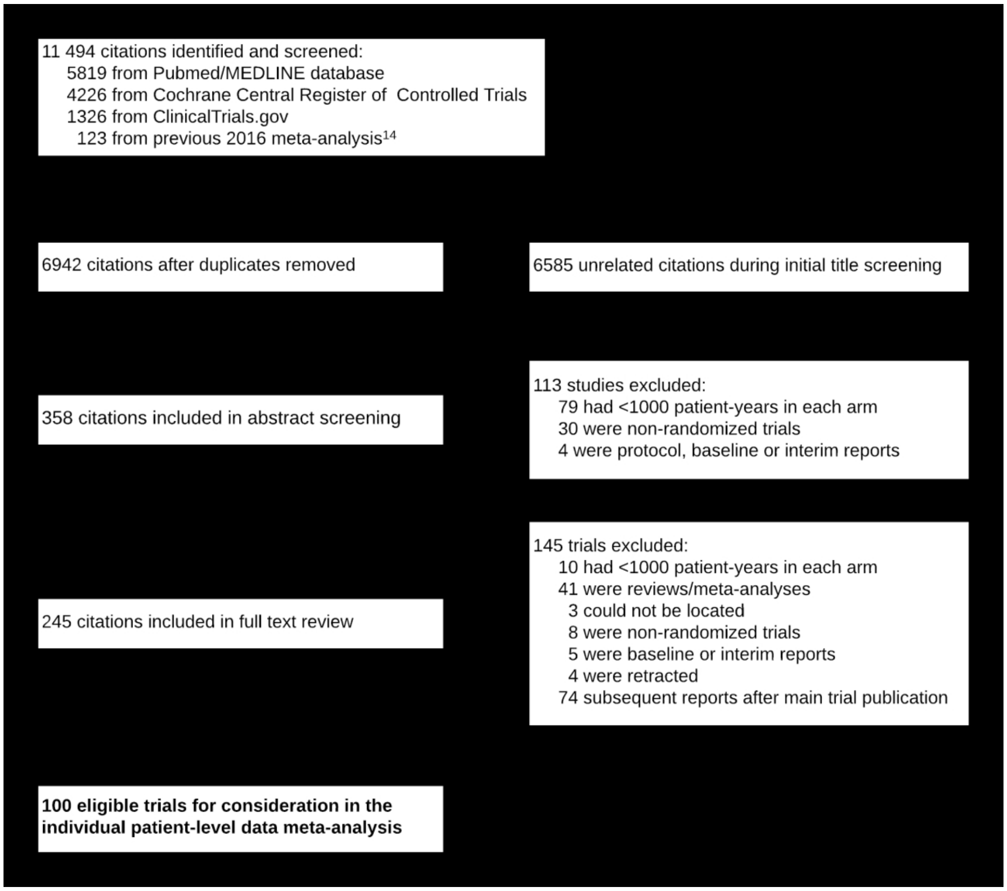

Figure 1

780x687mm (72 x 72 DPI)

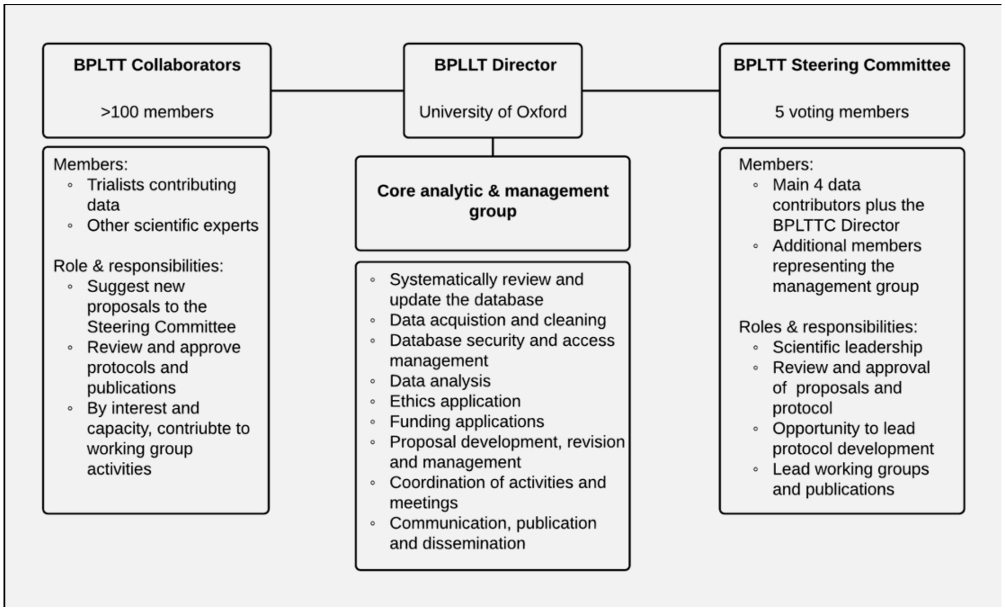

187x113mm (150 x 150 DPI)

SUPPLEMENTARY MATERIAL

Investigating the stratified efficacy and safety of pharmacological blood pressure lowering: A protocol for individual patient-level data meta-analyses of over 300,000 randomized participants in the new phase of the Blood Pressure Lowering Treatment Trialists' Collaboration (BPLTTC)

Kazem Rahimi,[1] Dexter Canoy,[1] Milad Nazarzadeh,[1,2] Gholamreza Khorshidi,[1] Mark Woodward,[1,3] Koon Teo,[4] Barry R Davis,[5] John Chalmers,[3] Carl J. Pepine,[6] on behalf of the Blood Pressure Lowering Treatment Trialists' Collaboration

[1]The George Institute for Global Health UK, University of Oxford, Oxford, UK

[2] Collaboration Center of Meta-Analysis Research, Torbat Heydariyeh University of Medical Sciences, Torbat Heydariyeh, Iran

[3]The George Institute for Global Health, University of New South Wales, Sydney, Australia

[4]Population Health Research Institute, McMaster University, Hamilton, Ontario, Canada

[5]The University of Texas School of Public Health, Houston, Texas, USA

[6]Department of Medicine, University of Florida, Gainesville, Florida, USA

E-mail address:

kazem.rahimi@georgeinstitute.ox.ac.uk (KR)

dexter.canoy@georgeinstitute.ox.ac.uk (DC)

milad.nazarzadehlarzjan@georgeinstitute.ox.ac.uk (ML)

reza.khorshidi@georgeinstitute.ox.ac.uk (GK)

mark.woodward@georgeinstitute.ox.ac.uk (MW)

Koon.Teo@phri.ca (KT)

Barry.R.Davis@uth.tmc.edu (BRD)

chalmers@georgeinstitute.org.au (JC)

Carl.Pepine@medicine.ufl.edu (CP)

For peer review only

Supplementary table 1. Search strategy to be used in MEDLINE, covering indexed entries between 01 Jan 1966 to 01 Jun 2018.

Search (((((("Hypertension/drug effects"[Mesh] OR "Hypertension/drug therapy"[Mesh]))) AND (("Blood Pressure/drug effects"[Mesh] OR "Blood Pressure/therapy"[Mesh]))) AND ((Clinical Trial[ptyp] OR Controlled Clinical Trial[ptyp] OR Meta-Analysis[ptyp] OR Randomized Controlled Trial[ptyp] OR Clinical Trial, Phase III[ptyp]) AND Humans[Mesh] AND adult[MeSH]))) AND (((((((((("Antihypertensive Agents" [Pharmacological Action]) OR "Antihypertensive Agents/therapeutic use"[Mesh])) OR ((("Vasodilator Agents" [Pharmacological Action])) OR ("Vasodilator Agents/therapeutic use"[Mesh] OR "Vasodilator Agents/therapy"[Mesh]))) OR (("Adrenergic alpha-Antagonists/therapeutic use"[Mesh]) OR "Adrenergic alpha-Antagonists" [Pharmacological Action])) OR (("Adrenergic beta-Antagonists" [Pharmacological Action]) OR "Adrenergic beta-Antagonists/therapeutic use"[Mesh])) OR (("Sodium Chloride Symporter Inhibitors" [Pharmacological Action]) OR "Sodium Chloride Symporter Inhibitors/therapeutic use"[Mesh])) OR "Angiotensin-Converting Enzyme Inhibitors/therapeutic use"[Mesh]) OR (("Angiotensin II Type 1 Receptor Blockers" [Pharmacological Action]) OR "Angiotensin II Type 1 Receptor Blockers/therapeutic use"[Mesh])) OR (("Calcium Channel Blockers" [Pharmacological Action]) OR "Calcium Channel Blockers/therapeutic use"[Mesh])) AND ((Clinical Trial[ptyp] OR Controlled Clinical Trial[ptyp] OR Meta-Analysis[ptyp] OR Randomized Controlled Trial[ptyp] OR Clinical Trial, Phase III[ptyp]) AND Humans[Mesh] AND adult[MeSH])) Filters: Clinical Trial; Controlled Clinical Trial; Randomized Controlled Trial; Clinical Trial, Phase III; Meta-Analysis; Systematic Reviews; Humans; Adult: 19+ years

For peer review only

Supplementary table 2. PRISMA-P (Preferred Reporting Items for Systematic review and Meta-Analysis Protocols) checklist for the Blood Pressure Lowering Treatment Trialists' Collaboration protocol for individual patient-level data meta-analysis

Section and topic	Item No	Checklist item	Location in the manuscript
**ADMINISTRATIVE INFORMATION**			
Title:			
Identification	1a	Identify the report as a protocol of a systematic review	Title page
Update	1b	If the protocol is for an update of a previous systematic review, identify as such	NA
Registration	2	If registered, provide the name of the registry (such as PROSPERO) and registration number	Page 5
Authors:			
Contact	3a	Provide name, institutional affiliation, e-mail address of all protocol authors; provide physical mailing address of corresponding author	Title page (email address in Supplementary material title page)
Contributions	3b	Describe contributions of protocol authors and identify the guarantor of the review	Page 10
Amendments	4	If the protocol represents an amendment of a previously completed or published protocol, identify as such and list changes; otherwise, state plan for documenting important protocol amendments	NA
Support:			
Sources	5a	Indicate sources of financial or other support for the review	Page 10
Sponsor	5b	Provide name for the review funder and/or sponsor	Page 10
Role of sponsor or funder	5c	Describe roles of funder(s), sponsor(s), and/or institution(s), if any, in developing the protocol	Page 10
**INTRODUCTION**			
Rationale	6	Describe the rationale for the review in the context of what is already known	Page 3
Objectives	7	Provide an explicit statement of the question(s) the review will address with reference to participants, interventions, comparators, and outcomes (PICO)	Page 4
**METHODS**			
Eligibility criteria	8	Specify the study characteristics (such as PICO, study design, setting, time frame) and report characteristics (such as years considered, language, publication status) to be used as criteria for eligibility for the review	Pages 4 to 5
Information sources	9	Describe all intended information sources (such as electronic databases, contact with study authors, trial registers or other grey literature sources) with planned dates of coverage	Page 5

For peer review only - http://bmjopen.bmj.com/site/about/guidelines.xhtml

Search strategy	10	Present draft of search strategy to be used for at least one electronic database, including planned limits, such that it could be repeated	Supplementary table 1
Study records:			
Data management	11a	Describe the mechanism(s) that will be used to manage records and data throughout the review	Page 5
Selection process	11b	State the process that will be used for selecting studies (such as two independent reviewers) through each phase of the review (that is, screening, eligibility and inclusion in meta-analysis)	Pages 5 to 6
Data collection process	11c	Describe planned method of extracting data from reports (such as piloting forms, done independently, in duplicate), any processes for obtaining and confirming data from investigators	Page 6
Data items	12	List and define all variables for which data will be sought (such as PICO items, funding sources), any pre-planned data assumptions and simplifications	Supplementary table 3 (Protocols for each meta-analysis will be developed and published which will include specific definitions of relevant variables)
Outcomes and prioritization	13	List and define all outcomes for which data will be sought, including prioritization of main and additional outcomes, with rationale	For each meta-analysis (to address each specific objectives), specific protocol will be developed and published which will include a definition of the outcome of interest
Risk of bias in individual studies	14	Describe anticipated methods for assessing risk of bias of individual studies, including whether this will be done at the outcome or study level, or both; state how this information will be used in data synthesis	Page 6
Data synthesis	15a	Describe criteria under which study data will be quantitatively synthesised	Pages 8 to 9
	15b	If data are appropriate for quantitative synthesis, describe planned summary measures, methods of handling data and methods of combining data from studies, including any planned exploration of consistency (such as I^2, Kendall's τ)	Pages 8 to 9 (more detailed analytical plans will be described in separate protocols for each meta-analysis)
	15c	Describe any proposed additional analyses (such as sensitivity or subgroup analyses, meta-regression)	Pages 8 to 9
	15d	If quantitative synthesis is not appropriate, describe the type of summary planned	NA
Meta-bias(es)	16	Specify any planned assessment of meta-bias(es) (such as publication bias across studies, selective reporting within studies)	Pages 6 and 9 (specific assessments will be described in the protocol for each planned meta-analysis)
Confidence in cumulative evidence	17	Describe how the strength of the body of evidence will be assessed (such as GRADE)	Page 9

Reference: Shamseer L, Moher D, Clarke M, Ghersi D, Liberati A, Petticrew M, Shekelle P, Stewart L, PRISMA-P Group. Preferred reporting items for systematic review and meta-analysis protocols (PRISMA-P) 2015: elaboration and explanation. BMJ. 2015;349:g7647.

For peer review only - http://bmjopen.bmj.com/site/about/guidelines.xhtml
For peer review only

Supplementary table 3. Blood pressure lowering randomized trials eligible for BPLTTC individual patient-level data meta-analysis.

Study name or author	Publication year	Total N	Intervention/Comparisons
**AASK** (African American Study of Kidney Disease and Hypertension)[1]	2006	1094	Intensive BP lowering, moderate BP lowering, ACEi, CCB and BB (factorial design)
**ABCD** (Appropriate Blood Pressure Control in Diabetes Trial)[2]	1998	1900	BP lowering to different targets
**ACCOMPLISH** (Avoiding Cardiovascular events through Combination therapy in Patients Living with Systolic Hypertension)[3]	2008	11,506	CCB + ACEi vs diuretic
**ACCORD** (Action to Control Cardiovascular Risk in Diabetes)[4]	2010	4733	Intensive vs standard BP lowering
**ACTION** (A Coronary Disease Trial Investigating Outcome with Nifedipine GITS)[5]	2004	7665	CCB vs placebo
**ACTIVE I** (Atrial Fibrillation Clopidogrel Trial with Irbesartan for Prevention of Vascular Events)[6]	2011	9016	AARB vs placebo
**ADVANCE** (Action in Diabetes and Vascular Disease)[7]	2007	11,140	ACEi + diuretic vs placebo
**ALLHAT** (Antihypertensive and Lipid Lowering Treatment to Prevent Heart Attacks Trial)[8]	2002	42,418	Diuretic vs CCB vs ACEi vs alpha-adrenergic blocker
**ALTITUDE** (Aliskiren Trial in Type 2 Diabetes Using Cardiovascular and Renal Disease Endpoints)[9]	2012	8561	Renin inhibitor vs placebo
**ANBP** (The Australian National Blood Pressure Study)[10]	1980	3427	Diuretic ± alpha-adrenergic agonist/BB vs placebo
**ANBP2** (Second Australian National Blood Pressure Study)[11]	2003	6083	ACEi vs diuretic
**APSIS** (Angina Prognosis Study in Stockholm)[12]	1996	809	BB vs CCB
**ASCOT-BPLA** (Anglo-Scandinavian Cardiac Outcomes Trial - Blood Pressure Lowering Arm)[13]	2005	19,257	BB vs CCB
**ATTEMPT-CVD** (A Trial of Telmisartan Prevention of Cardiovascular Diseases)[14]	2016	1228	ARB vs non-ARB
**BBB** (Behandla Blodtryck Battre)[15]	1994	2127	Intensive vs usual BP lowering
**BCAPS** (β-Blocker Cholesterol-Lowering Asymptomatic Plaque Study)[16]	2001	793	BB vs placebo
**BENEDICT** (Bergamo Nephrologic Diabetes Complications Trial)[17]	2004	1808	ACEi, CCB or combination vs placebo; ACEi vs CCB
**CAMELOT** (The Comparison of Amlodipine vs Enalapril to Limit Occurrences of Thrombosis)[18]	2004	3327	CCB or ACEi vs placebo; CCB vs ACEi
**CAPPP** (Captopril Prevention Project)[19]	1999	10,985	ACEi vs diuretic + BB
**Cardio-Sis** (CARDIOvascolari del Controllo della Pressione Arteriosa SIStolica)[20]	2009	1111	Tight vs usual BP lowering
**CASE-J** (Candesartan Antihypertensive Survival Evaluation in Japan)[21]	2008	4703	ARB vs CCB
**CHIEF** (Chinese Hypertension Intervention Efficacy study)[22]	2012	13,080	CCB + diuretic vs CCB + ARB
**COLM** (Combination of OLMesartan and calcium channel blocker or diuretic)[23]	2014	5141	CCB + ARB vs diuretic + ARB
**CONVINCE** (Controlled Onset Verapamil Investigation of Cardiovascular End Points)[24]	2003	16,476	CCB vs BB or diuretic
**Coope J**, et al[25]	1986	884	BB ± diuretic
**COPE** (Combination Therapy of Hypertension to Prevent Cardiovascular Events)[26]	2011	6586	BB + CCB vs ARB + CCB; ARB + CCB vs diuretic + CCB; BB + CCB vs diuretic + CCB
**DIABHYCAR** (Non-insulin-dependent diabetes, hypertension, microalbuminuria or proteinuria, cardiovascular events, and ramipril)[27]	2004	4912	ACEi vs placebo
**DIME** (Diuretics in the Management of Essential hypertension study)[28]	2014	1130	Diuretic vs non-diuretic
**DIRECT-Prevent 1** (Effect of candesartan on prevention)[29]	2008	1421	ARB vs placebo
**DIRECT-Protect 1** (Effect of candesartan on progression)[29]	2008	1905	ARB vs placebo
**DIRECT-Protect 2** (Effect of candesartan on progression and regression of retinopathy in type 2 diabetes)[30]	2011	1905	ARB vs placebo
**DREAM** (Diabetes REduction Assessment with ramipril and rosiglitazone Medication)[31]	2006	5269	ACEi vs placebo
**Dutch TIA Trial** (Dutch Transient Ischemic Attack Trial)[32]	1993	1473	BB vs placebo

For peer review only - http://bmjopen.bmj.com/site/about/guidelines.xhtml

**E-COST** (Efficacy of Candesartan on Outcome in Saitama Trial)[33]	2005	2048	ARB vs conventional drug (other than ACEi or ARB)
ELSA (Efficacy of Candesartan on Outcome in Saitama Trial)[34]	2002	2334	BB vs CCB
**EUROPA** (European trial on reduction of cardiac events with perindopril in stable coronary artery)[35]	2003	12,218	ACEi vs placebo
**EWPHE** (European Working Party on High Blood Pressure in the Elderly)[36]	1985	840	Diuretic vs placebo
**FEVER** (Felodipine Event Reduction Study)[37]	2005	9711	CCB + diuretic vs diuretic
**HAPPHY** (Heart Attack Primary Prevention in Hypertension Trial)[38]	1987	6569	Diuretic vs BB
**HDFP** (Hypertension Detection and Follow Up Program)[39]	1979	10,940	Intensive (stepped care) vs usual (referred care) BP lowering
**HIJ-CREATE** (Heart Institute of Japan Candesartan Randomized Trial for Evaluation in Coronary Heart Disease)[40]	2009	2049	ARB vs non-ARB
**HOMED-BP** (Hypertension Objective Treatment based on Measurement by Electrical Devices of Blood Pressure Study)[41]	2012	3518	Tight BP control, usual BP control, ACEi, CCB and ARB (factorial design)
**HOPE** (Heart Outcomes Prevention Evaluation Study)[42]	2000	9297	ACEi vs placebo
**HOPE-3** (Heart Outcomes Prevention Evaluation-3)[43]	2016	12,705	ARB + diuretic vs placebo
**HOT** (Hypertension Optimal Treatment Study)[44]	1998	18,790	DBP lowering to ≤80, ≤85 and ≤90 mmHg
**HYVET** (Hypertension in the Very Elderly Trial)[45]	2008	3845	Diuretic vs placebo
**IDNT** (Irbesartan Diabetic Nephropathy Trial)[46]	2001	2861	ARB or CCB vs placebo; ARB vs CCB
**INSIGHT** (International Nifedipine GITS Study: Intervention as a Goal for Hypertension Therapy)[47]	2000	6321	CCB vs diuretic
**INVEST** (International Verapamil-Trandolapril Study)[48]	2003	22,576	CCB vs BB
**IPPSH** (International Prospective Primary Prevention Study in Hypertension)[49]	1985	6357	BB vs non-BB
**JATOS** (Japanese Trial to Assess Optimal Systolic Blood Pressure in Elderly Hypertensive Patients)[50]	2008	4418	Strict vs mild BP lowering
**JMIC-B** (Japan Multicenter Investigation for Cardiovascular Diseases-B)[51]	2004	1650	CCB vs ACEi
**LIFE** (Losartan Intervention for Endpoint Reduction in Hypertension Study)[52]	2002	9193	ARB vs BB
**LIT** (Lopressor Intervention Trial)[53]	1987	2395	BB vs placebo
**MIDAS** (Multicenter Isradipine Diuretic Atherosclerosis Study)[54]	1996	883	CCB vs diuretic
**MOSES** (Morbidity and Mortality After Stroke, Eprosartan Compared With Nitrendipine for Secondary Prevention)[55]	2005	1352	ARB vs CCB
**MRC-1** (Medical Research Council Treatment of Mild Hypertension)[56]	1985	26,054	Diuretic or BB vs placebo
**MRC-2** (Medical Research Council Treatment of Hypertension in Older Adults)[57]	1992	6579	Diuretic or BB vs placebo; Diuretic vs BB
**Multicentre International Study**[58]	1975	3038	BB vs placebo
**NAVIGATOR** (Nateglinide and Valsartan in Impaired Glucose Tolerance Outcomes Research)[59]	2010	9306	ARB vs placebo
**NICOLE** (Nisoldipine in Coronary Artery Disease in Leuven Study)[60]	2001	819	CCB vs placebo
**NICS-EH** (National Intervention Cooperative Study in Elderly Hypertensives)[61]	1999	414	CCB vs diuretic
**NORDIL** (Nordic Diltiazem Study)[62]	2000	10,881	CCB vs diuretic
**ONTARGET** (Ongoing Telmisartan Alone and in Combination with Ramipril Global Endpoint Trial)[63]	2008	34,196	ARB + ACEi vs ACEi; ARB vs ACEi
**OSCAR** (OlmeSartan and calcium antagonists randomized study)[64]	2012	1164	ARB vs ARB + CCB
**The Oslo Study**[65]	1980	785	Diuretic ± alpha-adrenergic agonist or BB vs placebo
**PART-2** (Prevention of Atherosclerosis with Ramipril Trial)[66]	2000	617	ACEi vs placebo
**PATE-Hypertension** (The Practitioner's Trial on the Efficacy of Antihypertensive Treatment in the Elderly with Hypertension)[67]	2000	1748	ACEi vs CCB
**PATS** (Post-stroke Antihypertensive Treatment Study)[68]	2009	5665	Diuretic vs placebo

For peer review only

For peer review only - http://bmjopen.bmj.com/site/about/guidelines.xhtml

**PEACE** (Prevention of Events with Angiotensin Converting Enzyme Inhibition)[69]	2004	8290	ACEi vs placebo
**PHARAO** (Prevention of hypertension with the angiotensin-converting enzyme inhibitor ramipril in patients with high-normal blood pressure)[70]	2008	1008	ACEi vs placebo
**PREVEND IT** (Prevention of Renal and Vascular Endstage Disease)[71]	2004	864	ACEi vs placebo
**PREVENT** (Prospective Randomized Evaluation of the Vascular Effects of Norvasc Trial)[72]	2000	825	CCB vs placebo
**PRoFESS** (Prevention Regimen For Effectively Avoiding Second Strokes)[73]	2008	20,332	ARB vs placebo
**PROGRESS** (Perindopril Protection Against Recurrent Stroke Study)[74]	2001	6105	ACEi ± diuretic vs placebo
**QUIET** (Quinapril Ischaemic Event Trial)[75]	2001	1750	ACEi vs placebo
**RENAAL** (Reduction of Endpoints in NIDDM with the Angiotensin II Antagonist Losartan Study)[76]	2001	1513	ARB vs placebo
**ROADMAP** (Randomized Olmesartan And Diabetes Microalbuminuria Prevention Study)[77]	2011	4447	ARB vs placebo
**SCOPE** (Study on Cognition and Prognosis in the Elderly)[78]	2003	4937	ARB vs placebo
**SHELL** (Systolic Hypertension in the Elderly Long-term Lacidipine Trial)[79]	2003	1882	CCB vs diuretic
**SHEP** (Systolic Hypertension in the Elderly Program)[80]	1991	4736	Diuretic ± BB vs placebo
**SPRINT** (Systolic Blood Pressure Intervention Trial)[81]	2015	9361	Intensive vs standard BP lowering
**SPS3** (Secondary Prevention of Small Subcortical Strokes)[82]	2013	3020	SBP lowering to <120 and <140 mmHg
**STONE** (Shanghai trial of nifedipine in the elderly)[83]	1996	1632	CCB vs placebo
**STOP Hypertension** (Swedish Trial in Old Patients with Hypertension)[84]	1991	1627	BB + diuretic vs placebo
**STOP Hypertension-2** (Swedish Trial in Old Patients with Hypertension-2)[85]	1999	13,228	ACEi vs conventional drug; CCB vs conventional drug; ACEi vs CCB
**Sun M**, et al[86]	1997	2080	CCB vs placebo
**Syst-China** (Systolic Hypertension in China)[87]	1988	2394	CCB ± ACEi and/or diuretic vs placebo
**Syst-Eur** (Systolic Hypertension in Europe)[88]	1997	4695	CCB ± ACEi and/or diuretic
**Taylor SH**, et al[89]	1982	1103	BB vs placebo
**TEST** (Ternormin after stroke and TIA)[90]	1995	720	BB vs placebo
**TOMHS** (Treatment of Mild Hypertension Study)[91]	1993	902	Multi-drug class combination vs placebo
**TRANSCEND** (Telmisartan Randomised Assessment Study in ACE Intolerant Subjects with Cardiovascular Disease)[92]	2008	5926	ARB vs placebo
**TROPHY** (Trial of Preventing Hypertension)[93]	2006	772	ARB vs placebo
**UKPDS** (UK Prospective Diabetes Study)[94]	1998	1906	Strict vs less strict BP lowering; ACEi vs BB
**VA NEPHRON-D** (Veterans Affairs Nephropathy in Diabetes)[95]	2013	1448	ACEi + ARB vs ARB
**VALISH** (Valsartan in Elderly Isolated Systolic Hypertension Study)[96]	2010	3079	Strict vs moderate BP lowering
**VALUE** (Valsartan Antihypertensive Long-Term Use Evaluation)[97]	2004	15,245	ARB vs CCB
**VHAS** (Verapamil in Hypertension and Atherosclerosis Study)[98]	1997	1414	Diuretic vs CCB
**Wei Y**, et al.[99]	2013	724	Intensive vs standard BP lowering

BPLTTC – Blood Pressure Lowering Treatment Trialists' Collaboration; ACEi – Angiotensin-converting enzyme inhibitor; ARB – Angiotensin receptor blocker; BB – Beta-blocker; CCB – Calcium channel blocker; BP – blood pressure; SBP – Systolic BP; DBP – Diastolic BP.

For peer review only - http://bmjopen.bmj.com/site/about/guidelines.xhtml

Supplementary table 3 references:

1. Wright JT, Jr., Bakris G, Greene T, et al. Effect of blood pressure lowering and antihypertensive drug class on progression of hypertensive kidney disease: results from the AASK trial. *JAMA* 2002;**288**:2421-31.
2. Estacio RO, Jeffers BW, Hiatt WR, et al. The effect of nisoldipine as compared with enalapril on cardiovascular outcomes in patients with non-insulin-dependent diabetes and hypertension. *New Engl J Med* 1998;**338**:645-52. doi:10.1056/NEJM199803053381003
3. Jamerson K, Weber MA, Bakris GL, et al. Benazepril plus amlodipine or hydrochlorothiazide for hypertension in high-risk patients. *New Engl J Med* 2008;**359**:2417-28. doi:10.1056/NEJMoa0806182
4. ACCORD Study Group, Cushman WC, Evans GW, et al. Effects of intensive blood-pressure control in type 2 diabetes mellitus. *New Engl J Med* 2010;**362**:1575-85. doi:10.1056/NEJMoa1001286
5. Poole-Wilson PA, Lubsen J, Kirwan BA, et al. Effect of long-acting nifedipine on mortality and cardiovascular morbidity in patients with stable angina requiring treatment (ACTION trial): randomised controlled trial. *Lancet* 2004;**364**:849-57. doi:10.1016/S0140-6736(04)16980-8
6. Active I Investigators, Yusuf S, Healey JS, et al. Irbesartan in patients with atrial fibrillation. *New Engl J Med* 2011;**364**:928-38. doi:10.1056/NEJMoa1008816
7. Patel A, Group AC, MacMahon S, et al. Effects of a fixed combination of perindopril and indapamide on macrovascular and microvascular outcomes in patients with type 2 diabetes mellitus (the ADVANCE trial): a randomised controlled trial. *Lancet* 2007;**370**:829-40. doi:10.1016/S0140-6736(07)61303-8
8. Major cardiovascular events in hypertensive patients randomized to doxazosin vs chlorthalidone: the antihypertensive and lipid-lowering treatment to prevent heart attack trial (ALLHAT). ALLHAT Collaborative Research Group. *JAMA* 2000;**283**:1967-75.
9. Parving HH, Brenner BM, McMurray JJ, et al. Cardiorenal end points in a trial of aliskiren for type 2 diabetes. *New Engl J Med* 2012;**367**:2204-13. doi:10.1056/NEJMoa1208799
10. The Australian therapeutic trial in mild hypertension. Report by the Management Committee. *Lancet* 1980;**315**:1261-7.
11. Wing LM, Reid CM, Ryan P, et al. A comparison of outcomes with angiotensin-converting--enzyme inhibitors and diuretics for hypertension in the elderly. *New Engl J Med* 2003;**348**:583-92. doi:10.1056/NEJMoa021716
12. Rehnqvist N, Hjemdahl P, Billing E, et al. Effects of metoprolol vs verapamil in patients with stable angina pectoris. The Angina Prognosis Study in Stockholm (APSIS). *Eur Heart J* 1996;**17**:76-81.
13. Dahlöf B, Sever PS, Poulter NR, et al. Prevention of cardiovascular events with an antihypertensive regimen of amlodipine adding perindopril as required versus atenolol adding bendroflumethiazide as required, in the Anglo-Scandinavian Cardiac Outcomes Trial-Blood Pressure Lowering Arm (ASCOT-BPLA): a multicentre randomised controlled trial. *Lancet* 2005;**366**:895-906. doi:10.1016/s0140-6736(05)67185-1
14. Ogawa H, Soejima H, Matsui K, et al. A trial of telmisartan prevention of cardiovascular diseases (ATTEMPT-CVD): Biomarker study. *Eur J Prev Cardiol* 2016;**23**:913-21. doi:10.1177/2047487315603221
15. Hansson L. The BBB Study: the effect of intensified antihypertensive treatment on the level of blood pressure, side-effects, morbidity and mortality in well-treated hypertensive patients. Behandla Blodtryck Battre. *Blood Pressure* 1994;**3**:248--54.
16. Hedblad B, Wikstrand J, Janzon L, et al. Low-dose metoprolol CR/XL and fluvastatin slow progression of carotid intima-media thickness: Main results from the Beta-Blocker Cholesterol-Lowering Asymptomatic Plaque Study (BCAPS). *Circulation* 2001;**103**:1721-6.
17. Ruggenenti P, Fassi A, Ilieva AP, et al. Preventing microalbuminuria in type 2 diabetes. *New Engl J Med* 2004;**351**:1941-51. doi:10.1056/NEJMoa042167
18. Nissen SE, Tuzcu EM, Libby P, et al. Effect of antihypertensive agents on cardiovascular events in patients with coronary disease and normal blood pressure: the CAMELOT study: a randomized controlled trial. *JAMA* 2004;**292**:2217-25. doi:10.1001/jama.292.18.2217
19. Hansson L, Lindholm LH, Niskanen L, et al. Effect of angiotensin-converting-enzyme inhibition compared with conventional therapy on cardiovascular morbidity and mortality in hypertension: the Captopril Prevention Project (CAPPP) randomised trial. *Lancet* 1999;**353**:611-6.
20. Verdecchia P, Staessen JA, Angeli F, et al. Usual versus tight control of systolic blood pressure in non-diabetic patients with hypertension (Cardio-Sis): an open-label randomised trial. *Lancet* 2009;**374**:525-33. doi:10.1016/S0140-6736(09)61340-4
21. Ogihara T, Nakao K, Fukui T, et al. Effects of candesartan compared with amlodipine in hypertensive patients with high cardiovascular risks: candesartan antihypertensive survival evaluation in Japan trial. *Hypertension* 2008;**51**:393-8. doi:10.1161/HYPERTENSIONAHA.107.098475
22. Ma L, Wang W, Zhao Y, et al. Combination of amlodipine plus angiotensin receptor blocker or diuretics in high-risk hypertensive patients: a 96-week efficacy and safety study. *Am J Cardiovasc Drugs* 2012;**12**:137-42. doi:10.2165/11598110-000000000-00000

For peer review only

23. Ogihara T, Saruta T, Rakugi H, et al. Combinations of olmesartan and a calcium channel blocker or a diuretic in elderly hypertensive patients: a randomized, controlled trial. *J Hypertens* 2014;**32**:2054-63; discussion 63. doi:10.1097/HJH.0000000000000281
24. Black HR, Elliott WJ, Grandits G, et al. Principal results of the Controlled Onset Verapamil Investigation of Cardiovascular End Points (CONVINCE) trial. *JAMA* 2003;**289**:2073-82. doi:10.1001/jama.289.16.2073
25. Coope J, Warrender TS. Randomised trial of treatment of hypertension in elderly patients in primary care. *BMJ* 1986;**293**:1145-51.
26. Matsuzaki M, Ogihara T, Umemoto S, et al. Prevention of cardiovascular events with calcium channel blocker-based combination therapies in patients with hypertension: a randomized controlled trial. *J Hypertens* 2011;**29**:1649-59. doi:10.1097/HJH.0b013e328348345d
27. Marre M, Lievre M, Chatellier G, et al. Effects of low dose ramipril on cardiovascular and renal outcomes in patients with type 2 diabetes and raised excretion of urinary albumin: randomised, double blind, placebo controlled trial (the DIABHYCAR study). *BMJ* 2004;**328**:495. doi:10.1136/bmj.37970.629537.0D
28. Ueda S, Morimoto T, Ando S, et al. A randomised controlled trial for the evaluation of risk for type 2 diabetes in hypertensive patients receiving thiazide diuretics: Diuretics In the Management of Essential hypertension (DIME) study. *BMJ Open* 2014;**4**:e004576. doi:10.1136/bmjopen-2013-004576
29. Chaturvedi N, Porta M, Klein R, et al. Effect of candesartan on prevention (DIRECT-Prevent 1) and progression (DIRECT-Protect 1) of retinopathy in type 1 diabetes: randomised, placebo-controlled trials. *Lancet* 2008;**372**:1394-402. doi:10.1016/S0140-6736(08)61412-9
30. Tillin T, Orchard T, Malm A, et al. The role of antihypertensive therapy in reducing vascular complications of type 2 diabetes. Findings from the DIabetic REtinopathy Candesartan Trials-Protect 2 study. *J Hypertens* 2011;**29**:1457-62. doi:10.1097/HJH.0b013e3283480db9
31. DREAM Trial Investigators, Bosch J, Yusuf S, et al. Effect of ramipril on the incidence of diabetes. *New Engl J Med* 2006;**355**:1551-62. doi:10.1056/NEJMoa065061
32. Trial of secondary prevention with atenolol after transient ischemic attack or nondisabling ischemic stroke. The Dutch TIA Trial Study Group. *Stroke* 1993;**24**:543-8.
33. Suzuki H, Kanno Y, Group EoCoOiST. Effects of candesartan on cardiovascular outcomes in Japanese hypertensive patients. *Hypertens Res* 2005;**28**:307-14. doi:10.1291/hypres.28.307
34. Zanchetti A, Bond MG, Hennig M, et al. Calcium antagonist lacidipine slows down progression of asymptomatic carotid atherosclerosis: principal results of the European Lacidipine Study on Atherosclerosis (ELSA), a randomized, double-blind, long-term trial. *Circulation* 2002;**106**:2422-7.
35. Fox KM, Investigators EtOrocewPiscAd. Efficacy of perindopril in reduction of cardiovascular events among patients with stable coronary artery disease: randomised, double-blind, placebo-controlled, multicentre trial (the EUROPA study). *Lancet* 2003;**362**:782-8.
36. Amery A, Birkenhager W, Brixko P, et al. Mortality and morbidity results from the European Working Party on High Blood Pressure in the Elderly trial. *Lancet* 1985;**1**:1349-54.
37. Liu L, Zhang Y, Liu G, et al. The Felodipine Event Reduction (FEVER) Study: a randomized long-term placebo-controlled trial in Chinese hypertensive patients. *J Hypertens* 2005;**23**:2157-72.
38. Wilhelmsen L, Berglund G, Elmfeldt D, et al. Beta-blockers versus diuretics in hypertensive men: main results from the HAPPHY trial. *J Hypertens* 1987;**5**:561-72.
39. Five-year findings of the hypertension detection and follow-up program. I. Reduction in mortality of persons with high blood pressure, including mild hypertension. Hypertension Detection and Follow-up Program Cooperative Group. *JAMA* 1979;**242**:2562-71.
40. Kasanuki H, Hagiwara N, Hosoda S, et al. Angiotensin II receptor blocker-based vs. non-angiotensin II receptor blocker-based therapy in patients with angiographically documented coronary artery disease and hypertension: the Heart Institute of Japan Candesartan Randomized Trial for Evaluation in Coronary Artery Disease (HIJ-CREATE). *Eur Heart J* 2009;**30**:1203-12. doi:10.1093/eurheartj/ehp101
41. Asayama K, Ohkubo T, Metoki H, et al. Cardiovascular outcomes in the first trial of antihypertensive therapy guided by self-measured home blood pressure. *Hypertens Res* 2012;**35**:1102-10. doi:10.1038/hr.2012.125
42. Investigators; HOPES, Yusuf S, Sleight P, et al. Effects of an angiotensin-converting-enzyme inhibitor, ramipril, on cardiovascular events in high-risk patients. *New Engl J Med* 2000;**342**:145-53. doi:10.1056/NEJM200001203420301
43. Lonn EM, Bosch J, Lopez-Jaramillo P, et al. Blood-Pressure Lowering in Intermediate-Risk Persons without Cardiovascular Disease. *New Engl J Med* 2016;**374**:2009-20. doi:10.1056/NEJMoa1600175
44. Hansson L, Zanchetti A, Carruthers SG, et al. Effects of intensive blood-pressure lowering and low-dose aspirin in patients with hypertension: principal results of the Hypertension Optimal Treatment (HOT) randomised trial. HOT Study Group. *Lancet* 1998;**351**:1755--62.
45. Beckett NS, Peters R, Fletcher AE, et al. Treatment of hypertension in patients 80 years of age or older. *New Engl J Med* 2008;**358**:1887-98. doi:10.1056/NEJMoa0801369

For peer review only

46. Lewis EJ, Hunsicker LG, Clarke WR, et al. Renoprotective effect of the angiotensin-receptor antagonist irbesartan in patients with nephropathy due to type 2 diabetes. *New Engl J Med* 2001;**345**:851-60. doi:10.1056/NEJMoa011303
47. Brown MJ, Palmer CR, Castaigne A, et al. Morbidity and mortality in patients randomised to double-blind treatment with a long-acting calcium-channel blocker or diuretic in the International Nifedipine GITS study: Intervention as a Goal in Hypertension Treatment (INSIGHT). *Lancet* 2000;**356**:366-72. doi:10.1016/S0140-6736(00)02527-7
48. Pepine CJ, Handberg EM, Cooper-DeHoff RM, et al. A calcium antagonist vs a non-calcium antagonist hypertension treatment strategy for patients with coronary artery disease. The International Verapamil-Trandolapril Study (INVEST): a randomized controlled trial. *JAMA* 2003;**290**:2805-16. doi:10.1001/jama.290.21.2805
49. Cardiovascular risk and risk factors in a randomized trial of treatment based on the beta-blocker oxprenolol: the International Prospective Primary Prevention Study in Hypertension (IPPPSH). The IPPPSH Collaborative Group. *J Hypertens* 1985;**3**:379-92.
50. JATOS Study Group. Principal results of the Japanese trial to assess optimal systolic blood pressure in elderly hypertensive patients (JATOS). *Hypertens Res* 2008;**31**:2115-27. doi:10.1291/hypres.31.2115
51. Yui Y, Sumiyoshi T, Kodama K, et al. Comparison of nifedipine retard with angiotensin converting enzyme inhibitors in Japanese hypertensive patients with coronary artery disease: the Japan Multicenter Investigation for Cardiovascular Diseases-B (JMIC-B) randomized trial. *Hypertens Res* 2004;**27**:181-91.
52. Dahlof B, Devereux RB, Kjeldsen SE, et al. Cardiovascular morbidity and mortality in the Losartan Intervention For Endpoint reduction in hypertension study (LIFE): a randomised trial against atenolol. *Lancet* 2002;**359**:995-1003. doi:10.1016/S0140-6736(02)08089-3
53. The Lopressor Intervention Trial: multicentre study of metoprolol in survivors of acute myocardial infarction. Lopressor Intervention Trial Research Group. *Eur Heart J* 1987;**8**:1056-64.
54. Borhani NO, Mercuri M, Borhani PA, et al. Final outcome results of the Multicenter Isradipine Diuretic Atherosclerosis Study (MIDAS). A randomized controlled trial. *JAMA* 1996;**276**:785-91.
55. Schrader J, Luders S, Kulschewski A, et al. Morbidity and Mortality After Stroke, Eprosartan Compared with Nitrendipine for Secondary Prevention: principal results of a prospective randomized controlled study (MOSES). *Stroke* 2005;**36**:1218-26. doi:10.1161/01.STR.0000166048.35740.a9
56. MRC trial of treatment of mild hypertension: principal results. Medical Research Council Working Party. *BMJ* 1985;**291**:97-104.
57. Medical Research Council trial of treatment of hypertension in older adults: principal results. MRC Working Party. *BMJ* 1992;**304**:405-12.
58. Improvement in prognosis of myocardial infarction by long-term beta-adrenoreceptor blockade using practolol. A multicentre international study. *BMJ* 1975;**3**:735-40.
59. Navigator Study Group, McMurray JJ, Holman RR, et al. Effect of valsartan on the incidence of diabetes and cardiovascular events. *New Engl J Med* 2010;**362**:1477-90. doi:10.1056/NEJMoa1001121
60. Dens JA, Desmet WJ, Coussement P, et al. Long term effects of nisoldipine on the progression of coronary atherosclerosis and the occurrence of clinical events: the NICOLE study. *Heart* 2003;**89**:887-92.
61. Randomized double-blind comparison of a calcium antagonist and a diuretic in elderly hypertensives. National Intervention Cooperative Study in Elderly Hypertensives Study Group. *Hypertension* 1999;**34**:1129-33.
62. Hansson L, Hedner T, Lund-Johansen P, et al. Randomised trial of effects of calcium antagonists compared with diuretics and beta-blockers on cardiovascular morbidity and mortality in hypertension: the Nordic Diltiazem (NORDIL) study. *Lancet* 2000;**356**:359-65.
63. ONTARGET Investigators, Yusuf S, Teo KK, et al. Telmisartan, ramipril, or both in patients at high risk for vascular events. *New Engl J Med* 2008;**358**:1547-59. doi:10.1056/NEJMoa0801317
64. Ogawa H, Kim-Mitsuyama S, Matsui K, et al. Angiotensin II receptor blocker-based therapy in Japanese elderly, high-risk, hypertensive patients. *Am J Med* 2012;**125**:981-90. doi:10.1016/j.amjmed.2011.12.010
65. Helgeland A. Treatment of mild hypertension: a five year controlled drug trial. The Oslo study. *Am J Med* 1980;**69**:725-32.
66. MacMahon S, Sharpe N, Gamble G, et al. Randomized, placebo-controlled trial of the angiotensin-converting enzyme inhibitor, ramipril, in patients with coronary or other occlusive arterial disease. PART-2 Collaborative Research Group. Prevention of Atherosclerosis with Ramipril. *J Am Coll Cardiol* 2000;**36**:438-43.
67. Ogihara T. Practitioner's Trial on the Efficacy of Antihypertensive Treatment in the Elderly Hypertension (The PATE-Hypertension Study) in Japan. *Am J Hypertens* 2000;**13**:461-7.
68. Liu L, Wang Z, Gong L, et al. Blood pressure reduction for the secondary prevention of stroke: a Chinese trial and a systematic review of the literature. *Hypertens Research* 2009;**32**:1032-40. doi:10.1038/hr.2009.139
69. Braunwald E, Domanski MJ, Fowler SE, et al. Angiotensin-converting-enzyme inhibition in stable coronary artery disease. *New Engl J Med* 2004;**351**:2058-68. doi:10.1056/NEJMoa042739
70. Luders S, Schrader J, Berger J, et al. The PHARAO study: prevention of hypertension with the angiotensin-converting enzyme inhibitor ramipril in patients with high-normal blood pressure: a prospective, randomized, controlled prevention trial of the German Hypertension League. *J Hypertens* 2008;**26**:1487-96. doi:10.1097/HJH.0b013e3282ff8864

For peer review only

For peer review only - http://bmjopen.bmj.com/site/about/guidelines.xhtml

71. Asselbergs FW, Diercks GF, Hillege HL, et al. Effects of fosinopril and pravastatin on cardiovascular events in subjects with microalbuminuria. *Circulation* 2004;**110**:2809-16. doi:10.1161/01.CIR.0000146378.65439.7A
72. Pitt B, Byington RP, Furberg CD, et al. Effect of amlodipine on the progression of atherosclerosis and the occurrence of clinical events. PREVENT Investigators. *Circulation* 2000;**102**:1503-10.
73. Yusuf S, Diener HC, Sacco RL, et al. Telmisartan to prevent recurrent stroke and cardiovascular events. *New Engl J Med* 2008;**359**:1225-37. doi:10.1056/NEJMoa0804593
74. Randomised trial of a perindopril-based blood-pressure-lowering regimen among 6105 individuals with previous stroke or transient ischaemic attack. *Lancet* 2001;**358**:1033-41. doi:10.1016/s0140-6736(01)06178-5
75. Pitt B, O'Neill B, Feldman R, et al. The QUinapril Ischemic Event Trial (QUIET): evaluation of chronic ACE inhibitor therapy in patients with ischemic heart disease and preserved left ventricular function. *Am J Cardiol* 2001;**87**:1058-63.
76. Brenner BM, Cooper ME, de Zeeuw D, et al. Effects of losartan on renal and cardiovascular outcomes in patients with type 2 diabetes and nephropathy. *New Engl J Med* 2001;**345**:861-9. doi:10.1056/NEJMoa011161
77. Haller H, Ito S, Izzo JL, Jr., et al. Olmesartan for the delay or prevention of microalbuminuria in type 2 diabetes. *New Engl J Med* 2011;**364**:907-17. doi:10.1056/NEJMoa1007994
78. Lithell H, Hansson L, Skoog I, et al. The Study on Cognition and Prognosis in the Elderly (SCOPE): principal results of a randomized double-blind intervention trial. *J Hypertens* 2003;**21**:875-86. doi:10.1097/01.hjh.0000059028.82022.89
79. Malacco E, Mancia G, Rappelli A, et al. Treatment of isolated systolic hypertension: the SHELL study results. *Blood Pressure* 2003;**12**:160-7.
80. Prevention of stroke by antihypertensive drug treatment in older persons with isolated systolic hypertension. Final results of the Systolic Hypertension in the Elderly Program (SHEP). SHEP Cooperative Research Group. *JAMA* 1991;**265**:3255-64.
81. SPRINT Research Group, Wright JT, Jr., Williamson JD, et al. A Randomized Trial of Intensive versus Standard Blood-Pressure Control. *New Engl J Med* 2015;**373**:2103-16. doi:10.1056/NEJMoa1511939
82. S. P. S. Study Group, Benavente OR, Coffey CS, et al. Blood-pressure targets in patients with recent lacunar stroke: the SPS3 randomised trial. *Lancet* 2013;**382**:507-15. doi:10.1016/S0140-6736(13)60852-1
83. Gong L, Zhang W, Zhu Y, et al. Shanghai trial of nifedipine in the elderly (STONE). *J Hypertens* 1996;**14**:1237-45.
84. Dahlof B, Lindholm LH, Hansson L, et al. Morbidity and mortality in the Swedish Trial in Old Patients with Hypertension (STOP-Hypertension). *Lancet* 1991;**338**:1281-5.
85. Hansson L, Lindholm LH, Ekbom T, et al. Randomised trial of old and new antihypertensive drugs in elderly patients: cardiovascular mortality and morbidity the Swedish Trial in Old Patients with Hypertension-2 study. *Lancet* 1999;**354**:1751-6.
86. Sun M, Zhou H, Jia Z. [Prevention and treatment of stroke after hypertension for ten years in Hunan Province]. *Zhonghua nei ke za zhi* 1997;**36**:312-4.
87. Liu L, Wang JG, Gong L, et al. Comparison of active treatment and placebo in older Chinese patients with isolated systolic hypertension. Systolic Hypertension in China (Syst-China) Collaborative Group. *J Hypertens* 1998;**16**:1823-9.
88. Staessen JA, Fagard R, Thijs L, et al. Randomised double-blind comparison of placebo and active treatment for older patients with isolated systolic hypertension. The Systolic Hypertension in Europe (Syst-Eur) Trial Investigators. *Lancet* 1997;**350**:757-64.
89. Taylor SH, Silke B, Ebbutt A, et al. A long-term prevention study with oxprenolol in coronary heart disease. *New Engl J Med* 1982;**307**:1293-301. doi:10.1056/NEJM198211183072101
90. Eriksson S, Olofsson BO, Wester PO, et al. Atenolol in Secondary Prevention after Stroke. *Cerebrovasc Dis* 1995;**5**:21-25. doi:https://doi.org/10.1159/000107813
91. Neaton JD, Grimm RH, Jr., Prineas RJ, et al. Treatment of Mild Hypertension Study. Final results. Treatment of Mild Hypertension Study Research Group. *JAMA* 1993;**270**:713-24.
92. Telmisartan Randomised AssessmeNt Study in A. C. E. iNtolerant subjects with cardiovascular Disease Investigators, Yusuf S, Teo K, et al. Effects of the angiotensin-receptor blocker telmisartan on cardiovascular events in high-risk patients intolerant to angiotensin-converting enzyme inhibitors: a randomised controlled trial. *Lancet* 2008;**372**:1174-83. doi:10.1016/S0140-6736(08)61242-8
93. Julius S, Nesbitt SD, Egan BM, et al. Feasibility of treating prehypertension with an angiotensin-receptor blocker. *The New Engl J Med* 2006;**354**:1685-97. doi:10.1056/NEJMoa060838
94. Tight blood pressure control and risk of macrovascular and microvascular complications in type 2 diabetes: UKPDS 38. UK Prospective Diabetes Study Group. *BMJ* 1998;**317**:703-13.
95. Fried LF, Emanuele N, Zhang JH, et al. Combined angiotensin inhibition for the treatment of diabetic nephropathy. *The New Engl J Med* 2013;**369**:1892-903. doi:10.1056/NEJMoa1303154
96. Ogihara T, Saruta T, Rakugi H, et al. Target blood pressure for treatment of isolated systolic hypertension in the elderly: valsartan in elderly isolated systolic hypertension study. *Hypertension* 2010;**56**:196-202. doi:10.1161/HYPERTENSIONAHA.109.146035

For peer review only

97. Julius S, Kjeldsen SE, Weber M, et al. Outcomes in hypertensive patients at high cardiovascular risk treated with regimens based on valsartan or amlodipine: the VALUE randomised trial. *Lancet* 2004;**363**:2022-31. doi:10.1016/s0140-6736(04)16451-9
98. Rosei EA, Dal Palu C, Leonetti G, et al. Clinical results of the Verapamil in Hypertension and Atherosclerosis Study. VHAS Investigators. *J Hypertens* 1997;**15**:1337-44.
99. Wei Y, Jin Z, Shen G, et al. Effects of intensive antihypertensive treatment on Chinese hypertensive patients older than 70 years. *J Clin Hypertens* 2013;**15**:420-7. doi:10.1111/jch.12094

For peer review only

For peer review only - http://bmjopen.bmj.com/site/about/guidelines.xhtml

Supplementary table 4. Variables to be sought from participating trials.

Data	Variables
Study level data	Region<br>Treatment/Comparison groups<br>Study period / Duration of follow-up<br>Randomisation method/Treatment allocation<br>Outcome ascertainment<br>Early stopping and reasons<br>Funding source
Participant level data	<u>Baseline characteristics</u><br>Sex, age, ethnicity, lifestyle (e.g. smoking, alcohol intake), past medical history (e.g. diabetes, cardiovascular disease, chronic renal disease), drug treatment (e.g. hypertension, dyslipidaemia, cardiovascular disease) weight, height, systolic and diastolic blood pressure, blood and urine measurements (e.g. lipids, glucose homestasis, renal function)<br><br><u>Follow-up variables (after randomisation)</u><br>Outcomes and relevant dates (e.g. cardiovascular events, acute renal injury / renal replacement therapy, cancer, fractures, diabetes, retinopathy, neurodegenerative conditions, serious adverse events)<br>Vital status (and cause of death) on last follow-up<br>Clinical measures and relevant dates (e.g. weight, height, systolic and diastolic blood pressure, blood and urine measurements)<br>Changes in treatment (e.g. discontinuation) and date

For peer review only

AMSTAR 2: a critical appraisal tool for systematic reviews that include randomised or non-randomised studies of healthcare interventions, or both

1. Did the research questions and inclusion criteria for the review include the components of PICO?

For Yes:
- ☑ Population
- ☑ Intervention
- ☑ Comparator group
- ☑ Outcome

Optional (recommended)
- ☑ Timeframe for follow-up

- ☑ Yes
- ☐ No

2. Did the report of the review contain an explicit statement that the review methods were established prior to the conduct of the review and did the report justify any significant deviations from the protocol?

For Partial Yes:
The authors state that they had a written protocol or guide that included ALL the following:
- ☑ review question(s)
- ☑ a search strategy
- ☑ inclusion/exclusion criteria
- ☑ a risk of bias assessment

For Yes:
As for partial yes, plus the protocol should be registered and should also have specified:
- ☑ a meta-analysis/synthesis plan, if appropriate, *and*
- ☑ a plan for investigating causes of heterogeneity
- ☐ justification for any deviations from the protocol

- ☑ Yes
- ☐ Partial Yes
- ☐ No

3. Did the review authors explain their selection of the study designs for inclusion in the review?

For Yes, the review should satisfy ONE of the following:
- ☑ *Explanation for* including only RCTs
- ☐ OR *Explanation for* including only NRSI
- ☐ OR *Explanation for* including both RCTs and NRSI

- ☑ Yes
- ☐ No

4. Did the review authors use a comprehensive literature search strategy?

For Partial Yes (all the following):
- ☑ searched at least 2 databases (relevant to research question)
- ☑ provided key word and/or search strategy
- ☑ justified publication restrictions (e.g. language)

For Yes, should also have (all the following):
- ☑ searched the reference lists / bibliographies of included studies
- ☑ searched trial/study registries
- ☑ included/consulted content experts in the field
- ☑ where relevant, searched for grey literature
- ☑ conducted search within 24 months of completion of the review

- ☑ Yes
- ☐ Partial Yes
- ☐ No

5. Did the review authors perform study selection in duplicate?

For Yes, either ONE of the following:
- ☑ at least two reviewers independently agreed on selection of eligible studies and achieved consensus on which studies to include
- ☐ OR two reviewers selected a sample of eligible studies and achieved good agreement (at least 80 percent), with the remainder selected by one reviewer.

- ☑ Yes
- ☐ No

For peer review only

AMSTAR 2: a critical appraisal tool for systematic reviews that include randomised or non-randomised studies of healthcare interventions, or both

This section onwards are currently not relevant to the protocol.

6. Did the review authors perform data extraction in duplicate?

For Yes, either ONE of the following:	
☐ at least two reviewers achieved consensus on which data to extract from included studies<br>☐ OR two reviewers extracted data from a sample of eligible studies <u>and</u> achieved good agreement (at least 80 percent), with the remainder extracted by one reviewer.	☐ Yes<br>☐ No

7. Did the review authors provide a list of excluded studies and justify the exclusions?

For Partial Yes:	For Yes, must also have:	
☐ provided a list of all potentially relevant studies that were read in full-text form but excluded from the review	☐ Justified the exclusion from the review of each potentially relevant study	☐ Yes<br>☐ Partial Yes<br>☐ No

8. Did the review authors describe the included studies in adequate detail?

For Partial Yes (ALL the following):	For Yes, should also have ALL the following:	
☐ described populations<br>☐ described interventions<br>☐ described comparators<br>☐ described outcomes<br>☐ described research designs	☐ described population in detail<br>☐ described intervention in detail (including doses where relevant)<br>☐ described comparator in detail (including doses where relevant)<br>☐ described study's setting<br>☐ timeframe for follow-up	☐ Yes<br>☐ Partial Yes<br>☐ No

9. Did the review authors use a satisfactory technique for assessing the risk of bias (RoB) in individual studies that were included in the review?

**RCTs**<br>For Partial Yes, must have assessed RoB from	For Yes, must also have assessed RoB from:	
☐ unconcealed allocation, *and*<br>☐ lack of blinding of patients and assessors when assessing outcomes (unnecessary for objective outcomes such as all-cause mortality)	☐ allocation sequence that was not truly random, *and*<br>☐ selection of the reported result from among multiple measurements or analyses of a specified outcome	☐ Yes<br>☐ Partial Yes<br>☐ No<br>☐ Includes only NRSI
**NRSI**<br>For Partial Yes, must have assessed RoB:	For Yes, must also have assessed RoB:	
☐ from confounding, *and*<br>☐ from selection bias	☐ methods used to ascertain exposures and outcomes, *and*<br>☐ selection of the reported result from among multiple measurements or analyses of a specified outcome	☐ Yes<br>☐ Partial Yes<br>☐ No<br>☐ Includes only RCTs

10. Did the review authors report on the sources of funding for the studies included in the review?

For Yes	
☐ Must have reported on the sources of funding for individual studies included in the review. Note: Reporting that the reviewers looked for this information but it was not reported by study authors also qualifies	☐ Yes<br>☐ No

For peer review only

AMSTAR 2: a critical appraisal tool for systematic reviews that include randomised or non-randomised studies of healthcare interventions, or both

11. If meta-analysis was performed did the review authors use appropriate methods for statistical combination of results?

RCTs
For Yes:

- ☐ The authors justified combining the data in a meta-analysis
  - ☐ AND they used an appropriate weighted technique to combine study results and adjusted for heterogeneity if present.
  - ☐ AND investigated the causes of any heterogeneity

☐ Yes
☐ No
☐ No meta-analysis conducted

For NRSI
For Yes:

- ☐ The authors justified combining the data in a meta-analysis
  - ☐ AND they used an appropriate weighted technique to combine study results, adjusting for heterogeneity if present
  - ☐ AND they statistically combined effect estimates from NRSI that were adjusted for confounding, rather than combining raw data, or justified combining raw data when adjusted effect estimates were not available
  - ☐ AND they reported separate summary estimates for RCTs and NRSI separately when both were included in the review

☐ Yes
☐ No
☐ No meta-analysis conducted

12. If meta-analysis was performed, did the review authors assess the potential impact of RoB in individual studies on the results of the meta-analysis or other evidence synthesis?

For Yes:

- ☐ included only low risk of bias RCTs
- ☐ OR, if the pooled estimate was based on RCTs and/or NRSI at variable RoB, the authors performed analyses to investigate possible impact of RoB on summary estimates of effect.

☐ Yes
☐ No
☐ No meta-analysis conducted

13. Did the review authors account for RoB in individual studies when interpreting/ discussing the results of the review?

For Yes:

- ☐ included only low risk of bias RCTs
- ☐ OR, if RCTs with moderate or high RoB, or NRSI were included the review provided a discussion of the likely impact of RoB on the results

☐ Yes
☐ No

14. Did the review authors provide a satisfactory explanation for, and discussion of, any heterogeneity observed in the results of the review?

For Yes:

- ☐ There was no significant heterogeneity in the results
- ☐ OR if heterogeneity was present the authors performed an investigation of sources of any heterogeneity in the results and discussed the impact of this on the results of the review

☐ Yes
☐ No

15. If they performed quantitative synthesis did the review authors carry out an adequate investigation of publication bias (small study bias) and discuss its likely impact on the results of the review?

For Yes:

- ☐ performed graphical or statistical tests for publication bias and discussed the likelihood and magnitude of impact of publication bias

☐ Yes
☐ No
☐ No meta-analysis conducted

For peer review only

AMSTAR 2: a critical appraisal tool for systematic reviews that include randomised or non-randomised studies of healthcare interventions, or both

16. Did the review authors report any potential sources of conflict of interest, including any funding they received for conducting the review?

For Yes:	
☐ The authors reported no competing interests OR	☐ Yes
☐ The authors described their funding sources and how they managed potential conflicts of interest	☐ No

To cite this tool: Shea BJ, Reeves BC, Wells G, Thuku M, Hamel C, Moran J, Moher D, Tugwell P, Welch V, Kristjansson E, Henry DA. AMSTAR 2: a critical appraisal tool for systematic reviews that include randomised or non-randomised studies of healthcare interventions, or both. BMJ. 2017 Sep 21;358:j4008.

For peer review only

BMJ Open

Investigating the stratified efficacy and safety of pharmacological blood pressure lowering: An overall protocol for individual patient-level data meta-analyses of over 300,000 randomized participants in the new phase of the Blood Pressure Lowering Treatment Trialists' Collaboration (BPLTTC)

Journal:	*BMJ Open*
Manuscript ID	bmjopen-2018-028698.R1
Article Type:	Protocol
Date Submitted by the Author:	01-Mar-2019
Complete List of Authors:	Rahimi, Kazem; University of Oxford, The George Institute for Global Health UK<br>Canoy, Dexter; University of Oxford, The George Institute for Global Health UK<br>Nazarzadeh, Milad; University of Oxford, The George Institute for Global Health UK<br>Khorshidi, Gholamreza; University of Oxford, The George Institute for Global Health UK<br>Woodward, Mark; University of New South Wales<br>Teo, Koon; Population Health Research Institute<br>Davis, Barry; University of Texas Health Science Center at Houston<br>Chalmers, John; George Institute for Global Health<br>Pepine, Carl; University of Florida
**Primary Subject Heading**:	Epidemiology
Secondary Subject Heading:	Cardiovascular medicine
Keywords:	Blood Pressure, Meta-analyses, Efficacy and safety of BP lowering treatment

SCHOLARONE™
Manuscripts

Investigating the stratified efficacy and safety of pharmacological blood pressure lowering: An overall protocol for individual patient-level data meta-analyses of over 300,000 randomized participants in the new phase of the Blood Pressure Lowering Treatment Trialists' Collaboration (BPLTTC)

Kazem Rahimi,[1] Dexter Canoy,[1,2] Milad Nazarzadeh,[1,3] Gholamreza Khorshidi,[1] Mark Woodward,[1,4] Koon Teo,[5] Barry R. Davis,[6] John Chalmers,[4] Carl J. Pepine,[7] on behalf of the Blood Pressure Lowering Treatment Trialists' Collaboration*

**Individual members listed at the end of the manuscript*

[1]The George Institute for Global Health UK, University of Oxford, Oxford, UK

[2] Faculty of Medicine, University of New South Wales, Sydney, Australia

[3] Collaboration Center of Meta-Analysis Research, Torbat Heydariyeh University of Medical Sciences, Torbat Heydariyeh, Iran

[4]The George Institute for Global Health, University of New South Wales, Sydney, Australia

[5]Population Health Research Institute, McMaster University, Hamilton, Ontario, Canada

[6]The University of Texas School of Public Health, Houston, Texas, USA

[7]Department of Medicine, University of Florida, Gainesville, Florida, USA

Correspondence to:

For peer review only - http://bmjopen.bmj.com/site/about/guidelines.xhtml

The BPLTTC
The George Institute for Global Health (UK)
University of Oxford
Hayes House, 75 George St., Oxford OX1 2BQ, United Kingdom
Tel: +441865617200; fax: +441865617202; e-mail: kazem.rahimi@georgeinstitute.ox.ac.uk

Abstract

Introduction: Previous research from the Blood Pressure Lowering Treatment Trialists' Collaboration (BPLTTC) and others has shown that pharmacological blood pressure (BP) lowering substantially reduces the risk of major cardiovascular events, including ischaemic heart disease, heart failure and stroke. In this new phase, the aim is to conduct individual patient-level data (IPD) meta-analyses involving eligible BP lowering randomized controlled trials (RCTs) to address uncertainties relating to efficacy and safety of BP lowering treatment.

Methods and analysis: RCTs investigating the effect of pharmacological BP lowering, with a minimum of 1000 patient-years of follow-up in each trial arm, are eligible. Our systematic review identified 100 potentially eligible trials. We requested their investigators/sponsors to contribute baseline, follow-up and outcomes data. As of January 2018, the collaboration has obtained data from 49 trials (N=315,046 participants), with additional data currently in the process of being transferred from four RCTs (N=34,642 participants). In addition, data harmonization has commenced. Scientific activities of the collaboration are overseen by the Steering Committee with input from all collaborators. Detailed protocols for individual meta-analyses will be developed and registered on public platforms.

Ethics and dissemination: Ethics approval has been obtained for this new and extended phase of the BPLTTC, the largest collaboration of de-identified IPD from RCTs. It offers an efficient and ethical manner of re-purposing existing data to answer clinically important questions relating to BP treatment as well as methodological questions relating to IPD meta-analyses. Among the immediate impacts will include reliable quantification of effects of treatment modifiers, such as baseline BP, age and prior disease, on both vascular and non-vascular outcomes. Analyses will further assess the impact of BP lowering on important, but less-well understood, outcomes, such as new-onset diabetes and renal disease. Findings will be published in peer-reviewed medical journals on behalf of the collaboration.

For peer review only - http://bmjopen.bmj.com/site/about/guidelines.xhtml

For peer review only

For peer review only - http://bmjopen.bmj.com/site/about/guidelines.xhtml

Strengths and limitations of this study

- This protocol aims to conduct individual patient-level data (IPD) of blood pressure (BP)-lowering randomised clinical trials (RCT) to address stratified efficacy of BP-lowering treatment according to various patient characteristics, examine impact of BP-lowering treatment on less-studied health vascular outcomes, and identify unintended consequences of such treatment.
- With data from 49 RCTs (>300,000 participants) already shared in the collaboration, it will be the largest IPD meta-analysis of any randomised trials, allowing sufficient statistical power to address a number of important clinical questions a single BP-lowering randomised trial is unlikely to be able to answer reliably on its own.
- Findings of meta-analyses from this resource are likely to provide reliable evidence to inform clinical guidelines and public health policy developmemt
- Data from some RCTs are not accessible because some investigators, particularly of older trials, could not be traced; or, sponsors/funders of the trials are unwilling to share data
- Not all RCTs would have collected the same clinical information so missing data is inevitable for some studies; appropriate methods to address these issues would have to be utilised

For peer review only - http://bmjopen.bmj.com/site/about/guidelines.xhtml

Introduction

Elevated blood pressure (BP) is a major risk factor for cardiovascular death and disability worldwide, and its burden has been increasing. In 2016, elevated blood pressure above the minimum risk exposure threshold of 110 mmHg systolic was responsible for more than 10 million deaths and 7% of the world's total disability-adjusted life years (DALY) [1].

Several randomized trials and their meta-analyses have shown that pharmacological BP lowering is an effective and affordable strategy for management of cardiovascular risk. Despite this, treatment and control rates among those who have been classified to have 'hypertension' (BP >140/90 mmHg) remain low in both high and low income countries [2]. This gap between evidence and practice is likely to be at least in part due to remaining uncertainties about the efficacy and safety of BP lowering [3]. For instance, there is no expert consensus on the appropriate BP threshold for treatment initiation and whether this should differ by age or disease status (e.g. diabetes or coronary heart disease [CHD]). Also, little is known on the effects of BP lowering on safety outcomes (e.g. cancer, falls and fractures, acute kidney injury), non-vascular outcomes (e.g. new-onset diabetes, dementia), or less commonly reported vascular outcomes (e.g. atrial fibrillation, peripheral arterial disease, aortic aneurysms, pulmonary embolism, stroke subtype).

These existing uncertainties have major consequences for patient management and outcomes. On the one hand, a lack of reliable estimates for the efficacy of BP lowering on less commonly reported vascular outcomes, some of which have a clear association with BP in observational studies [4–7], may result in an underestimation of the overall efficacy of BP lowering in the general patient population. On the other hand, uncertainty about the safety of BP lowering could potentially expose specific patient groups to avoidable harms because of over- or underuse of BP lowering treatment. To address these uncertainties, more evidence from randomized trials are needed. However, because single randomized trials are often not large enough for detailed investigation of the safety and efficacy of BP lowering and its variation by class of medication or important patient features, pooling information from several trials remains the most efficient and often the only viable option for addressing these remaining uncertainties.

In 1995, Principal Investigators of major ongoing clinical trials of BP lowering drugs conceived and initiated the Blood Pressure Lowering Treatment Trialists' Collaboration (BPLTTC) to investigate the effect of BP lowering on major cardiovascular events and all-cause mortality through a series of

For peer review only - http://bmjopen.bmj.com/site/about/guidelines.xhtml

individual patient data (IPD) meta-analyses [8]. Since its inception, the BPLTTC has successfully completed several studies which have provided reliable evidence for the benefits of BP lowering for prevention of major cardiovascular outcomes (CHD, heart failure and stroke) and have demonstrated that major classes of anti-hypertensives have similar beneficial effects on major vascular outcomes [9–13]. However, with its limited size and original focus on major cardiovascular outcomes only, the BPLTTC has been unable to address important contemporary questions about the stratified (i.e. subgroup-specific) effects BP lowering and its safety.

Objectives

In 2014, existing BPLTTC investigators were approached and asked to extend the scope of the collaboration with provision of information on all available and accessible baseline, follow-up and outcome data. In addition, investigators from several other large-scale trials that had not participated in BPLTTC or had provided summary data only were invited to join the collaboration with provision of IPD. The key objectives for this extended phase of BPLTTC were specified as follows:

1. To assess the stratified efficacy of BP lowering on major vascular events (CHD, stroke and heart failure) and all-cause mortality by baseline BP levels, sex, age, disease status (including multimorbidity), and risk of CVD at baseline.
2. To assess the overall and stratified safety of BP lowering examining its effect on unintended outcomes, including total serious adverse events, acute renal failure, new-onset diabetes, fractures, and cancer overall and by drug classes and baseline BP levels.
3. To assess the extended efficacy of BP lowering on other important 'vascular' outcomes, including atrial fibrillation and dementia.

Methods and analysis

Inclusion and exclusion criteria:

For this extended phase of the collaboration, the original trial eligibility criteria were modified, chiefly to enable inclusion of additional large-scale trials that were reported before the inception of the BPLTTC in 1995. We will only include randomized clinical trials to minimise the impact of bias and confounding. For clinical trials to be eligible for inclusion in BPLTTC, one of the following criteria must be met:

For peer review only - http://bmjopen.bmj.com/site/about/guidelines.xhtml
For peer review only

- Randomization of patients between a BP lowering agent and a placebo arm (or other inactive control condition)
- Randomization of patients between various BP lowering intensities
- Randomization of patients between various antihypertensive drugs

Additionally, a minimum of 1000 patient-years of follow-up in each randomly allocated trial arm is required. There is no restriction on publication date, setting, or drugs investigated. However, the following types of trial will be excluded:

- Trials exclusively conducted in patients with heart failure or short-term interventions in patients with acute myocardial infarction or other acute settings
- Trials with non-pharmacological interventions of BP lowering without a drug comparison arm
- Trials without a clearly defined randomization process

Identification of trials

We conducted a systematic review to identify trials for possible inclusion in the IPD meta-analysis. The search was restricted to clinical trials, controlled clinical trials, randomized controlled trials or meta-analyses and no language restrictions were applied. We searched electronic bibliographic databases, including PubMed/Medline (NCBI, Bethesda, MD, USA) and The Cochrane Central Register of Controlled Trials (The Cochrane Collaboration, London, UK) covering the periods between 1 Jan 1966 and 1 Jun 2018. Reference lists of eligible studies, related meta-analyses, clinical trial registries, and BPLTTC collected in previous projects [14] were hand-searched to identify further relevant studies. This systematic review protocol, including details of the methods and search strategy, was registered with PROSPERO (www.crd.york.ac.uk/prospero/) (*CRD42018099283*) before the review was conducted. ***Supplementary table 1*** shows the Medline searh strategy. For this protocol, the Preferred Reporting Items for Systematic review and Meta-Analysis Protocols (PRISMA-P) checklist is shown in ***Supplementary Table 2***.

We used reference management software (Endnote) to store identified records from different sources. Duplicate records from various sources were collapsed into a single entry. Two investigators (DC and MN) conducted independent searches and screened the title and abstracts of publications for meeting eligibility criteria. The full manuscript were then requested and assessed

For peer review only

for eligibility. Disagreements were resolved by discussion, with involvement of a third investigator (KR) where necessary.

Because the purpose of this systematic review was to identify potential trials for inclusion in an IPD meta-analysis, we mainly extracted information about the investigators and/or contact details to allow us to invite them to participate by contributing data. When conducting the meta-analysis of RCTs for which we have obtained data at individual patient-level, we will report quality assessment of these RCTs based on randomization procedures, treatment allocations according to assignment, and outcomes collected and compared across comparison groups; concealment and blinding methods; and risk of bias both at study and outcome levels using the Cochrane Risk of Bias Tool. We will use the quality of RCTs as part of sensitivity analysis when conducting the IPD meta-analysis.

Figure 1 summarizes the trial search as of June 2018, indicating that a total of 100 trials with 595,514 randomized participants were identified that potentially met the BPLTTC eligibility criteria (***Supplementary Table 3***). This literature search will be continuously updated, and investigators of newly reported and eligible trials will be invited to join the collaboration.

Data collection

All existing BPLTT collaborators and investigators of all newly identified trials were contacted and asked whether they would be willing to provide IPD (variables are listed in ***Supplementary table 4***). As of June 2018, the collaboration had acquired data from 49 trials comprising 315,046 participants. Investigators or sponsors of several other trials have agreed to share data. Data from another four major trials with 34,642 participants are currently at the transfer stage. For the remaining trials, data are still being sought. Many of these trials have been published many years ago and identification of the data guardian or an electronic database of the trial has proven challenging.

In parallel to requesting further data, the process of data harmonization has commenced. This includes creation of database with all information obtained from investigators, using the data dictionary provided. Initial data verification will include checking of the individual patient data with respect to number of participants, baseline characteristics, and types of follow-up and outcome data available. Next, prepared data are to be checked for consistency (e.g. plausible ranges, randomization sequence etc.) and completeness. In case of any inconsistencies or missing information, the investigators will be contacted for verification.

For peer review only - http://bmjopen.bmj.com/site/about/guidelines.xhtml

For peer review only

Governance and data management policies

Figure 2 provides an overview of roles and responsibilities of the BPLTTC collaborative group. The BPLTTC Director, supported by a core analytic and management group, at the University of Oxford coordinates all activities among internal collaborators and external stakeholders. This central team will periodically update the literature review, acquire new data, develop proposals and protocols, seek approvals and funding, and manage publications and communication. Scientific activities of the BPLTTC core group are overseen by the Steering Committee with input from all BPLTT collaborators.

The aforementioned three main objectives of the extended phase of BPLTTC have all been approved by the Steering Committee and collaborators. *For each of these objectives, at least one study protocol will be developed*. Collaborators or external researchers may suggest additional research proposals to the BPLTTC core group. Such proposals shall be checked for feasibility and overlap with any existing proposals. If appropriate, they will be shared with the Steering Committee for scientific review. After approval, proposing researchers will be able to join a working group, including other interested members of the Collaboration. This working group will work with the BPLTTC core group and Steering Committee to write the research protocol, conduct and interpret the results, draft the manuscript and publish the final report.

Once agreement on a structured protocol is reached by the working group, the study will be registered with PROSPERO and the protocol will be posted on a password protected website at www.bplttc.org. Additionally, collaborators will be able to use this website to stay informed about the progress of various projects.

Trial data from investigators and sponsors are typically transferred by using a secure file transmission system, such as Oxford's Oxfile system. Before sending a data file, the data will be compressed using a standard compression system and then encrypted. All data transfer processes will be overseen by the BPLTTC core group in compliance with the data exchange and sharing agreement with the data provider. All trial data will be considered confidential and will not be provided to any third party without written permission of the owner of the data. Data will be stored on site at the George Institute at the University of Oxford, with strict confidentiality and

For peer review only

For peer review only - http://bmjopen.bmj.com/site/about/guidelines.xhtml

comprehensive data security. All analyses will be carried out by the core analytic team, according to research priorities and availability of resources.

Patient and public involvement

This protocol describes plans for collaborative research investigations involving secondary analyses of existing clinical trials data. There was neither any involvement by patients in conceptualising the study nor in the development of the protocol.

Statistical analysis

Prior to commencing any analysis, a feasibility assessment and power calculation will be carried out on the blinded dataset. For approved proposals, a protocol will be written which will entail a detailed statistical analysis plan. Although specific proposals may require different statistical methods, in principle, we intend to use a one-step approach by simultaneously analysing observations from all trials (while accounting for clustering of observations within studies) [15][16]. This approach is more suitable than a two-step approach when non-linear effects or multiple covariates and their interaction are to be assessed, which our stratified analyses aim to do [17]. For some analyses, it might be important to supplement the available IPD with aggregate data for those studies where IPD are not available, using a combined one-step and two-step approaches. Aggregate data will be obtained by sending our computer codes to the Investigators of such studies, so as to extract their results (e.g. hazard ratios), rather than their data.

We will use fixed effects methods to pool trial-specific data. For time-to-event data clustered Cox proportional-hazard models will be used and hazard ratios with 95% or 99% confidence intervals (CI) will be reported. For binary outcomes, risk ratios and 95% CI will be estimated using log-binomial mixed-effects models (or odds ratios from logistic models, if necessary). Effects on continuous outcomes (e.g. renal function) will be quantified by mean differences and 95% CI and will be estimated using linear mixed effects models.

Using a two-step approach, key results will be presented using Forest plots, and Cochran's Q test and I^2 statistic will be used to quantify the degree of heterogeneity between studies. Meta-regression will be used if considerable residual heterogeneity remains after controlling for all possible variables and recognized effect modifiers, with P-value adjusted for false positive findings based on Monte Carlo simulation.

For peer review only - http://bmjopen.bmj.com/site/about/guidelines.xhtml

Where appropriate, analyses will also be stratified by class of medication, patient population and baseline BP to allow for heterogeneity among different patient populations and types of medication to be determined. In addition, a dose-effect (dose-response) regression model will be applied to assess any potential dose-effect association for each medication dosage and represent efficacy and probable risk of side effects (point-to-point association) both at individual and trial level (aggregate data). Also, we will use fractional polynomials and cubic splines to smoothen the dose-response relationship and address any violated linearity assumption [18]. For analysing drug class-specific effects, we will consider using IPD network meta-analysis [19].

Most trials included in BPLTTC have a double blind design where the use of routine unadjudicated outcomes is unlikely to result in biased estimates (provided sufficient number of events can be gathered) because any under- or over-reporting would affect both arms of the trial equally [20,21]. However, due to the differing data formats of contributing trials and potentially differing types of data provided, additional methodological work might be needed to enhance the quality and power of the data. This might be particularly relevant when a large proportion of data on important covariates, effect modifiers or outcomes is missing. Simply ignoring the missing data, or implementing a complete case analysis, is typically inappropriate, as it could result in biased results. We will therefore conduct methodological studies that will compare existing and novel methods for imputation of non-commensurate measurements between trials, correction of measurement errors, or harmonization of differing definitions among trials [22–25].

In general, reporting of the findings will follow the PRISMA-IPD guidance [26] and where appropriate, we will use Grading of Recommendations Assessment, Development and Evaluation (GRADE) (www.gradeworkinggroup.org) when assessing certainty of evidence.

Ethics and dissemination

As we do not have access to patient identifiable information, our procedures are fully compliant with the Data Protection Act 1998 and consistent with the principles of the General Data Protection Regulation. The University of Oxford's research contracts team is well aware of legal requirements for data sharing and storage and all our procedures are fully compliant with these requirements. These requirements are described in detail in Oxford's research governance (https://researchsupport.admin.ox.ac.uk/governance/integrity) and policies on research data

For peer review only - http://bmjopen.bmj.com/site/about/guidelines.xhtml

handling and management (http://researchdata.ox.ac.uk/). We have already obtained ethics review from the Oxford Central Ethics University Research Committee (OxTREC Reference: 545-14) and agreed to communicate any major amendments to the protocol for this third cycle of BPLTTC with the Ethics Committee for further approval before analyses begin.

The knowledge of the degree of efficacy and safety of BP lowering and its variation by class of medication and among subgroups of patients will help to refine treatment recommendations. The presence of clinically meaningful differences in the efficacy of different classes of BP medication, either overall, or within certain patient groups will have considerable implications for stratified patient management and clinical decision making. BPLTTC is the largest international hypertension collaboration and is unique in its ability to pool and analyse IPD from a large number of trials to answer these clinically relevant questions.

We will publish our findings in peer-reviewed medical journals. Further, we will share and present our findings at various scientific meetings and through the Collaboration's networks and memberships across professional societies. The George Institute for Global Health has communication and dissemination practices in place to ensure that research it produces and policies it contributes to are regularly transmitted through its various institutes and units in Australia, India, China, UK and the US and to its partner organisations globally.

Details about data curation and deposition are described under the section on 'Governance and data management policies'. No data will be provided to any third party without written permission from the trial data provider or custodian. As previously mentioned, the BPLTTC is governed by the University of Oxford's policies on research integrity and codes of practice and follow the university policy on the management of research data and records.

Authors' contributions

Kazem Rahimi wrote the first draft of the manuscript; Kazem Rahimi and Dexter Canoy sought access to the data; Dexter Canoy, Milad LNazarzadeh and Kazem Rahimi conducted the systematic review; Kazem Rahimi, Dexter Canoy, Milad Nazarzadeh, Gholamreza Khorshidi, Mark Woodward, Koon Teo, Barry R. Davis, John Chambers and Carl J. Pepine contributed in the conception and design of the study, provided critical input into the subsequent revisions of the manuscript, and

For peer review only - http://bmjopen.bmj.com/site/about/guidelines.xhtml

gave approval to the final version to be published; Kazem Rahimi and Dexter Canoy are guarantors of this protocol.

Funding statement

BPLTTC is supported by the Oxford Martin School and the National Institute for Health Research (NIHR) Oxford Biomedical Research Centre (BRC).

The views expressed are those of the author(s) and not necessarily those of the NHS, the NIHR or the Department of Health; Funders have no involvement in the conduct of the review or in the development of the protocol.

Competing interests statement

George Health Enterprises, the social enterprise arm of The George Institute for Global Health, has received investment to develop fixed-dose combination products containing aspirin, statin, and blood pressure lowering drugs. George Health Enterprises has submitted patents for low-dose blood pressure combinations on which one of the collaborators (A Rodgers) is listed as one of the inventors, but does not have a financial interest in these planned products.

For peer review only

The Blood Pressure Lowering Treatment Trialists' Collaboration

Steering Committee

Kazem Rahimi (Chair) (The George Institute for Global Health, University of Oxford, Oxford, United Kingdom), Koon Teo (Population Health Research Institute, McMaster University, Hamilton, Ontario, Canada) Barry R Davis (The University of Texas School of Public Health, Houston, Texas, USA), John Chalmers (The George Institute for Global Health, University of New South Wales, Sydney, Australia), Carl J. Pepine (Department of Medicine, University of Florida, Gainesville, Florida, USA)

Collaborating Trialists

L Agodoa (AASK [African-American Study of Kidney Disease and Hypertension]), A Algra (Dutch TIA Study [Dutch Transient Ischemic Attack Study]), F W Asselbergs (PREVEND-IT [Prevention of Renal and Vascular End- stage Disease Intervention Trial]), N Beckett (HYVET [Hypertension in the Very Elderly Trial]), E Berge (VALUE trial [Valsartan Antihypertensive Long-term Use Evaluation trial]), H Black (CONVINCE [Controlled Onset Verapamil Investigation of Cardiovascular End Points]), F.P.J. Brouwers (PREVEND-IT), M Brown (INSIGHT [International Nifedipine GITS Study: Intervention as a Goal in Hypertension]), C J Bulpitt (HYVET), B Byington (PREVENT [Prospective Randomized Evaluation of the Vascular Effects of Norvasc Trial]), J Chalmers (ADVANCE[Action in Diabetes and Vascular Disease: Preterax and Diamicron MR Controlled Evaluation]), J Cutler (ALLHAT [Antihypertensive and Lipid-Lowering Treatment to Prevent Heart Attack Trial]), B Davis (ALLHAT), R B Devereaux (LIFE [Losartan Intervention For Endpoint reduction in hypertension]), D Dwyer (IDNT [Irbesartan Diabetic Nephropathy Trial]), R Estacio (ABCD [Appropriate Blood Pressure Control in Diabetes]), R Fagard (SYST-EUR [SYSTolic Hypertension in EURope]), K Fox (EUROPA [European trial on Reduction Of cardiac events with Perindopril among patients with stable coronary Artery disease]), T Fukui (CASE-J [Candesartan Antihypertensive Survival Evaluation in Japan]), A J Gupta (ASCOT [Anglo-Scandinavian Cardiac Outcomes Trial]), R R Holman (UKPDS [UK Prospective Diabetes Study]), Y Imai (HOMED-BP [Hypertension Objective Treatment Based on Measurement by Electrical Devices of Blood Pressure]), M Ishii (JMIC-B [Japan Multicenter Investigation for Cardiovascular Diseases-B]), S Julius (VALUE), Y Kanno (E-COST [Efficacy of Candesartan on Outcome in Saitama Trial]), S E Kjeldsen (VALUE, LIFE), J Kostis (SHEP [Systolic Hypertension in the Elderly Program]) K Kuramoto (NICS-EH [National Intervention Cooperative Study in Elderly Hypertensives]), J Lanke (STOP2 [Swedish Trial in Old Patients with Hypertension-2], NORDIL [Nordic Diltiazem]), E Lewis (IDNT), J Lewis (IDNT) M Lievre (DIABHYCAR [Non-insulin-dependent diabetes, hypertension, microalbuminuria or proteinuria, cardiovascular events, and ramipril study]), L H Lindholm (CAPPP [Captopril Prevention Project], STOP2, NORDIL), S Lueders (MOSES [The Morbidity and Mortality After Stroke, Eprosartan Compared With Nitrendipine for Secondary Prevention]), S MacMahon (ADVANCE), M Matsuzaki (COPE [The Combination Therapy of Hypertension to Prevent Cardiovascular Events]), M H Mehlum (VALUE), S Nissen (CAMELOT [Comparison of Amlodipine vs Enalapril to Limit Occurrences of Thrombosis]), H Ogawa (HIJ-CREATE [Heart Institute of Japan Candesartan Randomized Trial for Evaluation in Coronary Heart Disease]), T Othisgihara (CASE-J), T Ohkubo (HOMED-BP), C Palmer (INSIGHT), A Patel (ADVANCE), C Pepine (INVEST [International Verapamil SR-Trandolapril Study]), M Pfeffer (PEACE [Prevention of Events With Angiotensin- Converting Enzyme Inhibition]), N R Poulter (ASCOT [Anglo-Scandinavian Cardiac Outcomes Trial]), H Rakugi (VALISH [Valsartan in Elderly Isolated Systolic Hypertension Study], CASE-J), G Remuzzi (BENEDICT [BErgamo NEphrologic DIabetes Complications Trial]), P Ruggenenti (BENEDICT), T Saruta (CASE-J), J Schrader (MOSES), R Schrier (ABCD), P Sever (ASCOT), P Sleight (CONVINCE, HOPE [Heart Outcomes Prevention Evaluation], TRANSCEND [Telmisartan Randomised AssessmeNt Study in ACE iNtolerant subjects with

For peer review only - http://bmjopen.bmj.com/site/about/guidelines.xhtml

cardiovascular Disease], ONTARGET [Ongoing Telmisartan Alone and in Combination with Ramipril Global Endpoint Trial]), J A Staessen (SYST-EUR [Systolic Hypertension in Europe]), H Suzuki (ECOST), L Thijs (Syst-Eur), K Ueshima (VALISH, CASE-J), S Umemoto (COPE), W H van Gilst (PREVEND-IT), P Verdecchia (Cardio-Sis [CARDIOvascolari del Controllo della Pressione Arteriosa SIStolica]), K Wachtell (LIFE), L Wing (ANBP2 [The Second Australian National Blood Pressure Study]), Y Yui (JMIC-B), S Yusuf (HOPE, ONTARGET, TRANSCEND), A Zanchetti (deceased) (VHAS [Verapamil in Hypertension and Atherosclerosis Study], ELSA [European Lacidipine Study on Atherosclerosis]).

Other members
C Baigent, R Collins, D de Zeeuw B Neal, V Perkovic, M Rahman, W J Remme, A Rodgers, J Sundström, F Turnbull.

For peer review only

Figure legends

Figure 1: Literature search and trial selection flowchart.

Figure 2: Overview of collaboration governance.

For peer review only

For peer review only - http://bmjopen.bmj.com/site/about/guidelines.xhtml

References

1 GBD 2016 Risk Factors Collaboration, Abajobir AA, Abate KH, *et al.* Global, regional, and national comparative risk assessment of 84 behavioural, environmental and occupational, and metabolic risks or clusters of risks, 1990–2016: a systematic analysis for the Global Burden of Disease Study 2016. *Lancet* 2017;**390**:1345–422. doi:10.1016/S0140-6736(17)32366-8

2 Mills KT, Bundy JD, Kelly TN, *et al.* Global Disparities of Hypertension Prevalence and Control Clinical Perspective: a Systematic Analysis of Population-Based Studies from 90 Countries. *Circulation* 2016;**134**:441–50. doi:10.1161/CIRCULATIONAHA.115.018912

3 Rahimi K, Emdin CA, MacMahon S. The epidemiology of blood pressure and its worldwide management. *Circ Res* 2015;**116**:925–36. doi:10.1161/CIRCRESAHA.116.304723

4 Emdin CA, Anderson SG, Salimi-Khorshidi G, *et al.* Usual blood pressure, atrial fibrillation and vascular risk: Evidence from 4.3 million adults. *Int J Epidemiol* 2017;**46**:162–72. doi:10.1093/ije/dyw053

5 Emdin CA, Rothwell PM, Salimi-Khorshidi G, *et al.* Blood Pressure and Risk of Vascular Dementia. *Stroke* 2016;**47**:1429–35. doi:10.1161/STROKEAHA.116.012658

6 Emdin CA, Anderson SG, Callender T, *et al.* Usual blood pressure, peripheral arterial disease, and vascular risk: cohort study of 4.2 million adults. *BMJ* 2015;**351**:h4865.

7 Emdin CA, Anderson SG, Woodward M, *et al.* Usual Blood Pressure and Risk of New-Onset Diabetes: Evidence From 4.1 Million Adults and a Meta-Analysis of Prospective Studies. *J Am Coll Cardiol* 2015;**66**:1552–62. doi:10.1016/j.jacc.2015.07.059

8 Protocol for prospective collaborative overviews of major randomized trials of blood-pressure-lowering treatments. World Health Organization-International Society of Hypertension Blood Pressure Lowering Treatment Trialists' Collaboration. *J Hypertens* 1998;**16**:127–37.

9 Blood Pressure Lowering Treatment Trialists' Collaboration, Turnbull F, Neal B, *et al.* Effects of different regimens to lower blood pressure on major cardiovascular events in older and younger adults: meta-analysis of randomised trials. *BMJ* 2008;**336**:1121–3. doi:10.1136/bmj.39548.738368.BE

10 Turnbull F, Blood Pressure Lowering Treatment Trialists' Collaboration. Effects of different blood-pressure-lowering regimens on major cardiovascular events: results of prospectively-designed overviews of randomised trials. *Lancet (London, England)* 2003;**362**:1527–35.

11 Sundström J, Arima H, Jackson R, *et al.* Effects of blood pressure reduction in mild hypertension: a systematic review and meta-analysis. *Ann Intern Med* 2015;**162**:184–91. doi:10.7326/M14-0773

12 Blood Pressure Lowering Treatment Trialists' Collaboration, Ying A, Arima H, *et al.* Effects of blood pressure lowering on cardiovascular risk according to baseline body-mass index: a meta-analysis of randomised trials. *Lancet (London, England)* 2015;**385**:867–74. doi:10.1016/S0140-6736(14)61171-5

13 Turnbull F, Woodward M, Neal B, *et al.* Do men and women respond differently to blood pressure-lowering treatment? Results of prospectively designed overviews of randomized trials. *Eur Heart J* 2008;**29**:2669–80. doi:10.1093/eurheartj/ehn427

14 Ettehad D, Emdin CA, Kiran A, *et al.* Blood pressure lowering for prevention of cardiovascular disease and death: a systematic review and meta-analysis. *Lancet* 2016;**387**:957–67. doi:10.1016/S0140-6736(15)01225-8

15 Hamza TH, van Houwelingen HC, Stijnen T. The binomial distribution of meta-analysis was preferred to model within-study variability. *J Clin Epidemiol* 2008;**61**:41–51. doi:10.1016/j.jclinepi.2007.03.016

For peer review only

For peer review only - http://bmjopen.bmj.com/site/about/guidelines.xhtml

16 Stijnen T, Hamza TH, Özdemir P. Random effects meta-analysis of event outcome in the framework of the generalized linear mixed model with applications in sparse data. *Stat Med* 2010;**29**:3046–67. doi:10.1002/sim.4040

17 Abo-Zaid G, Guo B, Deeks JJ, *et al.* Individual participant data meta-analyses should not ignore clustering. *J Clin Epidemiol* 2013;**66**:865–873.e4. doi:10.1016/j.jclinepi.2012.12.017

18 Bagnardi V, Zambon A, Quatto P, *et al.* Flexible Meta-Regression Functions for Modeling Aggregate Dose-Response Data, with an Application to Alcohol and Mortality. *Am J Epidemiol* 2004;**159**:1077–86. doi:10.1093/aje/kwh142

19 Debray TPA, Schuit E, Efthimiou O, *et al*. An overview of methods for network meta-analysis using individual participant data: when do benefits arise? *Stat Methods Med Res* 2018;**27**:1351-1364.

20 Rahimi K, Bhala N, Kamphuisen P, *et al.* Effect of Statins on Venous Thromboembolic Events: A Meta-analysis of Published and Unpublished Evidence from Randomised Controlled Trials. *PLoS Med* 2012;**9**:e1001310. doi:10.1371/journal.pmed.1001310

21 Pogue J, Walter SD, Yusuf S. Evaluating the benefit of event adjudication of cardiovascular outcomes in large simple RCTs. *Clin Trials* 2009;**6**:239–51. doi:10.1177/1740774509105223

22 Resche-Rigon M, White IR, Bartlett JW, *et al.* Multiple imputation for handling systematically missing confounders in meta-analysis of individual participant data. *Stat Med* 2013;**32**:4890–905. doi:10.1002/sim.5894

23 Siddique J, Reiter JP, Brincks A, *et al.* Multiple imputation for harmonizing longitudinal non-commensurate measures in individual participant data meta-analysis. *Stat Med* 2015;**34**:3399–414. doi:10.1002/sim.6562

24 Kunkel D, Kaizar EE. A comparison of existing methods for multiple imputation in individual participant data meta-analysis. *Stat Med* 2017;**36**:3507–32. doi:10.1002/sim.7388

25 Schifeling T, Reiter JP, DeYoreo M. Data Fusion for Correcting Measurement Errors. 2016;:1–31.

26 Stewart LA, Clarke M, Rovers M, *et al.* Preferred Reporting Items for a Systematic Review and Meta-analysis of Individual Participant Data. *JAMA* 2015;**313**:1657. doi:10.1001/jama.2015.3656

For peer review only

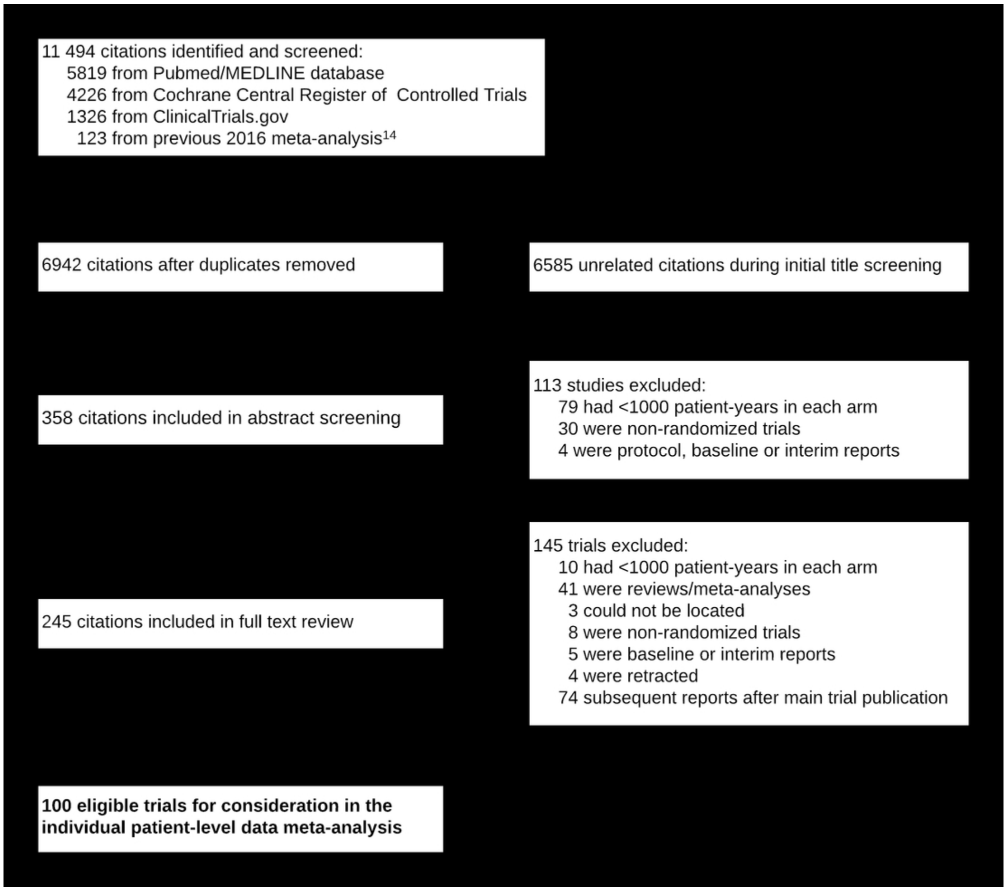

Figure 1: Literature search and trial selection flowchart

93x82mm (300 x 300 DPI)

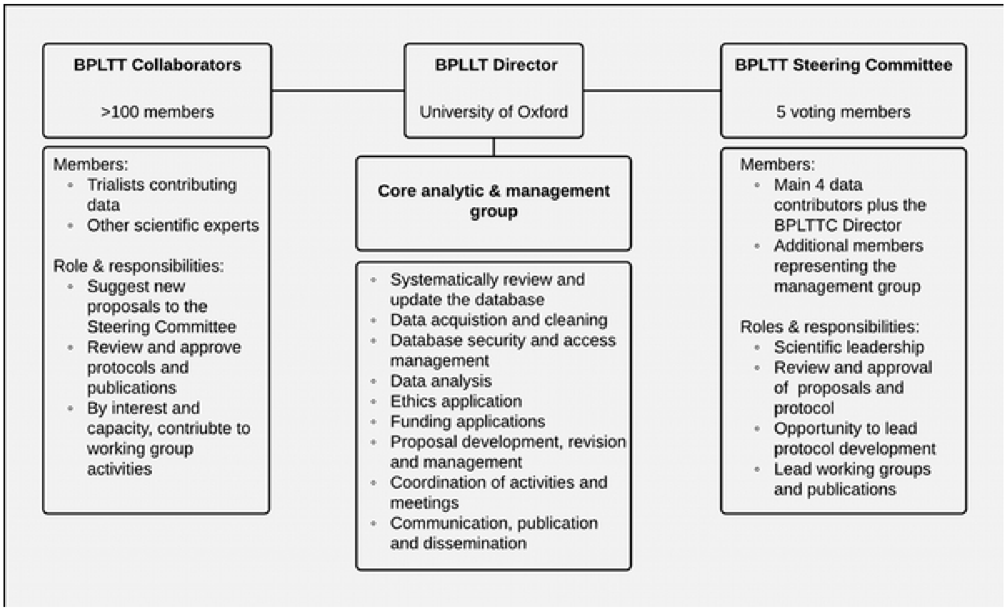

Figure 2: Overview of collaboration governance.

50x30mm (300 x 300 DPI)

SUPPLEMENTARY MATERIAL

Investigating the stratified efficacy and safety of pharmacological blood pressure lowering: An overall protocol for individual patient-level data meta-analyses of over 300,000 randomized participants in the new phase of the Blood Pressure Lowering Treatment Trialists' Collaboration (BPLTTC)

Kazem Rahimi,[1] Dexter Canoy,[1,2] Milad Nazarzadeh,[1,3] Gholamreza Khorshidi,[1] Mark Woodward,[1,4] Koon Teo,[5] Barry R. Davis,[6] John Chalmers,[4] Carl J. Pepine,[7] on behalf of the Blood Pressure Lowering Treatment Trialists' Collaboration

[1]The George Institute for Global Health UK, University of Oxford, Oxford, UK

[2] Faculty of Medicine, University of New South Wales, Sydney, Australia

[3] Collaboration Center of Meta-Analysis Research, Torbat Heydariyeh University of Medical Sciences, Torbat Heydariyeh, Iran

[4]The George Institute for Global Health, University of New South Wales, Sydney, Australia

[5]Population Health Research Institute, McMaster University, Hamilton, Ontario, Canada

[6]The University of Texas School of Public Health, Houston, Texas, USA

[7]Department of Medicine, University of Florida, Gainesville, Florida, USA

E-mail address:

kazem.rahimi@georgeinstitute.ox.ac.uk (KR)

dexter.canoy@georgeinstitute.ox.ac.uk (DC)

milad.nazarzadehlarzjan@georgeinstitute.ox.ac.uk (MN)

reza.khorshidi@georgeinstitute.ox.ac.uk (GK)

mark.woodward@georgeinstitute.ox.ac.uk (MW)

Koon.Teo@phri.ca (KT)

Barry.R.Davis@uth.tmc.edu (BRD)

chalmers@georgeinstitute.org.au (JC)

Carl.Pepine@medicine.ufl.edu (CP)

For peer review only

For peer review only - http://bmjopen.bmj.com/site/about/guidelines.xhtml

Supplementary table 1. Search strategy to be used in MEDLINE, covering indexed entries between 01 Jan 1966 to 01 Jun 2018.

Search (((((("Hypertension/drug effects"[Mesh] OR "Hypertension/drug therapy"[Mesh]))) AND (("Blood Pressure/drug effects"[Mesh] OR "Blood Pressure/therapy"[Mesh]))) AND ((Clinical Trial[ptyp] OR Controlled Clinical Trial[ptyp] OR Meta-Analysis[ptyp] OR Randomized Controlled Trial[ptyp] OR Clinical Trial, Phase III[ptyp]) AND Humans[Mesh] AND adult[MeSH]))) AND (((((((((("Antihypertensive Agents" [Pharmacological Action]) OR "Antihypertensive Agents/therapeutic use"[Mesh])) OR ((("Vasodilator Agents" [Pharmacological Action])) OR ("Vasodilator Agents/therapeutic use"[Mesh] OR "Vasodilator Agents/therapy"[Mesh]))) OR (("Adrenergic alpha-Antagonists/therapeutic use"[Mesh]) OR "Adrenergic alpha-Antagonists" [Pharmacological Action])) OR (("Adrenergic beta-Antagonists" [Pharmacological Action]) OR "Adrenergic beta-Antagonists/therapeutic use"[Mesh])) OR (("Sodium Chloride Symporter Inhibitors" [Pharmacological Action]) OR "Sodium Chloride Symporter Inhibitors/therapeutic use"[Mesh])) OR "Angiotensin-Converting Enzyme Inhibitors/therapeutic use"[Mesh]) OR (("Angiotensin II Type 1 Receptor Blockers" [Pharmacological Action]) OR "Angiotensin II Type 1 Receptor Blockers/therapeutic use"[Mesh])) OR (("Calcium Channel Blockers" [Pharmacological Action]) OR "Calcium Channel Blockers/therapeutic use"[Mesh])) AND ((Clinical Trial[ptyp] OR Controlled Clinical Trial[ptyp] OR Meta-Analysis[ptyp] OR Randomized Controlled Trial[ptyp] OR Clinical Trial, Phase III[ptyp]) AND Humans[Mesh] AND adult[MeSH])) Filters: Clinical Trial; Controlled Clinical Trial; Randomized Controlled Trial; Clinical Trial, Phase III; Meta-Analysis; Systematic Reviews; Humans; Adult: 19+ years

For peer review only

Supplementary table 2. PRISMA-P (Preferred Reporting Items for Systematic review and Meta-Analysis Protocols) checklist for the Blood Pressure Lowering Treatment Trialists' Collaboration protocol for individual patient-level data meta-analysis

Section and topic	Item No	Checklist item	Location in the manuscript
**ADMINISTRATIVE INFORMATION**			
Title:			
Identification	1a	Identify the report as a protocol of a systematic review	Title page
Update	1b	If the protocol is for an update of a previous systematic review, identify as such	NA
Registration	2	If registered, provide the name of the registry (such as PROSPERO) and registration number	Page 5
Authors:			
Contact	3a	Provide name, institutional affiliation, e-mail address of all protocol authors; provide physical mailing address of corresponding author	Title page (email address in Supplementary material title page)
Contributions	3b	Describe contributions of protocol authors and identify the guarantor of the review	Page 10
Amendments	4	If the protocol represents an amendment of a previously completed or published protocol, identify as such and list changes; otherwise, state plan for documenting important protocol amendments	NA
Support:			
Sources	5a	Indicate sources of financial or other support for the review	Page 10
Sponsor	5b	Provide name for the review funder and/or sponsor	Page 10
Role of sponsor or funder	5c	Describe roles of funder(s), sponsor(s), and/or institution(s), if any, in developing the protocol	Page 10
**INTRODUCTION**			
Rationale	6	Describe the rationale for the review in the context of what is already known	Page 3
Objectives	7	Provide an explicit statement of the question(s) the review will address with reference to participants, interventions, comparators, and outcomes (PICO)	Page 4
**METHODS**			
Eligibility criteria	8	Specify the study characteristics (such as PICO, study design, setting, time frame) and report characteristics (such as years considered, language, publication status) to be used as criteria for eligibility for the review	Pages 4 to 5
Information sources	9	Describe all intended information sources (such as electronic databases, contact with study authors, trial registers or other grey literature sources) with planned dates of coverage	Page 5

For peer review only - http://bmjopen.bmj.com/site/about/guidelines.xhtml

Search strategy	10	Present draft of search strategy to be used for at least one electronic database, including planned limits, such that it could be repeated	Supplementary table 1
Study records:			
Data management	11a	Describe the mechanism(s) that will be used to manage records and data throughout the review	Page 5
Selection process	11b	State the process that will be used for selecting studies (such as two independent reviewers) through each phase of the review (that is, screening, eligibility and inclusion in meta-analysis)	Pages 5 to 6
Data collection process	11c	Describe planned method of extracting data from reports (such as piloting forms, done independently, in duplicate), any processes for obtaining and confirming data from investigators	Page 6
Data items	12	List and define all variables for which data will be sought (such as PICO items, funding sources), any pre-planned data assumptions and simplifications	Supplementary table 3 (Protocols for each meta-analysis will be developed and published which will include specific definitions of relevant variables)
Outcomes and prioritization	13	List and define all outcomes for which data will be sought, including prioritization of main and additional outcomes, with rationale	For each meta-analysiss (to address each specific objectives), specific protocol will be developed and published which will include a definition of the outcome of interest
Risk of bias in individual studies	14	Describe anticipated methods for assessing risk of bias of individual studies, including whether this will be done at the outcome or study level, or both; state how this information will be used in data synthesis	Page 6
Data synthesis	15a	Describe criteria under which study data will be quantitatively synthesised	Pages 8 to 9
	15b	If data are appropriate for quantitative synthesis, describe planned summary measures, methods of handling data and methods of combining data from studies, including any planned exploration of consistency (such as I^2, Kendall's τ)	Pages 8 to 9 (more detailed analytical plans will be described in separate protocols for each meta-analysis)
	15c	Describe any proposed additional analyses (such as sensitivity or subgroup analyses, meta-regression)	Pages 8 to 9
	15d	If quantitative synthesis is not appropriate, describe the type of summary planned	NA
Meta-bias(es)	16	Specify any planned assessment of meta-bias(es) (such as publication bias across studies, selective reporting within studies)	Pages 6 and 9 (specific assessments will be described in the protocol for each planned meta-analysis)
Confidence in cumulative evidence	17	Describe how the strength of the body of evidence will be assessed (such as GRADE)	Page 9

Reference: Shamseer L, Moher D, Clarke M, Ghersi D, Liberati A, Petticrew M, Shekelle P, Stewart L, PRISMA-P Group. Preferred reporting items for systematic review and meta-analysis protocols (PRISMA-P) 2015: elaboration and explanation. BMJ. 2015;349:g7647.

For peer review only

For peer review only - http://bmjopen.bmj.com/site/about/guidelines.xhtml

Supplementary table 3. Blood pressure lowering randomized trials eligible for BPLTTC individual patient-level data meta-analysis.

Study name or author	Publication year	Total N	Intervention/Comparisons
**AASK** (African American Study of Kidney Disease and Hypertension)[1]	2006	1094	Intensive BP lowering, moderate BP lowering, ACEi, CCB and BB (factorial design)
**ABCD** (Appropriate Blood Pressure Control in Diabetes Trial)[2]	1998	1900	BP lowering to different targets
**ACCOMPLISH** (Avoiding Cardiovascular events through Combination therapy in Patients Living with Systolic Hypertension)[3]	2008	11,506	CCB + ACEi vs diuretic
**ACCORD** (Action to Control Cardiovascular Risk in Diabetes)[4]	2010	4733	Intensive vs standard BP lowering
**ACTION** (A Coronary Disease Trial Investigating Outcome with Nifedipine GITS)[5]	2004	7665	CCB vs placebo
**ACTIVE I** (Atrial Fibrillation Clopidogrel Trial with Irbesartan for Prevention of Vascular Events)[6]	2011	9016	AARB vs placebo
**ADVANCE** (Action in Diabetes and Vascular Disease)[7]	2007	11,140	ACEi + diuretic vs placebo
**ALLHAT** (Antihypertensive and Lipid Lowering Treatment to Prevent Heart Attacks Trial)[8]	2002	42,418	Diuretic vs CCB vs ACEi vs alpha-adrenergic blocker
**ALTITUDE** (Aliskiren Trial in Type 2 Diabetes Using Cardiovascular and Renal Disease Endpoints)[9]	2012	8561	Renin inhibitor vs placebo
**ANBP** (The Australian National Blood Pressure Study)[10]	1980	3427	Diuretic ± alpha-adrenergic agonist/BB vs placebo
**ANBP2** (Second Australian National Blood Pressure Study)[11]	2003	6083	ACEi vs diuretic
**APSIS** (Angina Prognosis Study in Stockholm)[12]	1996	809	BB vs CCB
**ASCOT-BPLA** (Anglo-Scandinavian Cardiac Outcomes Trial - Blood Pressure Lowering Arm)[13]	2005	19,257	BB vs CCB
**ATTEMPT-CVD** (A Trial of Telmisartan Prevention of Cardiovascular Diseases)[14]	2016	1228	ARB vs non-ARB
**BBB** (Behandla Blodtryck Battre)[15]	1994	2127	Intensive vs usual BP lowering
**BCAPS** (β-Blocker Cholesterol-Lowering Asymptomatic Plaque Study)[16]	2001	793	BB vs placebo
**BENEDICT** (Bergamo Nephrologic Diabetes Complications Trial)[17]	2004	1808	ACEi, CCB or combination vs placebo; ACEi vs CCB
**CAMELOT** (The Comparison of Amlodipine vs Enalapril to Limit Occurrences of Thrombosis)[18]	2004	3327	CCB or ACEi vs placebo; CCB vs ACEi
**CAPPP** (Captopril Prevention Project)[19]	1999	10,985	ACEi vs diuretic + BB
**Cardio-Sis** (CARDIOvascolari del Controllo della Pressione Arteriosa SIStolica)[20]	2009	1111	Tight vs usual BP lowering
**CASE-J** (Candesartan Antihypertensive Survival Evaluation in Japan)[21]	2008	4703	ARB vs CCB
**CHIEF** (Chinese Hypertension Intervention Efficacy study)[22]	2012	13,080	CCB + diuretic vs CCB + ARB
**COLM** (Combination of OLMesartan and calcium channel blocker or diuretic)[23]	2014	5141	CCB + ARB vs diuretic + ARB
**CONVINCE** (Controlled Onset Verapamil Investigation of Cardiovascular End Points)[24]	2003	16,476	CCB vs BB or diuretic
**Coope J**, et al[25]	1986	884	BB ± diuretic
**COPE** (Combination Therapy of Hypertension to Prevent Cardiovascular Events)[26]	2011	6586	BB + CCB vs ARB + CCB; ARB + CCB vs diuretic + CCB; BB + CCB vs diuretic + CCB
**DIABHYCAR** (Non-insulin-dependent diabetes, hypertension, microalbuminuria or proteinuria, cardiovascular events, and ramipril)[27]	2004	4912	ACEi vs placebo
**DIME** (Diuretics in the Management of Essential hypertension study)[28]	2014	1130	Diuretic vs non-diuretic
**DIRECT-Prevent 1** (Effect of candesartan on prevention)[29]	2008	1421	ARB vs placebo
**DIRECT-Protect 1** (Effect of candesartan on progression)[29]	2008	1905	ARB vs placebo
**DIRECT-Protect 2** (Effect of candesartan on progression and regression of retinopathy in type 2 diabetes)[30]	2011	1905	ARB vs placebo
**DREAM** (Diabetes REduction Assessment with ramipril and rosiglitazone Medication)[31]	2006	5269	ACEi vs placebo
**Dutch TIA Trial** (Dutch Transient Ischemic Attack Trial)[32]	1993	1473	BB vs placebo

For peer review only - http://bmjopen.bmj.com/site/about/guidelines.xhtml

**E-COST** (Efficacy of Candesartan on Outcome in Saitama Trial)[33]	2005	2048	ARB vs conventional drug (other than ACEi or ARB)
ELSA (Efficacy of Candesartan on Outcome in Saitama Trial)[34]	2002	2334	BB vs CCB
**EUROPA** (European trial on reduction of cardiac events with perindopril in stable coronary artery)[35]	2003	12,218	ACEi vs placebo
**EWPHE** (European Working Party on High Blood Pressure in the Elderly)[36]	1985	840	Diuretic vs placebo
**FEVER** (Felodipine Event Reduction Study)[37]	2005	9711	CCB + diuretic vs diuretic
**HAPPHY** (Heart Attack Primary Prevention in Hypertension Trial)[38]	1987	6569	Diuretic vs BB
**HDFP** (Hypertension Detection and Follow Up Program)[39]	1979	10,940	Intensive (stepped care) vs usual (referred care) BP lowering
**HIJ-CREATE** (Heart Institute of Japan Candesartan Randomized Trial for Evaluation in Coronary Heart Disease)[40]	2009	2049	ARB vs non-ARB
**HOMED-BP** (Hypertension Objective Treatment based on Measurement by Electrical Devices of Blood Pressure Study)[41]	2012	3518	Tight BP control, usual BP control, ACEi, CCB and ARB (factorial design)
**HOPE** (Heart Outcomes Prevention Evaluation Study)[42]	2000	9297	ACEi vs placebo
**HOPE-3** (Heart Outcomes Prevention Evaluation-3)[43]	2016	12,705	ARB + diuretic vs placebo
**HOT** (Hypertension Optimal Treatment Study)[44]	1998	18,790	DBP lowering to ≤80, ≤85 and ≤90 mmHg
**HYVET** (Hypertension in the Very Elderly Trial)[45]	2008	3845	Diuretic vs placebo
**IDNT** (Irbesartan Diabetic Nephropathy Trial)[46]	2001	2861	ARB or CCB vs placebo; ARB vs CCB
**INSIGHT** (International Nifedipine GITS Study: Intervention as a Goal for Hypertension Therapy)[47]	2000	6321	CCB vs diuretic
**INVEST** (International Verapamil-Trandolapril Study)[48]	2003	22,576	CCB vs BB
**IPPSH** (International Prospective Primary Prevention Study in Hypertension)[49]	1985	6357	BB vs non-BB
**JATOS** (Japanese Trial to Assess Optimal Systolic Blood Pressure in Elderly Hypertensive Patients)[50]	2008	4418	Strict vs mild BP lowering
**JMIC-B** (Japan Multicenter Investigation for Cardiovascular Diseases-B)[51]	2004	1650	CCB vs ACEi
**LIFE** (Losartan Intervention for Endpoint Reduction in Hypertension Study)[52]	2002	9193	ARB vs BB
**LIT** (Lopressor Intervention Trial)[53]	1987	2395	BB vs placebo
**MIDAS** (Multicenter Isradipine Diuretic Atherosclerosis Study)[54]	1996	883	CCB vs diuretic
**MOSES** (Morbidity and Mortality After Stroke, Eprosartan Compared With Nitrendipine for Secondary Prevention)[55]	2005	1352	ARB vs CCB
**MRC-1** (Medical Research Council Treatment of Mild Hypertension)[56]	1985	26,054	Diuretic or BB vs placebo
**MRC-2** (Medical Research Council Treatment of Hypertension in Older Adults)[57]	1992	6579	Diuretic or BB vs placebo; Diuretic vs BB
**Multicentre International Study**[58]	1975	3038	BB vs placebo
**NAVIGATOR** (Nateglinide and Valsartan in Impaired Glucose Tolerance Outcomes Research)[59]	2010	9306	ARB vs placebo
**NICOLE** (Nisoldipine in Coronary Artery Disease in Leuven Study)[60]	2001	819	CCB vs placebo
**NICS-EH** (National Intervention Cooperative Study in Elderly Hypertensives)[61]	1999	414	CCB vs diuretic
**NORDIL** (Nordic Diltiazem Study)[62]	2000	10,881	CCB vs diuretic
**ONTARGET** (Ongoing Telmisartan Alone and in Combination with Ramipril Global Endpoint Trial)[63]	2008	34,196	ARB + ACEi vs ACEi; ARB vs ACEi
**OSCAR** (OlmeSartan and calcium antagonists randomized study)[64]	2012	1164	ARB vs ARB + CCB
**The Oslo Study**[65]	1980	785	Diuretic ± alpha-adrenergic agonist or BB vs placebo
**PART-2** (Prevention of Atherosclerosis with Ramipril Trial)[66]	2000	617	ACEi vs placebo
**PATE-Hypertension** (The Practitioner's Trial on the Efficacy of Antihypertensive Treatment in the Elderly with Hypertension)[67]	2000	1748	ACEi vs CCB
**PATS** (Post-stroke Antihypertensive Treatment Study)[68]	2009	5665	Diuretic vs placebo

For peer review only

**PEACE** (Prevention of Events with Angiotensin Converting Enzyme Inhibition)[69]	2004	8290	ACEi vs placebo
**PHARAO** (Prevention of hypertension with the angiotensin-converting enzyme inhibitor ramipril in patients with high-normal blood pressure)[70]	2008	1008	ACEi vs placebo
**PREVEND IT** (Prevention of Renal and Vascular Endstage Disease)[71]	2004	864	ACEi vs placebo
**PREVENT** (Prospective Randomized Evaluation of the Vascular Effects of Norvasc Trial)[72]	2000	825	CCB vs placebo
**PRoFESS** (Prevention Regimen For Effectively Avoiding Second Strokes)[73]	2008	20,332	ARB vs placebo
**PROGRESS** (Perindopril Protection Against Recurrent Stroke Study)[74]	2001	6105	ACEi ± diuretic vs placebo
**QUIET** (Quinapril Ischaemic Event Trial)[75]	2001	1750	ACEi vs placebo
**RENAAL** (Reduction of Endpoints in NIDDM with the Angiotensin II Antagonist Losartan Study)[76]	2001	1513	ARB vs placebo
**ROADMAP** (Randomized Olmesartan And Diabetes Microalbuminuria Prevention Study)[77]	2011	4447	ARB vs placebo
**SCOPE** (Study on Cognition and Prognosis in the Elderly)[78]	2003	4937	ARB vs placebo
**SHELL** (Systolic Hypertension in the Elderly Long-term Lacidipine Trial)[79]	2003	1882	CCB vs diuretic
**SHEP** (Systolic Hypertension in the Elderly Program)[80]	1991	4736	Diuretic ± BB vs placebo
**SPRINT** (Systolic Blood Pressure Intervention Trial)[81]	2015	9361	Intensive vs standard BP lowering
**SPS3** (Secondary Prevention of Small Subcortical Strokes)[82]	2013	3020	SBP lowering to <120 and <140 mmHg
**STONE** (Shanghai trial of nifedipine in the elderly)[83]	1996	1632	CCB vs placebo
**STOP Hypertension** (Swedish Trial in Old Patients with Hypertension)[84]	1991	1627	BB + diuretic vs placebo
**STOP Hypertension-2** (Swedish Trial in Old Patients with Hypertension-2)[85]	1999	13,228	ACEi vs conventional drug; CCB vs conventional drug; ACEi vs CCB
**Sun M**, et al[86]	1997	2080	CCB vs placebo
**Syst-China** (Systolic Hypertension in China)[87]	1988	2394	CCB ± ACEi and/or diuretic vs placebo
**Syst-Eur** (Systolic Hypertension in Europe)[88]	1997	4695	CCB ± ACEi and/or diuretic
**Taylor SH**, et al[89]	1982	1103	BB vs placebo
**TEST** (Ternormin after stroke and TIA)[90]	1995	720	BB vs placebo
**TOMHS** (Treatment of Mild Hypertension Study)[91]	1993	902	Multi-drug class combination vs placebo
**TRANSCEND** (Telmisartan Randomised Assessment Study in ACE Intolerant Subjects with Cardiovascular Disease)[92]	2008	5926	ARB vs placebo
**TROPHY** (Trial of Preventing Hypertension)[93]	2006	772	ARB vs placebo
**UKPDS** (UK Prospective Diabetes Study)[94]	1998	1906	Strict vs less strict BP lowering; ACEi vs BB
**VA NEPHRON-D** (Veterans Affairs Nephropathy in Diabetes)[95]	2013	1448	ACEi + ARB vs ARB
**VALISH** (Valsartan in Elderly Isolated Systolic Hypertension Study)[96]	2010	3079	Strict vs moderate BP lowering
**VALUE** (Valsartan Antihypertensive Long-Term Use Evaluation)[97]	2004	15,245	ARB vs CCB
**VHAS** (Verapamil in Hypertension and Atherosclerosis Study)[98]	1997	1414	Diuretic vs CCB
**Wei Y**, et al.[99]	2013	724	Intensive vs standard BP lowering

BPLTTC – Blood Pressure Lowering Treatment Trialists' Collaboration; ACEi – Angiotensin-converting enzyme inhibitor; ARB – Angiotensin receptor blocker; BB – Beta-blocker; CCB – Calcium channel blocker; BP – blood pressure; SBP – Systolic BP; DBP – Diastolic BP.

For peer review only

For peer review only - http://bmjopen.bmj.com/site/about/guidelines.xhtml

Supplementary table 3 references:

1. Wright JT, Jr., Bakris G, Greene T, et al. Effect of blood pressure lowering and antihypertensive drug class on progression of hypertensive kidney disease: results from the AASK trial. *JAMA* 2002;**288**:2421-31.
2. Estacio RO, Jeffers BW, Hiatt WR, et al. The effect of nisoldipine as compared with enalapril on cardiovascular outcomes in patients with non-insulin-dependent diabetes and hypertension. *New Engl J Med* 1998;**338**:645-52. doi:10.1056/NEJM199803053381003
3. Jamerson K, Weber MA, Bakris GL, et al. Benazepril plus amlodipine or hydrochlorothiazide for hypertension in high-risk patients. *New Engl J Med* 2008;**359**:2417-28. doi:10.1056/NEJMoa0806182
4. ACCORD Study Group, Cushman WC, Evans GW, et al. Effects of intensive blood-pressure control in type 2 diabetes mellitus. *New Engl J Med* 2010;**362**:1575-85. doi:10.1056/NEJMoa1001286
5. Poole-Wilson PA, Lubsen J, Kirwan BA, et al. Effect of long-acting nifedipine on mortality and cardiovascular morbidity in patients with stable angina requiring treatment (ACTION trial): randomised controlled trial. *Lancet* 2004;**364**:849-57. doi:10.1016/S0140-6736(04)16980-8
6. Active I Investigators, Yusuf S, Healey JS, et al. Irbesartan in patients with atrial fibrillation. *New Engl J Med* 2011;**364**:928-38. doi:10.1056/NEJMoa1008816
7. Patel A, Group AC, MacMahon S, et al. Effects of a fixed combination of perindopril and indapamide on macrovascular and microvascular outcomes in patients with type 2 diabetes mellitus (the ADVANCE trial): a randomised controlled trial. *Lancet* 2007;**370**:829-40. doi:10.1016/S0140-6736(07)61303-8
8. Major cardiovascular events in hypertensive patients randomized to doxazosin vs chlorthalidone: the antihypertensive and lipid-lowering treatment to prevent heart attack trial (ALLHAT). ALLHAT Collaborative Research Group. *JAMA* 2000;**283**:1967-75.
9. Parving HH, Brenner BM, McMurray JJ, et al. Cardiorenal end points in a trial of aliskiren for type 2 diabetes. *New Engl J Med* 2012;**367**:2204-13. doi:10.1056/NEJMoa1208799
10. The Australian therapeutic trial in mild hypertension. Report by the Management Committee. *Lancet* 1980;**315**:1261-7.
11. Wing LM, Reid CM, Ryan P, et al. A comparison of outcomes with angiotensin-converting--enzyme inhibitors and diuretics for hypertension in the elderly. *New Engl J Med* 2003;**348**:583-92. doi:10.1056/NEJMoa021716
12. Rehnqvist N, Hjemdahl P, Billing E, et al. Effects of metoprolol vs verapamil in patients with stable angina pectoris. The Angina Prognosis Study in Stockholm (APSIS). *Eur Heart J* 1996;**17**:76-81.
13. Dahlöf B, Sever PS, Poulter NR, et al. Prevention of cardiovascular events with an antihypertensive regimen of amlodipine adding perindopril as required versus atenolol adding bendroflumethiazide as required, in the Anglo-Scandinavian Cardiac Outcomes Trial-Blood Pressure Lowering Arm (ASCOT-BPLA): a multicentre randomised controlled trial. *Lancet* 2005;**366**:895-906. doi:10.1016/s0140-6736(05)67185-1
14. Ogawa H, Soejima H, Matsui K, et al. A trial of telmisartan prevention of cardiovascular diseases (ATTEMPT-CVD): Biomarker study. *Eur J Prev Cardiol* 2016;**23**:913-21. doi:10.1177/2047487315603221
15. Hansson L. The BBB Study: the effect of intensified antihypertensive treatment on the level of blood pressure, side-effects, morbidity and mortality in well-treated hypertensive patients. Behandla Blodtryck Battre. *Blood Pressure* 1994;**3**:248--54.
16. Hedblad B, Wikstrand J, Janzon L, et al. Low-dose metoprolol CR/XL and fluvastatin slow progression of carotid intima-media thickness: Main results from the Beta-Blocker Cholesterol-Lowering Asymptomatic Plaque Study (BCAPS). *Circulation* 2001;**103**:1721-6.
17. Ruggenenti P, Fassi A, Ilieva AP, et al. Preventing microalbuminuria in type 2 diabetes. *New Engl J Med* 2004;**351**:1941-51. doi:10.1056/NEJMoa042167
18. Nissen SE, Tuzcu EM, Libby P, et al. Effect of antihypertensive agents on cardiovascular events in patients with coronary disease and normal blood pressure: the CAMELOT study: a randomized controlled trial. *JAMA* 2004;**292**:2217-25. doi:10.1001/jama.292.18.2217
19. Hansson L, Lindholm LH, Niskanen L, et al. Effect of angiotensin-converting-enzyme inhibition compared with conventional therapy on cardiovascular morbidity and mortality in hypertension: the Captopril Prevention Project (CAPPP) randomised trial. *Lancet* 1999;**353**:611-6.
20. Verdecchia P, Staessen JA, Angeli F, et al. Usual versus tight control of systolic blood pressure in non-diabetic patients with hypertension (Cardio-Sis): an open-label randomised trial. *Lancet* 2009;**374**:525-33. doi:10.1016/S0140-6736(09)61340-4
21. Ogihara T, Nakao K, Fukui T, et al. Effects of candesartan compared with amlodipine in hypertensive patients with high cardiovascular risks: candesartan antihypertensive survival evaluation in Japan trial. *Hypertension* 2008;**51**:393-8. doi:10.1161/HYPERTENSIONAHA.107.098475
22. Ma L, Wang W, Zhao Y, et al. Combination of amlodipine plus angiotensin receptor blocker or diuretics in high-risk hypertensive patients: a 96-week efficacy and safety study. *Am J Cardiovasc Drugs* 2012;**12**:137-42. doi:10.2165/11598110-000000000-00000

For peer review only

23. Ogihara T, Saruta T, Rakugi H, et al. Combinations of olmesartan and a calcium channel blocker or a diuretic in elderly hypertensive patients: a randomized, controlled trial. *J Hypertens* 2014;**32**:2054-63; discussion 63. doi:10.1097/HJH.0000000000000281
24. Black HR, Elliott WJ, Grandits G, et al. Principal results of the Controlled Onset Verapamil Investigation of Cardiovascular End Points (CONVINCE) trial. *JAMA* 2003;**289**:2073-82. doi:10.1001/jama.289.16.2073
25. Coope J, Warrender TS. Randomised trial of treatment of hypertension in elderly patients in primary care. *BMJ* 1986;**293**:1145-51.
26. Matsuzaki M, Ogihara T, Umemoto S, et al. Prevention of cardiovascular events with calcium channel blocker-based combination therapies in patients with hypertension: a randomized controlled trial. *J Hypertens* 2011;**29**:1649-59. doi:10.1097/HJH.0b013e328348345d
27. Marre M, Lievre M, Chatellier G, et al. Effects of low dose ramipril on cardiovascular and renal outcomes in patients with type 2 diabetes and raised excretion of urinary albumin: randomised, double blind, placebo controlled trial (the DIABHYCAR study). *BMJ* 2004;**328**:495. doi:10.1136/bmj.37970.629537.0D
28. Ueda S, Morimoto T, Ando S, et al. A randomised controlled trial for the evaluation of risk for type 2 diabetes in hypertensive patients receiving thiazide diuretics: Diuretics In the Management of Essential hypertension (DIME) study. *BMJ Open* 2014;**4**:e004576. doi:10.1136/bmjopen-2013-004576
29. Chaturvedi N, Porta M, Klein R, et al. Effect of candesartan on prevention (DIRECT-Prevent 1) and progression (DIRECT-Protect 1) of retinopathy in type 1 diabetes: randomised, placebo-controlled trials. *Lancet* 2008;**372**:1394-402. doi:10.1016/S0140-6736(08)61412-9
30. Tillin T, Orchard T, Malm A, et al. The role of antihypertensive therapy in reducing vascular complications of type 2 diabetes. Findings from the DIabetic REtinopathy Candesartan Trials-Protect 2 study. *J Hypertens* 2011;**29**:1457-62. doi:10.1097/HJH.0b013e3283480db9
31. DREAM Trial Investigators, Bosch J, Yusuf S, et al. Effect of ramipril on the incidence of diabetes. *New Engl J Med* 2006;**355**:1551-62. doi:10.1056/NEJMoa065061
32. Trial of secondary prevention with atenolol after transient ischemic attack or nondisabling ischemic stroke. The Dutch TIA Trial Study Group. *Stroke* 1993;**24**:543-8.
33. Suzuki H, Kanno Y, Group EoCoOiST. Effects of candesartan on cardiovascular outcomes in Japanese hypertensive patients. *Hypertens Res* 2005;**28**:307-14. doi:10.1291/hypres.28.307
34. Zanchetti A, Bond MG, Hennig M, et al. Calcium antagonist lacidipine slows down progression of asymptomatic carotid atherosclerosis: principal results of the European Lacidipine Study on Atherosclerosis (ELSA), a randomized, double-blind, long-term trial. *Circulation* 2002;**106**:2422-7.
35. Fox KM, Investigators EtOrocewPiscAd. Efficacy of perindopril in reduction of cardiovascular events among patients with stable coronary artery disease: randomised, double-blind, placebo-controlled, multicentre trial (the EUROPA study). *Lancet* 2003;**362**:782-8.
36. Amery A, Birkenhager W, Brixko P, et al. Mortality and morbidity results from the European Working Party on High Blood Pressure in the Elderly trial. *Lancet* 1985;**1**:1349-54.
37. Liu L, Zhang Y, Liu G, et al. The Felodipine Event Reduction (FEVER) Study: a randomized long-term placebo-controlled trial in Chinese hypertensive patients. *J Hypertens* 2005;**23**:2157-72.
38. Wilhelmsen L, Berglund G, Elmfeldt D, et al. Beta-blockers versus diuretics in hypertensive men: main results from the HAPPHY trial. *J Hypertens* 1987;**5**:561-72.
39. Five-year findings of the hypertension detection and follow-up program. I. Reduction in mortality of persons with high blood pressure, including mild hypertension. Hypertension Detection and Follow-up Program Cooperative Group. *JAMA* 1979;**242**:2562-71.
40. Kasanuki H, Hagiwara N, Hosoda S, et al. Angiotensin II receptor blocker-based vs. non-angiotensin II receptor blocker-based therapy in patients with angiographically documented coronary artery disease and hypertension: the Heart Institute of Japan Candesartan Randomized Trial for Evaluation in Coronary Artery Disease (HIJ-CREATE). *Eur Heart J* 2009;**30**:1203-12. doi:10.1093/eurheartj/ehp101
41. Asayama K, Ohkubo T, Metoki H, et al. Cardiovascular outcomes in the first trial of antihypertensive therapy guided by self-measured home blood pressure. *Hypertens Res* 2012;**35**:1102-10. doi:10.1038/hr.2012.125
42. Investigators; HOPES, Yusuf S, Sleight P, et al. Effects of an angiotensin-converting-enzyme inhibitor, ramipril, on cardiovascular events in high-risk patients. *New Engl J Med* 2000;**342**:145-53. doi:10.1056/NEJM200001203420301
43. Lonn EM, Bosch J, Lopez-Jaramillo P, et al. Blood-Pressure Lowering in Intermediate-Risk Persons without Cardiovascular Disease. *New Engl J Med* 2016;**374**:2009-20. doi:10.1056/NEJMoa1600175
44. Hansson L, Zanchetti A, Carruthers SG, et al. Effects of intensive blood-pressure lowering and low-dose aspirin in patients with hypertension: principal results of the Hypertension Optimal Treatment (HOT) randomised trial. HOT Study Group. *Lancet* 1998;**351**:1755--62.
45. Beckett NS, Peters R, Fletcher AE, et al. Treatment of hypertension in patients 80 years of age or older. *New Engl J Med* 2008;**358**:1887-98. doi:10.1056/NEJMoa0801369

For peer review only

For peer review only - http://bmjopen.bmj.com/site/about/guidelines.xhtml

46. Lewis EJ, Hunsicker LG, Clarke WR, et al. Renoprotective effect of the angiotensin-receptor antagonist irbesartan in patients with nephropathy due to type 2 diabetes. *New Engl J Med* 2001;**345**:851-60. doi:10.1056/NEJMoa011303
47. Brown MJ, Palmer CR, Castaigne A, et al. Morbidity and mortality in patients randomised to double-blind treatment with a long-acting calcium-channel blocker or diuretic in the International Nifedipine GITS study: Intervention as a Goal in Hypertension Treatment (INSIGHT). *Lancet* 2000;**356**:366-72. doi:10.1016/S0140-6736(00)02527-7
48. Pepine CJ, Handberg EM, Cooper-DeHoff RM, et al. A calcium antagonist vs a non-calcium antagonist hypertension treatment strategy for patients with coronary artery disease. The International Verapamil-Trandolapril Study (INVEST): a randomized controlled trial. *JAMA* 2003;**290**:2805-16. doi:10.1001/jama.290.21.2805
49. Cardiovascular risk and risk factors in a randomized trial of treatment based on the beta-blocker oxprenolol: the International Prospective Primary Prevention Study in Hypertension (IPPPSH). The IPPPSH Collaborative Group. *J Hypertens* 1985;**3**:379-92.
50. JATOS Study Group. Principal results of the Japanese trial to assess optimal systolic blood pressure in elderly hypertensive patients (JATOS). *Hypertens Res* 2008;**31**:2115-27. doi:10.1291/hypres.31.2115
51. Yui Y, Sumiyoshi T, Kodama K, et al. Comparison of nifedipine retard with angiotensin converting enzyme inhibitors in Japanese hypertensive patients with coronary artery disease: the Japan Multicenter Investigation for Cardiovascular Diseases-B (JMIC-B) randomized trial. *Hypertens Res* 2004;**27**:181-91.
52. Dahlof B, Devereux RB, Kjeldsen SE, et al. Cardiovascular morbidity and mortality in the Losartan Intervention For Endpoint reduction in hypertension study (LIFE): a randomised trial against atenolol. *Lancet* 2002;**359**:995-1003. doi:10.1016/S0140-6736(02)08089-3
53. The Lopressor Intervention Trial: multicentre study of metoprolol in survivors of acute myocardial infarction. Lopressor Intervention Trial Research Group. *Eur Heart J* 1987;**8**:1056-64.
54. Borhani NO, Mercuri M, Borhani PA, et al. Final outcome results of the Multicenter Isradipine Diuretic Atherosclerosis Study (MIDAS). A randomized controlled trial. *JAMA* 1996;**276**:785-91.
55. Schrader J, Luders S, Kulschewski A, et al. Morbidity and Mortality After Stroke, Eprosartan Compared with Nitrendipine for Secondary Prevention: principal results of a prospective randomized controlled study (MOSES). *Stroke* 2005;**36**:1218-26. doi:10.1161/01.STR.0000166048.35740.a9
56. MRC trial of treatment of mild hypertension: principal results. Medical Research Council Working Party. *BMJ* 1985;**291**:97-104.
57. Medical Research Council trial of treatment of hypertension in older adults: principal results. MRC Working Party. *BMJ* 1992;**304**:405-12.
58. Improvement in prognosis of myocardial infarction by long-term beta-adrenoreceptor blockade using practolol. A multicentre international study. *BMJ* 1975;**3**:735-40.
59. Navigator Study Group, McMurray JJ, Holman RR, et al. Effect of valsartan on the incidence of diabetes and cardiovascular events. *New Engl J Med* 2010;**362**:1477-90. doi:10.1056/NEJMoa1001121
60. Dens JA, Desmet WJ, Coussement P, et al. Long term effects of nisoldipine on the progression of coronary atherosclerosis and the occurrence of clinical events: the NICOLE study. *Heart* 2003;**89**:887-92.
61. Randomized double-blind comparison of a calcium antagonist and a diuretic in elderly hypertensives. National Intervention Cooperative Study in Elderly Hypertensives Study Group. *Hypertension* 1999;**34**:1129-33.
62. Hansson L, Hedner T, Lund-Johansen P, et al. Randomised trial of effects of calcium antagonists compared with diuretics and beta-blockers on cardiovascular morbidity and mortality in hypertension: the Nordic Diltiazem (NORDIL) study. *Lancet* 2000;**356**:359-65.
63. ONTARGET Investigators, Yusuf S, Teo KK, et al. Telmisartan, ramipril, or both in patients at high risk for vascular events. *New Engl J Med* 2008;**358**:1547-59. doi:10.1056/NEJMoa0801317
64. Ogawa H, Kim-Mitsuyama S, Matsui K, et al. Angiotensin II receptor blocker-based therapy in Japanese elderly, high-risk, hypertensive patients. *Am J Med* 2012;**125**:981-90. doi:10.1016/j.amjmed.2011.12.010
65. Helgeland A. Treatment of mild hypertension: a five year controlled drug trial. The Oslo study. *Am J Med* 1980;**69**:725-32.
66. MacMahon S, Sharpe N, Gamble G, et al. Randomized, placebo-controlled trial of the angiotensin-converting enzyme inhibitor, ramipril, in patients with coronary or other occlusive arterial disease. PART-2 Collaborative Research Group. Prevention of Atherosclerosis with Ramipril. *J Am Coll Cardiol* 2000;**36**:438-43.
67. Ogihara T. Practitioner's Trial on the Efficacy of Antihypertensive Treatment in the Elderly Hypertension (The PATE-Hypertension Study) in Japan. *Am J Hypertens* 2000;**13**:461-7.
68. Liu L, Wang Z, Gong L, et al. Blood pressure reduction for the secondary prevention of stroke: a Chinese trial and a systematic review of the literature. *Hypertens Research* 2009;**32**:1032-40. doi:10.1038/hr.2009.139
69. Braunwald E, Domanski MJ, Fowler SE, et al. Angiotensin-converting-enzyme inhibition in stable coronary artery disease. *New Engl J Med* 2004;**351**:2058-68. doi:10.1056/NEJMoa042739
70. Luders S, Schrader J, Berger J, et al. The PHARAO study: prevention of hypertension with the angiotensin-converting enzyme inhibitor ramipril in patients with high-normal blood pressure: a prospective, randomized, controlled prevention trial of the German Hypertension League. *J Hypertens* 2008;**26**:1487-96. doi:10.1097/HJH.0b013e3282ff8864

For peer review only

71. Asselbergs FW, Diercks GF, Hillege HL, et al. Effects of fosinopril and pravastatin on cardiovascular events in subjects with microalbuminuria. *Circulation* 2004;**110**:2809-16. doi:10.1161/01.CIR.0000146378.65439.7A
72. Pitt B, Byington RP, Furberg CD, et al. Effect of amlodipine on the progression of atherosclerosis and the occurrence of clinical events. PREVENT Investigators. *Circulation* 2000;**102**:1503-10.
73. Yusuf S, Diener HC, Sacco RL, et al. Telmisartan to prevent recurrent stroke and cardiovascular events. *New Engl J Med* 2008**;359**:1225-37. doi:10.1056/NEJMoa0804593
74. Randomised trial of a perindopril-based blood-pressure-lowering regimen among 6105 individuals with previous stroke or transient ischaemic attack. *Lancet* 2001;**358**:1033-41. doi:10.1016/s0140-6736(01)06178-5
75. Pitt B, O'Neill B, Feldman R, et al. The QUinapril Ischemic Event Trial (QUIET): evaluation of chronic ACE inhibitor therapy in patients with ischemic heart disease and preserved left ventricular function. *Am J Cardiol* 2001;**87**:1058-63.
76. Brenner BM, Cooper ME, de Zeeuw D, et al. Effects of losartan on renal and cardiovascular outcomes in patients with type 2 diabetes and nephropathy. *New Engl J Med* 2001;**345**:861-9. doi:10.1056/NEJMoa011161
77. Haller H, Ito S, Izzo JL, Jr., et al. Olmesartan for the delay or prevention of microalbuminuria in type 2 diabetes. *New Engl J Med* 2011;**364**:907-17. doi:10.1056/NEJMoa1007994
78. Lithell H, Hansson L, Skoog I, et al. The Study on Cognition and Prognosis in the Elderly (SCOPE): principal results of a randomized double-blind intervention trial. *J Hypertens* 2003;**21**:875-86. doi:10.1097/01.hjh.0000059028.82022.89
79. Malacco E, Mancia G, Rappelli A, et al. Treatment of isolated systolic hypertension: the SHELL study results. *Blood Pressure* 2003;**12**:160-7.
80. Prevention of stroke by antihypertensive drug treatment in older persons with isolated systolic hypertension. Final results of the Systolic Hypertension in the Elderly Program (SHEP). SHEP Cooperative Research Group. *JAMA* 1991;**265**:3255-64.
81. SPRINT Research Group, Wright JT, Jr., Williamson JD, et al. A Randomized Trial of Intensive versus Standard Blood-Pressure Control. *New Engl J Med* 2015;**373**:2103-16. doi:10.1056/NEJMoa1511939
82. S. P. S. Study Group, Benavente OR, Coffey CS, et al. Blood-pressure targets in patients with recent lacunar stroke: the SPS3 randomised trial. *Lancet* 2013;**382**:507-15. doi:10.1016/S0140-6736(13)60852-1
83. Gong L, Zhang W, Zhu Y, et al. Shanghai trial of nifedipine in the elderly (STONE). *J Hypertens* 1996;**14**:1237-45.
84. Dahlof B, Lindholm LH, Hansson L, et al. Morbidity and mortality in the Swedish Trial in Old Patients with Hypertension (STOP-Hypertension). *Lancet* 1991;**338**:1281-5.
85. Hansson L, Lindholm LH, Ekbom T, et al. Randomised trial of old and new antihypertensive drugs in elderly patients: cardiovascular mortality and morbidity the Swedish Trial in Old Patients with Hypertension-2 study. *Lancet* 1999;**354**:1751-6.
86. Sun M, Zhou H, Jia Z. [Prevention and treatment of stroke after hypertension for ten years in Hunan Province]. *Zhonghua nei ke za zhi* 1997;**36**:312-4.
87. Liu L, Wang JG, Gong L, et al. Comparison of active treatment and placebo in older Chinese patients with isolated systolic hypertension. Systolic Hypertension in China (Syst-China) Collaborative Group. *J Hypertens* 1998;**16**:1823-9.
88. Staessen JA, Fagard R, Thijs L, et al. Randomised double-blind comparison of placebo and active treatment for older patients with isolated systolic hypertension. The Systolic Hypertension in Europe (Syst-Eur) Trial Investigators. *Lancet* 1997;**350**:757-64.
89. Taylor SH, Silke B, Ebbutt A, et al. A long-term prevention study with oxprenolol in coronary heart disease. *New Engl J Med* 1982;**307**:1293-301. doi:10.1056/NEJM198211183072101
90. Eriksson S, Olofsson BO, Wester PO, et al. Atenolol in Secondary Prevention after Stroke. *Cerebrovasc Dis* 1995;**5**:21-25. doi:https://doi.org/10.1159/000107813
91. Neaton JD, Grimm RH, Jr., Prineas RJ, et al. Treatment of Mild Hypertension Study. Final results. Treatment of Mild Hypertension Study Research Group. *JAMA* 1993;**270**:713-24.
92. Telmisartan Randomised AssessmeNt Study in A. C. E. iNtolerant subjects with cardiovascular Disease Investigators, Yusuf S, Teo K, et al. Effects of the angiotensin-receptor blocker telmisartan on cardiovascular events in high-risk patients intolerant to angiotensin-converting enzyme inhibitors: a randomised controlled trial. *Lancet* 2008;**372**:1174-83. doi:10.1016/S0140-6736(08)61242-8
93. Julius S, Nesbitt SD, Egan BM, et al. Feasibility of treating prehypertension with an angiotensin-receptor blocker. *The New Engl J Med* 2006;**354**:1685-97. doi:10.1056/NEJMoa060838
94. Tight blood pressure control and risk of macrovascular and microvascular complications in type 2 diabetes: UKPDS 38. UK Prospective Diabetes Study Group. *BMJ* 1998;**317**:703-13.
95. Fried LF, Emanuele N, Zhang JH, et al. Combined angiotensin inhibition for the treatment of diabetic nephropathy. *The New Engl J Med* 2013;**369**:1892-903. doi:10.1056/NEJMoa1303154
96. Ogihara T, Saruta T, Rakugi H, et al. Target blood pressure for treatment of isolated systolic hypertension in the elderly: valsartan in elderly isolated systolic hypertension study. *Hypertension* 2010**;56**:196-202. doi:10.1161/HYPERTENSIONAHA.109.146035

For peer review only - http://bmjopen.bmj.com/site/about/guidelines.xhtml

97. Julius S, Kjeldsen SE, Weber M, et al. Outcomes in hypertensive patients at high cardiovascular risk treated with regimens based on valsartan or amlodipine: the VALUE randomised trial. *Lancet* 2004;**363**:2022-31. doi:10.1016/s0140-6736(04)16451-9
98. Rosei EA, Dal Palu C, Leonetti G, et al. Clinical results of the Verapamil in Hypertension and Atherosclerosis Study. VHAS Investigators. *J Hypertens* 1997;**15**:1337-44.
99. Wei Y, Jin Z, Shen G, et al. Effects of intensive antihypertensive treatment on Chinese hypertensive patients older than 70 years. *J Clin Hypertens* 2013;**15**:420-7. doi:10.1111/jch.12094

For peer review only

Supplementary table 4. Variables to be sought from participating trials.

Data	Variables
Study level data	Region<br>Treatment/Comparison groups<br>Study period / Duration of follow-up<br>Randomisation method/Treatment allocation<br>Outcome ascertainment<br>Early stopping and reasons<br>Funding source
Participant level data	Baseline characteristics<br>Sex, age, ethnicity, lifestyle (e.g. smoking, alcohol intake), past medical history (e.g. diabetes, cardiovascular disease, chronic renal disease), drug treatment (e.g. hypertension, dyslipidaemia, cardiovascular disease) weight, height, systolic and diastolic blood pressure, blood and urine measurements (e.g. lipids, glucose homestasis, renal function)<br><br>Follow-up variables (after randomisation)<br>Outcomes and relevant dates (e.g. cardiovascular events, acute renal injury / renal replacement therapy, cancer, fractures, diabetes, retinopathy, neurodegenerative conditions, serious adverse events)<br>Vital status (and cause of death) on last follow-up<br>Clinical measures and relevant dates (e.g. weight, height, systolic and diastolic blood pressure, blood and urine measurements)<br>Changes in treatment (e.g. discontinuation) and date

For peer review only

AMSTAR 2: a critical appraisal tool for systematic reviews that include randomised or non-randomised studies of healthcare interventions, or both

1. Did the research questions and inclusion criteria for the review include the components of PICO?

For Yes:
- ☑ Population
- ☑ Intervention
- ☑ Comparator group
- ☑ Outcome

Optional (recommended)
- ☑ Timeframe for follow-up

- ☑ Yes
- ☐ No

2. Did the report of the review contain an explicit statement that the review methods were established prior to the conduct of the review and did the report justify any significant deviations from the protocol?

For Partial Yes:
The authors state that they had a written protocol or guide that included ALL the following:
- ☑ review question(s)
- ☑ a search strategy
- ☑ inclusion/exclusion criteria
- ☑ a risk of bias assessment

For Yes:
As for partial yes, plus the protocol should be registered and should also have specified:
- ☑ a meta-analysis/synthesis plan, if appropriate, *and*
- ☑ a plan for investigating causes of heterogeneity
- ☐ justification for any deviations from the protocol

- ☑ Yes
- ☐ Partial Yes
- ☐ No

3. Did the review authors explain their selection of the study designs for inclusion in the review?

For Yes, the review should satisfy ONE of the following:
- ☑ *Explanation for* including only RCTs
- ☐ OR *Explanation for* including only NRSI
- ☐ OR *Explanation for* including both RCTs and NRSI

- ☑ Yes
- ☐ No

4. Did the review authors use a comprehensive literature search strategy?

For Partial Yes (all the following):
- ☑ searched at least 2 databases (relevant to research question)
- ☑ provided key word and/or search strategy
- ☑ justified publication restrictions (e.g. language)

For Yes, should also have (all the following):
- ☑ searched the reference lists / bibliographies of included studies
- ☑ searched trial/study registries
- ☑ included/consulted content experts in the field
- ☑ where relevant, searched for grey literature
- ☑ conducted search within 24 months of completion of the review

- ☑ Yes
- ☐ Partial Yes
- ☐ No

5. Did the review authors perform study selection in duplicate?

For Yes, either ONE of the following:
- ☑ at least two reviewers independently agreed on selection of eligible studies and achieved consensus on which studies to include
- ☐ OR two reviewers selected a sample of eligible studies and achieved good agreement (at least 80 percent), with the remainder selected by one reviewer.

- ☑ Yes
- ☐ No

For peer review only

AMSTAR 2: a critical appraisal tool for systematic reviews that include randomised or non-randomised studies of healthcare interventions, or both

This section onwards are currently not relevant to the protocol.

6. Did the review authors perform data extraction in duplicate?

For Yes, either ONE of the following:	
☐ at least two reviewers achieved consensus on which data to extract from included studies<br>☐ OR two reviewers extracted data from a sample of eligible studies <u>and</u> achieved good agreement (at least 80 percent), with the remainder extracted by one reviewer.	☐ Yes<br>☐ No

7. Did the review authors provide a list of excluded studies and justify the exclusions?

For Partial Yes:	For Yes, must also have:	
☐ provided a list of all potentially relevant studies that were read in full-text form but excluded from the review	☐ Justified the exclusion from the review of each potentially relevant study	☐ Yes<br>☐ Partial Yes<br>☐ No

8. Did the review authors describe the included studies in adequate detail?

For Partial Yes (ALL the following):	For Yes, should also have ALL the following:	
☐ described populations<br>☐ described interventions<br>☐ described comparators<br>☐ described outcomes<br>☐ described research designs	☐ described population in detail<br>☐ described intervention in detail (including doses where relevant)<br>☐ described comparator in detail (including doses where relevant)<br>☐ described study's setting<br>☐ timeframe for follow-up	☐ Yes<br>☐ Partial Yes<br>☐ No

9. Did the review authors use a satisfactory technique for assessing the risk of bias (RoB) in individual studies that were included in the review?

**RCTs**<br>For Partial Yes, must have assessed RoB from	For Yes, must also have assessed RoB from:	
☐ unconcealed allocation, *and*<br>☐ lack of blinding of patients and assessors when assessing outcomes (unnecessary for objective outcomes such as all-cause mortality)	☐ allocation sequence that was not truly random, *and*<br>☐ selection of the reported result from among multiple measurements or analyses of a specified outcome	☐ Yes<br>☐ Partial Yes<br>☐ No<br>☐ Includes only NRSI
**NRSI**<br>For Partial Yes, must have assessed RoB:	For Yes, must also have assessed RoB:	
☐ from confounding, *and*<br>☐ from selection bias	☐ methods used to ascertain exposures and outcomes, *and*<br>☐ selection of the reported result from among multiple measurements or analyses of a specified outcome	☐ Yes<br>☐ Partial Yes<br>☐ No<br>☐ Includes only RCTs

10. Did the review authors report on the sources of funding for the studies included in the review?

For Yes	
☐ Must have reported on the sources of funding for individual studies included in the review. Note: Reporting that the reviewers looked for this information but it was not reported by study authors also qualifies	☐ Yes<br>☐ No

For peer review only

AMSTAR 2: a critical appraisal tool for systematic reviews that include randomised or non-randomised studies of healthcare interventions, or both

11. If meta-analysis was performed did the review authors use appropriate methods for statistical combination of results?

RCTs
For Yes:

- ☐ The authors justified combining the data in a meta-analysis
  - ☐ AND they used an appropriate weighted technique to combine study results and adjusted for heterogeneity if present.
  - ☐ AND investigated the causes of any heterogeneity

☐ Yes
☐ No
☐ No meta-analysis conducted

For NRSI
For Yes:

- ☐ The authors justified combining the data in a meta-analysis
  - ☐ AND they used an appropriate weighted technique to combine study results, adjusting for heterogeneity if present
  - ☐ AND they statistically combined effect estimates from NRSI that were adjusted for confounding, rather than combining raw data, or justified combining raw data when adjusted effect estimates were not available
  - ☐ AND they reported separate summary estimates for RCTs and NRSI separately when both were included in the review

☐ Yes
☐ No
☐ No meta-analysis conducted

12. If meta-analysis was performed, did the review authors assess the potential impact of RoB in individual studies on the results of the meta-analysis or other evidence synthesis?

For Yes:

- ☐ included only low risk of bias RCTs
- ☐ OR, if the pooled estimate was based on RCTs and/or NRSI at variable RoB, the authors performed analyses to investigate possible impact of RoB on summary estimates of effect.

☐ Yes
☐ No
☐ No meta-analysis conducted

13. Did the review authors account for RoB in individual studies when interpreting/ discussing the results of the review?

For Yes:

- ☐ included only low risk of bias RCTs
- ☐ OR, if RCTs with moderate or high RoB, or NRSI were included the review provided a discussion of the likely impact of RoB on the results

☐ Yes
☐ No

14. Did the review authors provide a satisfactory explanation for, and discussion of, any heterogeneity observed in the results of the review?

For Yes:

- ☐ There was no significant heterogeneity in the results
- ☐ OR if heterogeneity was present the authors performed an investigation of sources of any heterogeneity in the results and discussed the impact of this on the results of the review

☐ Yes
☐ No

15. If they performed quantitative synthesis did the review authors carry out an adequate investigation of publication bias (small study bias) and discuss its likely impact on the results of the review?

For Yes:

- ☐ performed graphical or statistical tests for publication bias and discussed the likelihood and magnitude of impact of publication bias

☐ Yes
☐ No
☐ No meta-analysis conducted

For peer review only

AMSTAR 2: a critical appraisal tool for systematic reviews that include randomised or non-randomised studies of healthcare interventions, or both

16. Did the review authors report any potential sources of conflict of interest, including any funding they received for conducting the review?

For Yes:	
☐ The authors reported no competing interests OR	☐ Yes
☐ The authors described their funding sources and how they managed potential conflicts of interest	☐ No

To cite this tool: Shea BJ, Reeves BC, Wells G, Thuku M, Hamel C, Moran J, Moher D, Tugwell P, Welch V, Kristjansson E, Henry DA. AMSTAR 2: a critical appraisal tool for systematic reviews that include randomised or non-randomised studies of healthcare interventions, or both. BMJ. 2017 Sep 21;358:j4008.

For peer review only
